# Supplementary material for: Genomic and Chemical Decryption of the Bacteroidetes Phylum for Its Potential to Biosynthesize Natural Products
Source: Microbiol Spectr. 2022 Apr 20;10(3):e02479-21. doi: 10.1128/spectrum.02479-21 (PMC9248904; doi:10.1128/spectrum.02479-21)
Supplement: SUPPLEMENTAL FILE 1 — Supplemental material. Download spectrum.02479-21-s001.pdf, PDF file, 7.7 MB [file spectrum.02479-21-s001.pdf]

Supplementary Materials for

Genomic and chemical decryption of the Bacteroidetes phylum for its potential to biosynthesize natural products

Stephan Brinkmann[1], Michael Kurz[2], Maria A. Patras[1], Christoph Hartwig[1], Michael Marner[1], Benedikt Leis[1], André Billion[1], Yolanda Kleiner[1], Armin Bauer[2], Luigi Toti[2], Christoph Pöverlein[2], Peter E. Hammann[3], Andreas Vilcinskas[1,4], Jens Glaeser[1,3], Marius S. Spohn[1*] and Till F. Schäberle[1,4*]

*Correspondence: Marius S. Spohn; Till F. Schäberle

Email: marius.spohn@ime.fraunhofer.de; till.f.schaeberle@agrar.uni-giessen.de

This PDF file includes:

R-script for chemotype-barcoding matrix
Figs. S1 to S39
Tables S1 to S7
Synthesis of β-hydroxyamino acids (Phe, Ile, Asp)

Other Supplementary Materials for this manuscript include the following:

Table S8

Table of Contents

1. R-script for chemotype-barcoding matrix 3

2. Supplementary Figures 1 to 39 4

3. Supplementary Tables 1 to 7 29

4. Synthesis of β-hydroxyamino acids (Phe, Ile, Asp) 38

4.1. Synthetic procedures and compound characterization 38

4.1.1. General Methods 38

4.1.2. Preparation of the β-hydroxyaspartic acids 39

4.1.3. Preparation of the β-hydroxyphenylalanines 45

4.1.4. Preparation of the β-hydroxyisoleucines 55

4.2. NMR spectra of synthetic β-hydroxyamino acid intermediates 64

4.3. Determination of the enantiomeric excesses (*ee*) of synthetic β-hydroxyamino acid intermediates 80

1. R-script for chemotype-barcoding matrix

```
library(readr)
library(reshape)
library(ggplot2)

setwd("E:/Stephan/") # Working directory
experiment<-"2020-12-16_chitinophaga_v1"  #Experiment name
project<-(paste(c(experiment,""), collapse=""))

presencetable.gesamt<-as.matrix(read_tsv(paste(c(project,".txt"), collapse=""),col_names=TRUE,
col_types = NULL, na = c("", "NA"), trim_ws = FALSE, skip = 0, n_max = Inf, progress =
show_progress(), skip_empty_rows = TRUE))  # load project file
#presencetable.transformed<-t(presencetable.gesamt)
n<-ncol(presencetable.gesamt)
presencetable.ges<-presencetable.gesamt[,2:n]
names<-presencetable.gesamt[,1]
rownames(presencetable.ges)<-names
melt.data<-melt(presencetable.ges)
colnames(melt.data)<-c("Bucket","Condition","Value")
png(filename=paste(c(project,"_test.png"), collapse=""),
   width = 50*400,
   height = 50*400,
   res = 1000,
   pointsize = 5)
qplot(data=melt.data,
        x=Bucket,
        y=Condition,
        fill=factor(Value),
      #   geom="tile")+scale_fill_manual(values=c("more than one"="black", "R2A"="green",
"5065"="blue", "3018"="orange","3021"="pink","5294"="brown","empty"="white", "isolated"="red" ))
         geom="tile")+scale_fill_manual(values=c("A - more than one"="black", "F - R2A"="green", "D -
5065"="blue", "B - 3018"="orange","E - 3021"="pink","C - 5294"="brown","G - empty"="white", "H -
isolated"="red" ))
dev.off()
```

2. Supplementary Figures 1 to 39

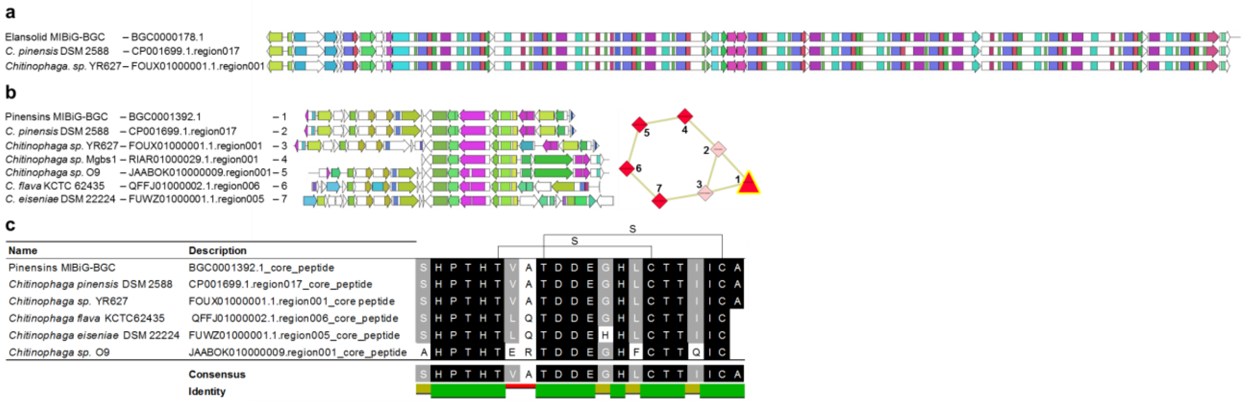

Fig. S1. Gene cluster families (GCF) of the elansolids and pinensins color coded by PFAM domains. a, Reference biosynthetic gene cluster of the elansolids deposited at MIBiG and two further identical BGCs identified on genomes of different strains. **b**, Reference biosynthetic gene cluster of the pinensins deposited at MIBiG and six modified derivatives found on genomes of different strains are depicted with the PFAM domains color coded and the gene cluster family shown to the right. BiG-SCAPE results with a cutoff of 0.6. **c**, Clustal W alignment (111) of the pinensin core peptide with those found in its GCF. No core peptide was found on the contig of *Chitinophaga sp.* Mgbs1 (RIAR01000029.1.region001).

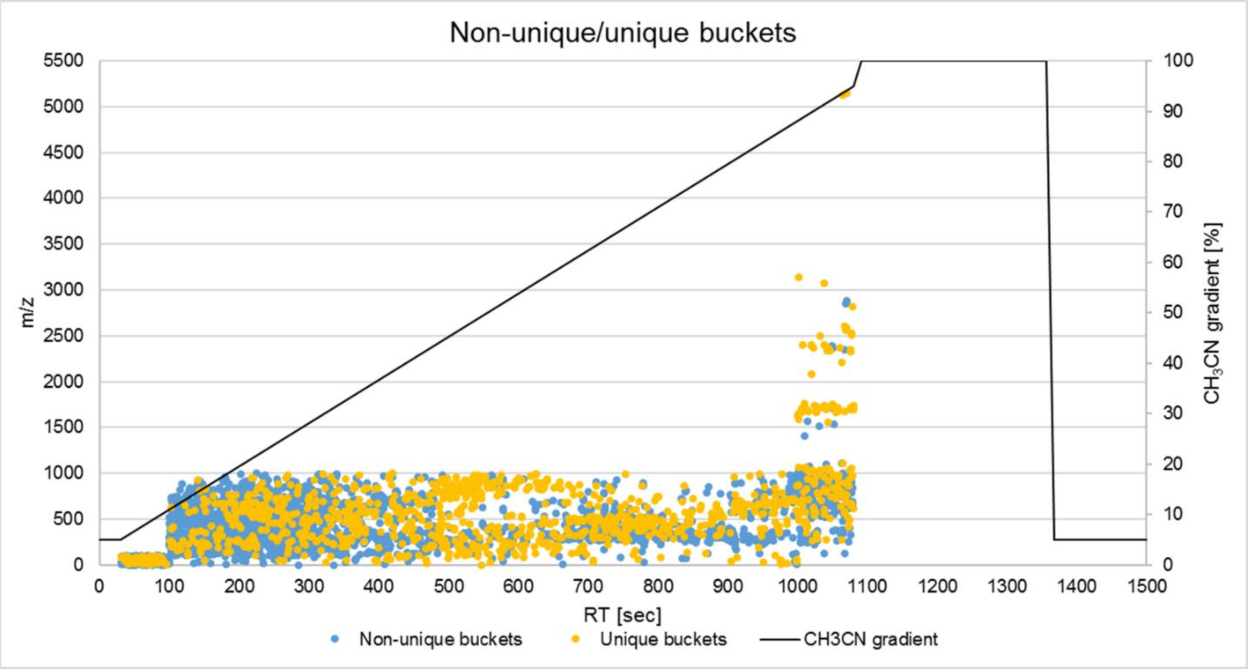

Fig. S2 Distribution of non-unique/unique buckets revealed by metablomics analysis shown as 2D-scatterplot. RT, retention time in seconds; *m/z*, mass to charge ratio; CH_3CN, acetonitrile.

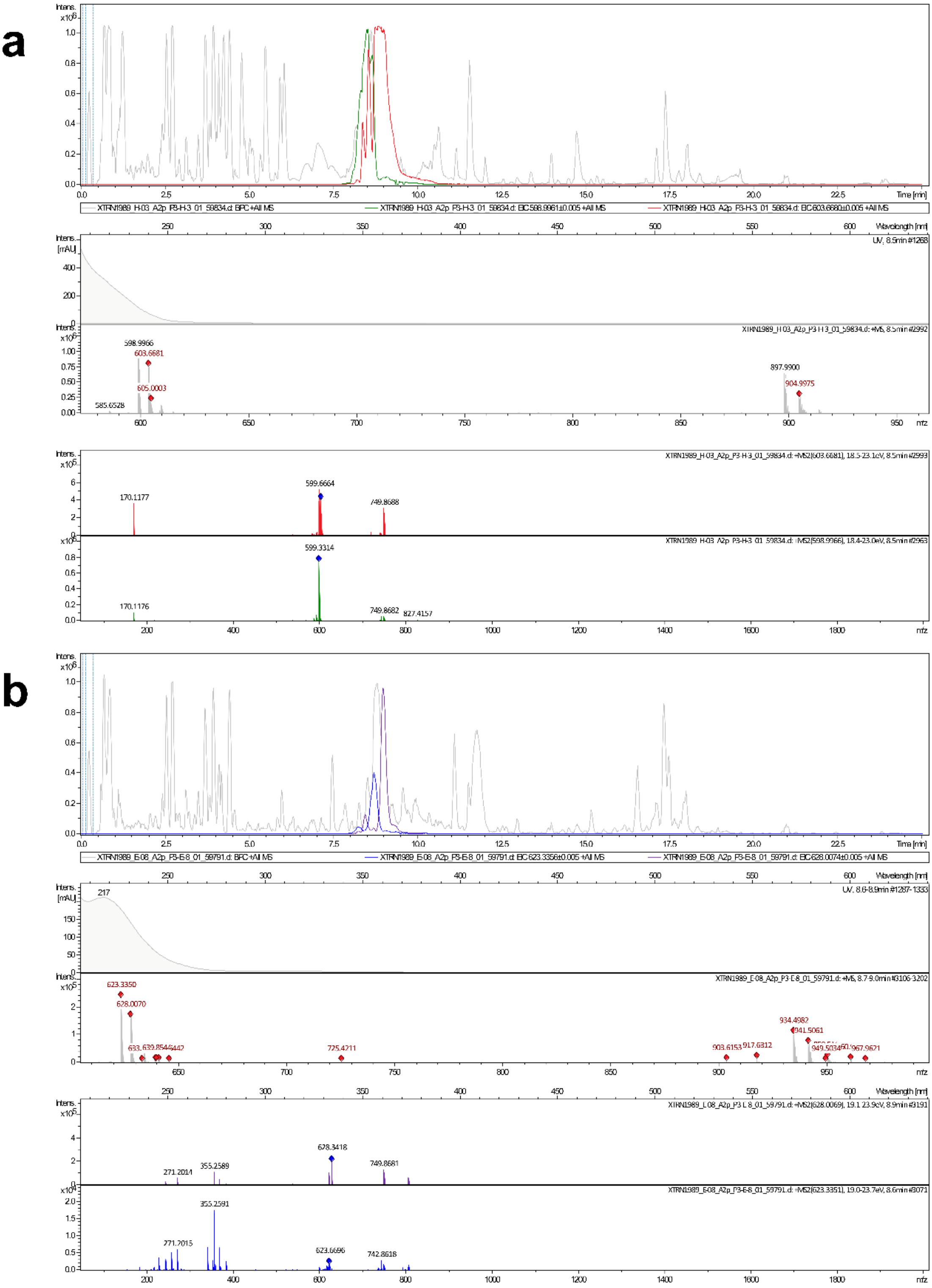

Fig. S3 (**A**) Analytics (extracted ion chromatograms and MS/MS fragments) of chitinopeptin A with *m/z* 603.6680 $[M+3H]^{3+}$ (red) and chitinopeptin B with m/z 598.6661 $[M+3H]^{3+}$ (green), (**B**) and chitinopeptin C1+C2 with *m/z* 628.0074 $[M+3H]^{3+}$ (purple) and chitinopeptin D1+D2 with *m/z* 623.3356 $[M+3H]^{3+}$ (blue).

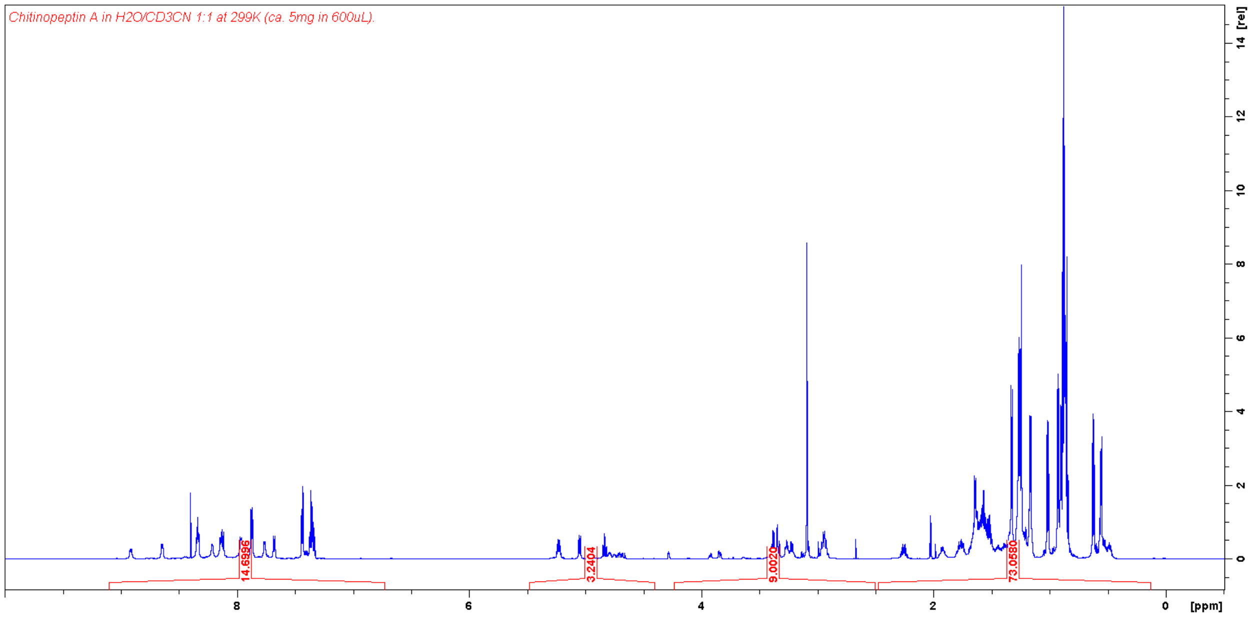

Fig. S4 ^{1}H NMR spectrum (700 MHz, H_2O/CD_3CN 1:1) of chitinopeptin A.

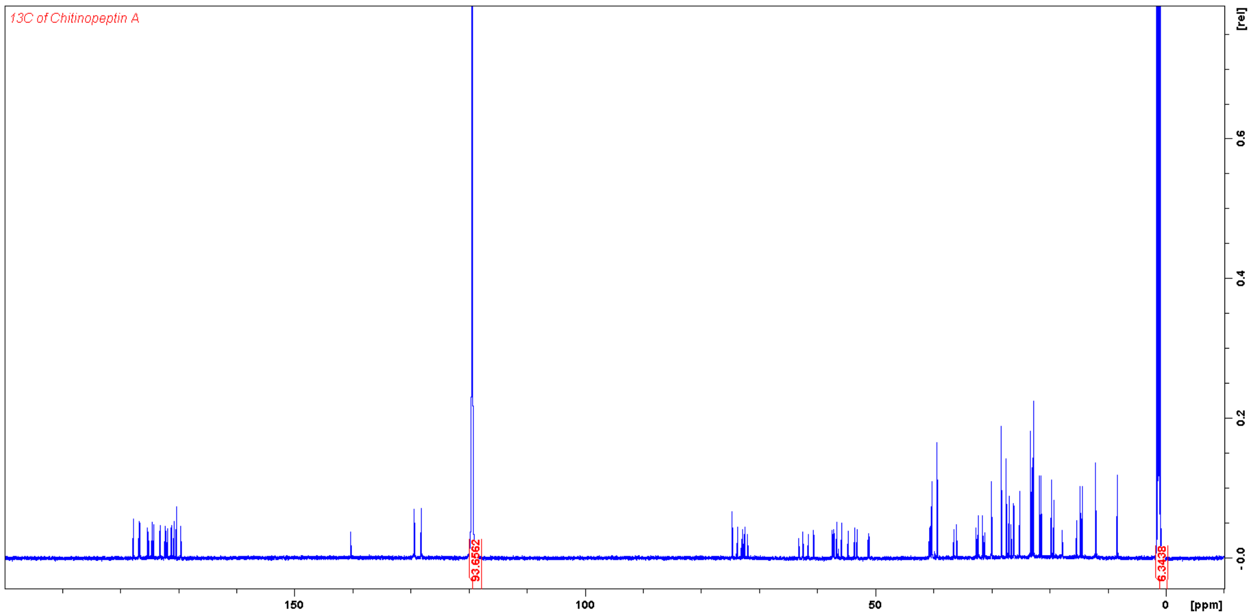

Fig. S5 ^{13}C NMR spectrum (175 MHz, H_2O/CD_3CN 1:1) of chitinopeptin A.

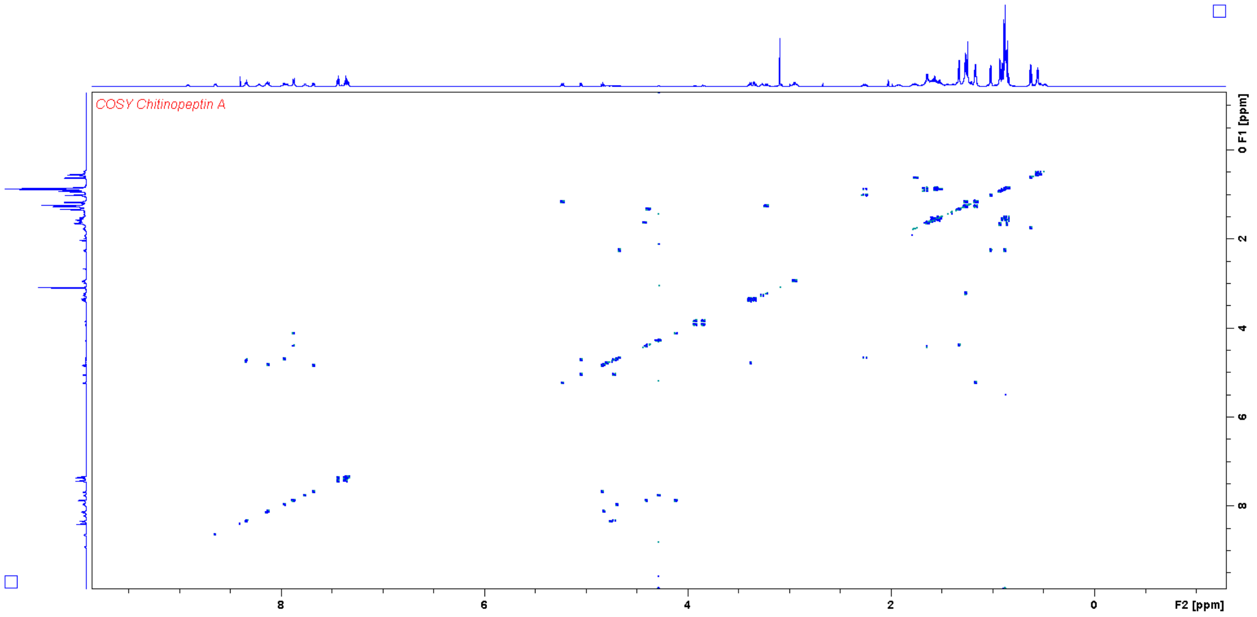

Fig. S6 COSY NMR spectrum (700 MHz, H_2O/CD_3CN 1:1) of chitinopeptin A.

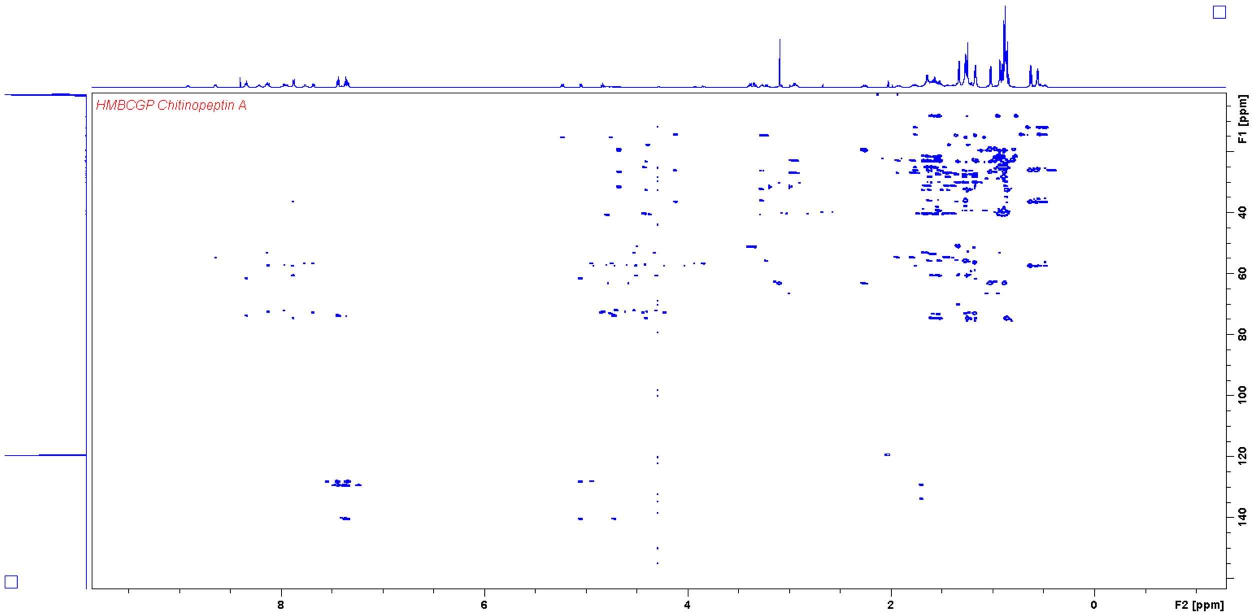

Fig. S7 HMBC NMR spectrum (700 MHz, H_2O/CD_3CN 1:1) of chitinopeptin A.

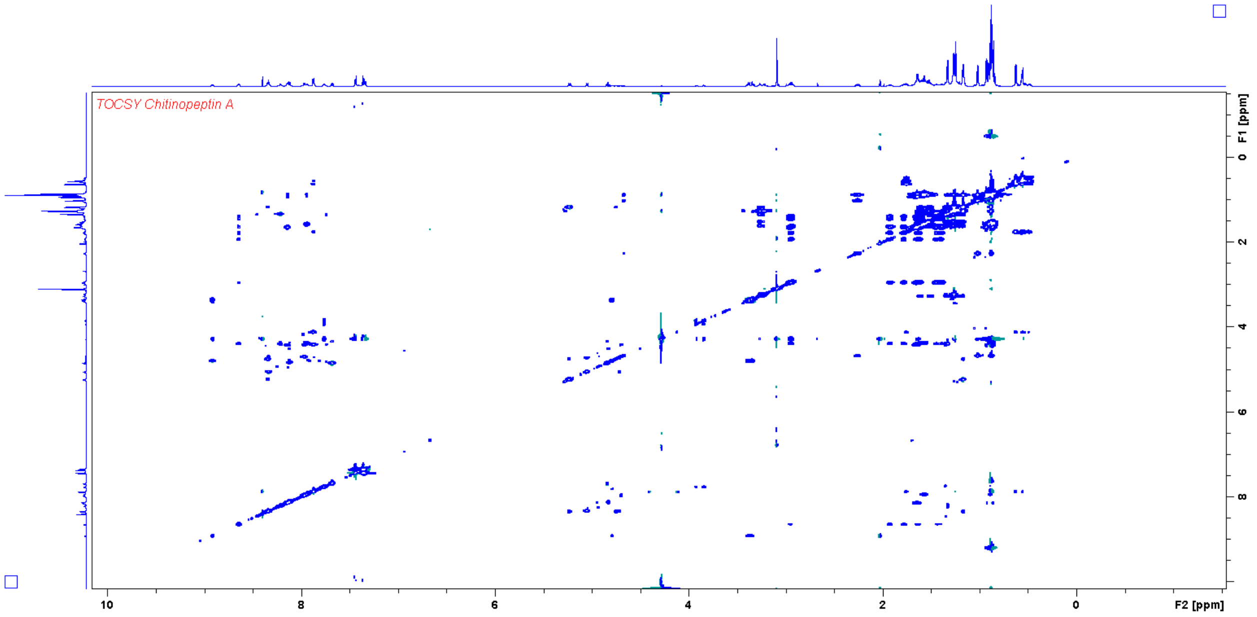

Fig. S8 TOCSY NMR spectrum (700 MHz, H_2O/CD_3CN 1:1) of chitinopeptin A.

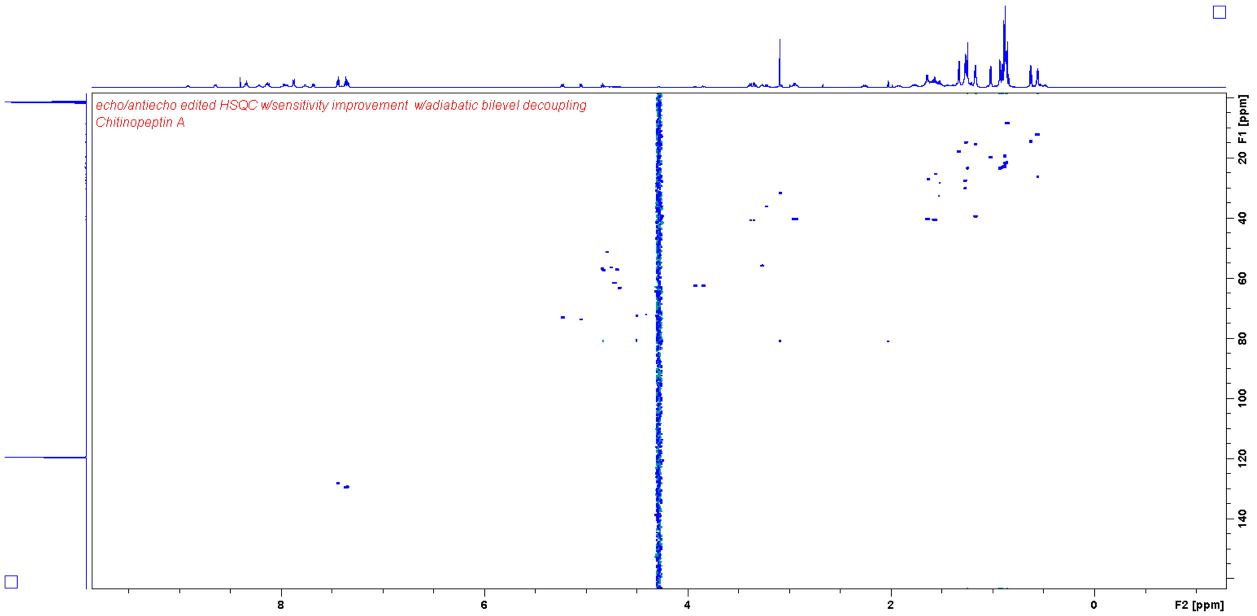

Fig. S9 HSQC NMR spectrum (700 MHz, H_2O/CD_3CN 1:1) of chitinopeptin A.

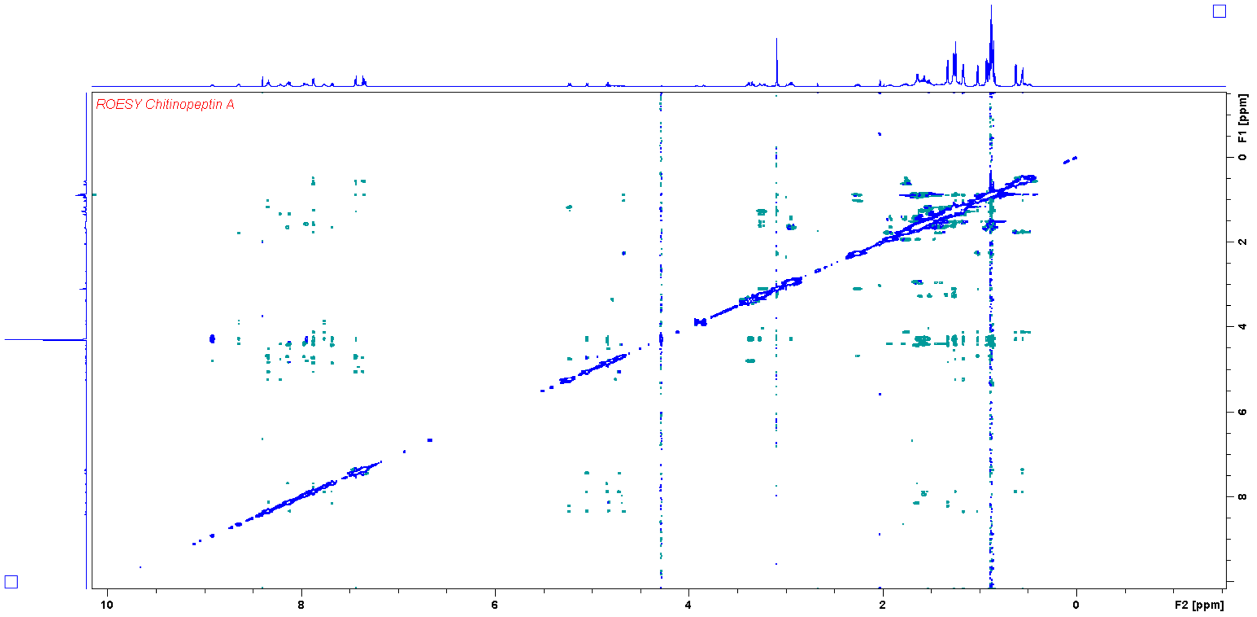

Fig. S10 ROESY NMR spectrum (700 MHz, H_2O/CD_3CN 1:1) of chitinopeptin A.

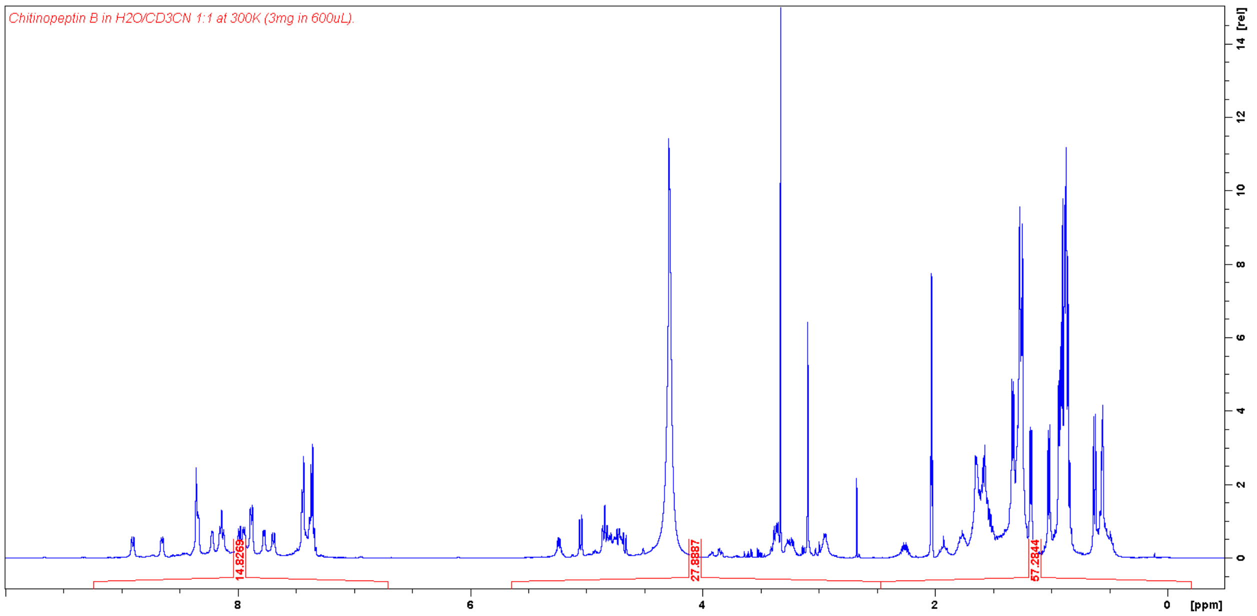

Fig. S11 ^{1}H NMR spectrum (500 MHz, H_2O/CD_3CN 1:1) of chitinopeptin B.

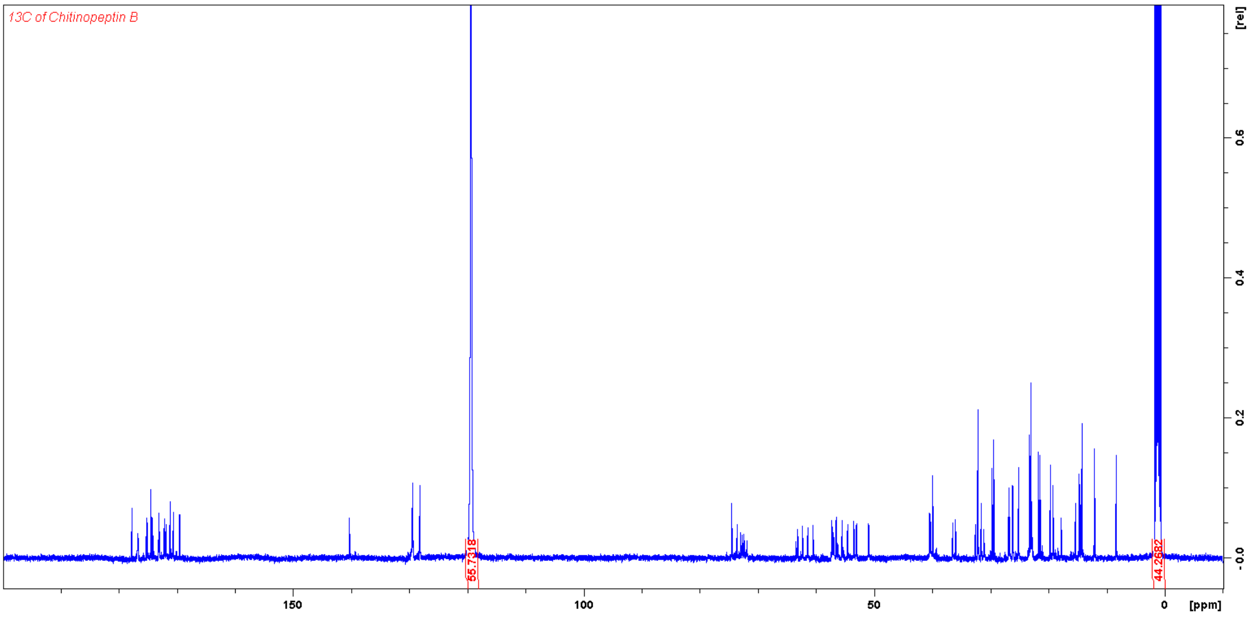

Fig. S12 ^{13}C NMR spectrum (125 MHz, D_2O/CD_3CN 1:1) of chitinopeptin B.

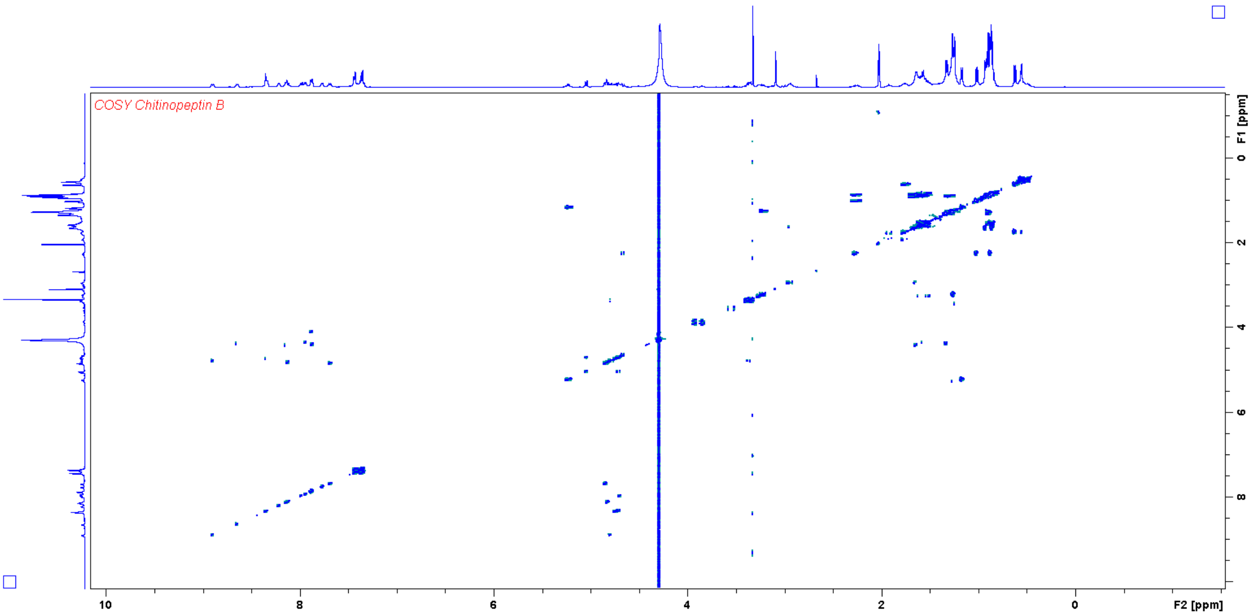

Fig. S13 COSY NMR spectrum (500 MHz, H_2O/CD_3CN 1:1) of chitinopeptin B.

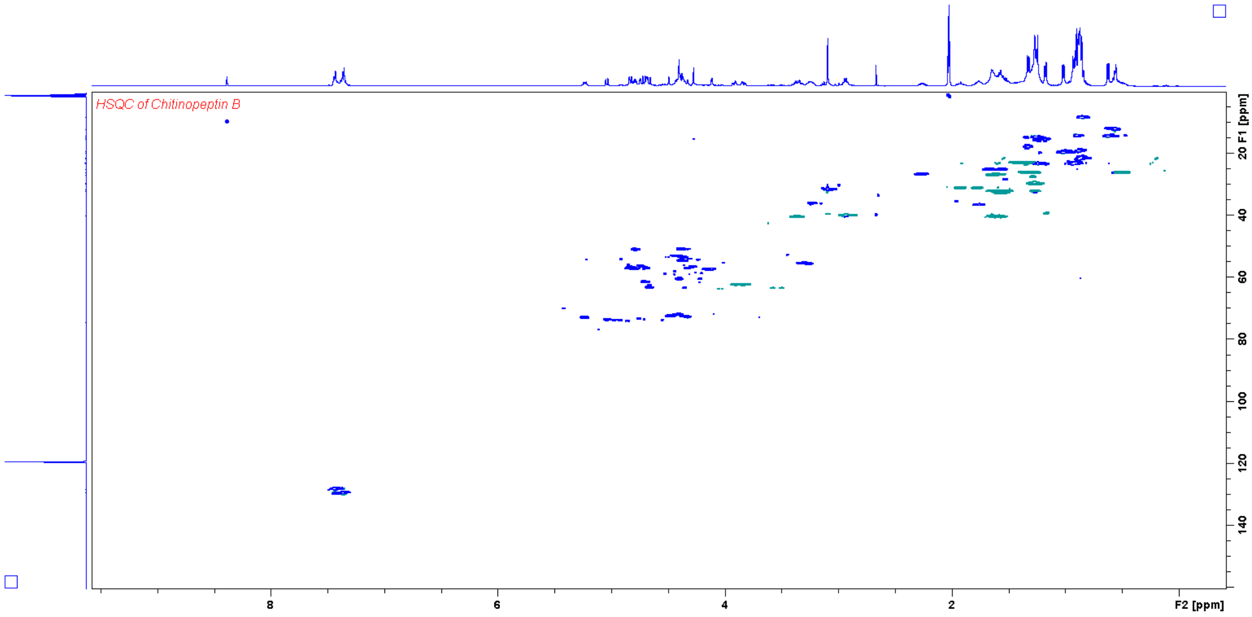

Fig. S14 HSQC NMR spectrum (500 MHz, D_2O/CD_3CN 1:1) of chitinopeptin B.

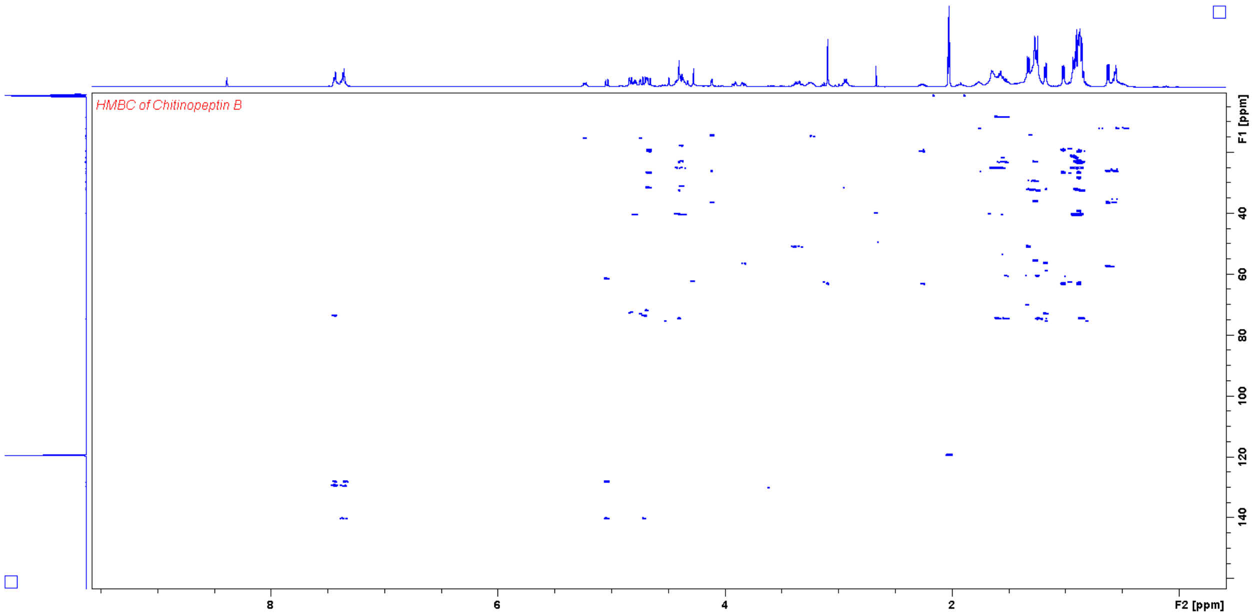

Fig. S15 HMBC NMR spectrum (500 MHz, D_2O/CD_3CN 1:1) of chitinopeptin B.

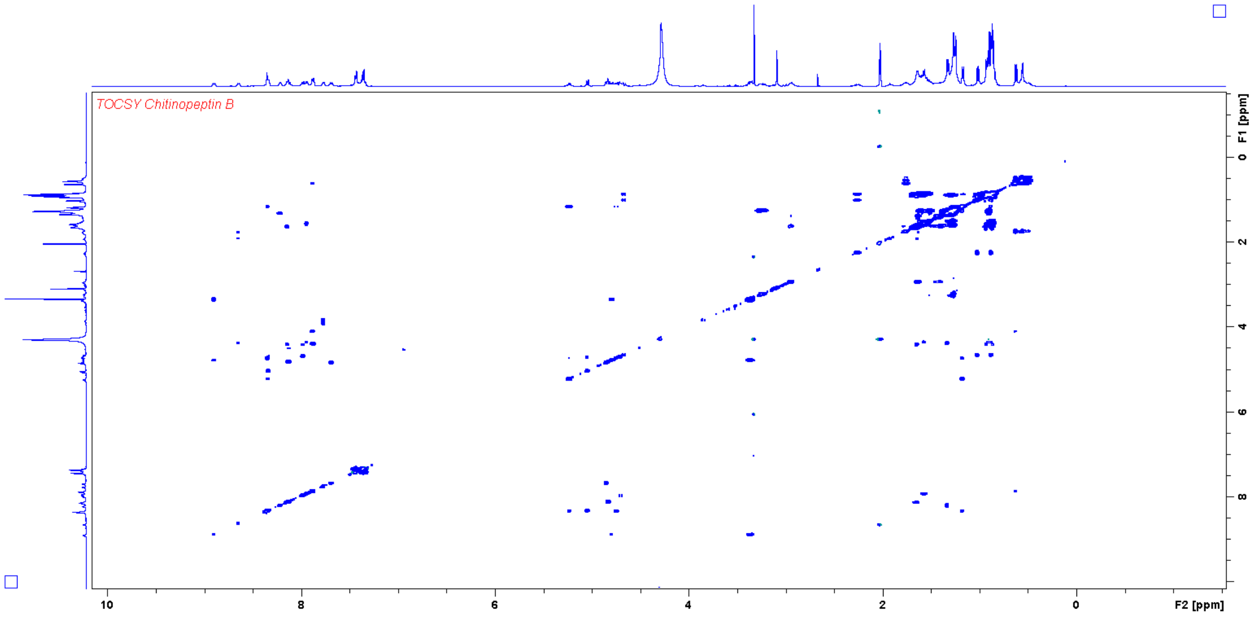

Fig. S16 TOCSY NMR spectrum (500 MHz, H_2O/CD_3CN 1:1) of chitinopeptin B.

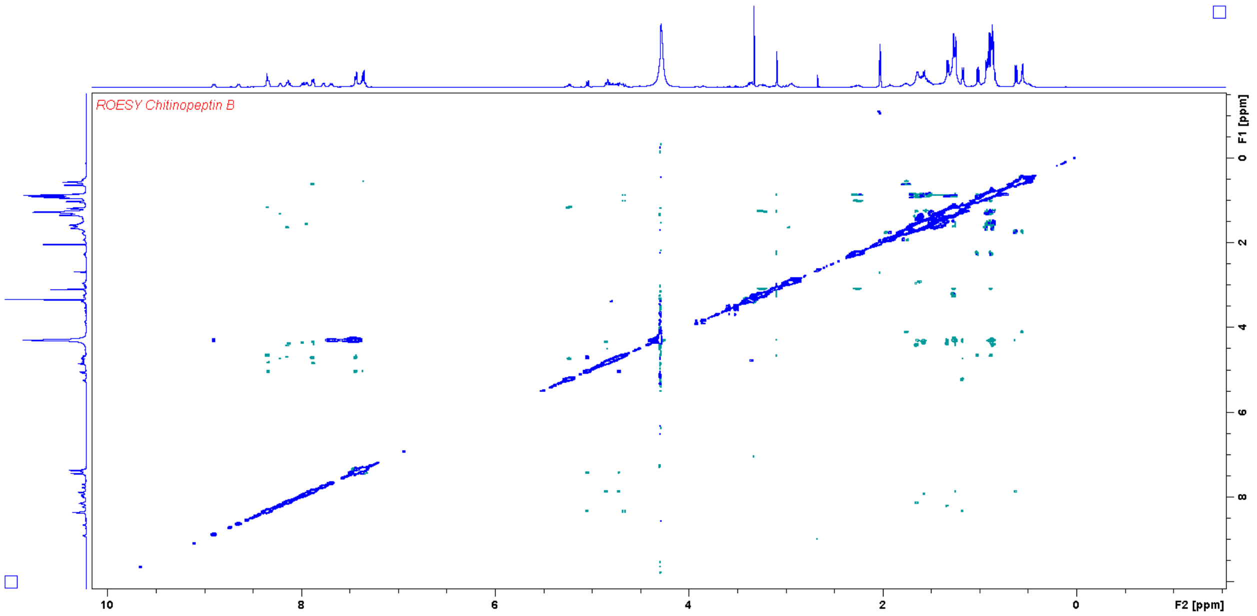

Fig. S17 ROESY NMR spectrum (500 MHz, H_2O/CD_3CN 1:1) of chitinopeptin B.

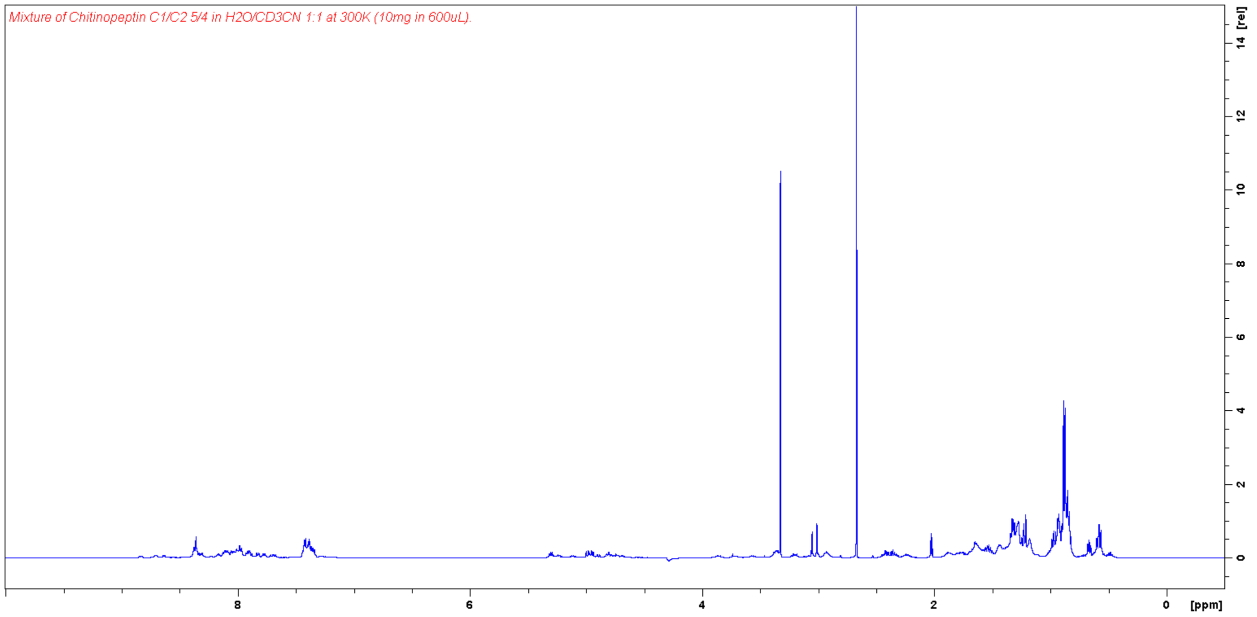

Fig. S18 ^{1}H NMR spectrum (500 MHz, H_2O/CD_3CN 1:1) of chitinopeptin C1 and C2 5/4 mixture.

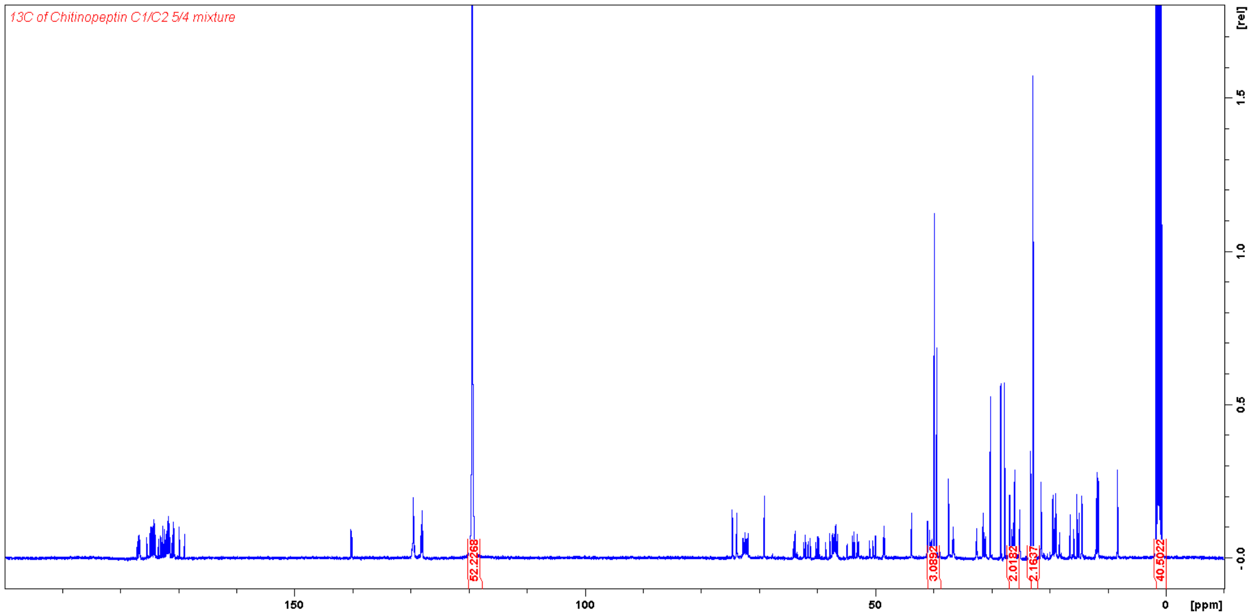

Fig. S19 ^{13}C NMR spectrum (125 MHz, D_2O/CD_3CN 1:1) of chitinopeptin C1 and C2 5/4 mixture.

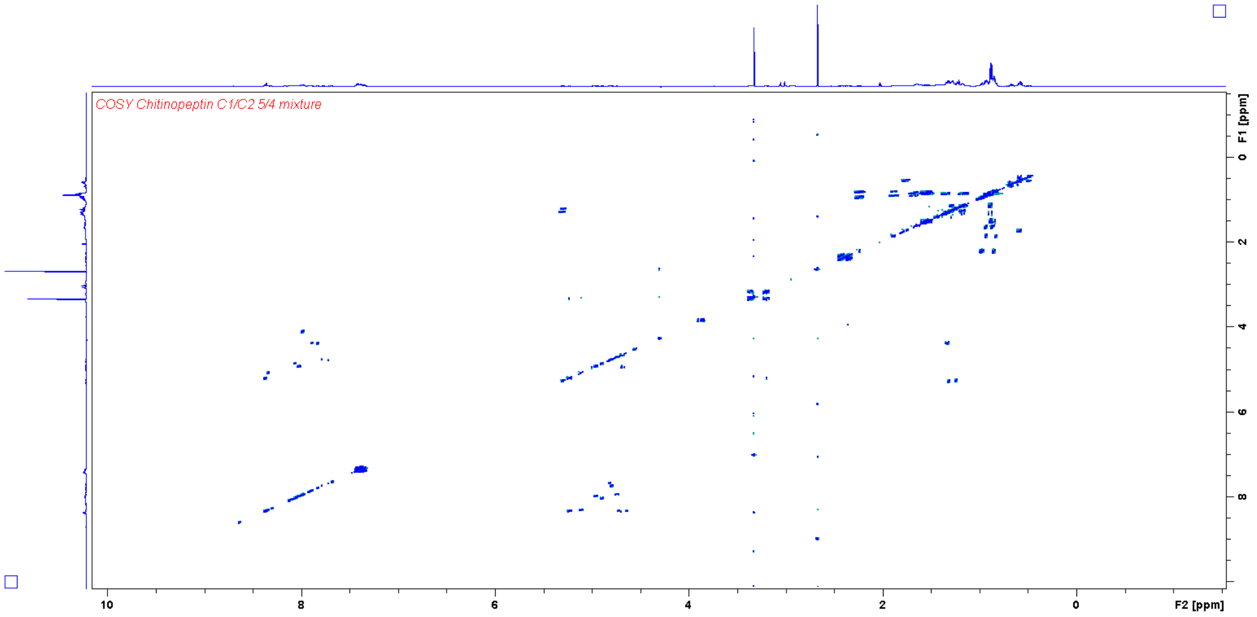

Fig. S20 COSY NMR spectrum (500 MHz, H_2O/CD_3CN 1:1) of chitinopeptin C1 and C2 5/4 mixture.

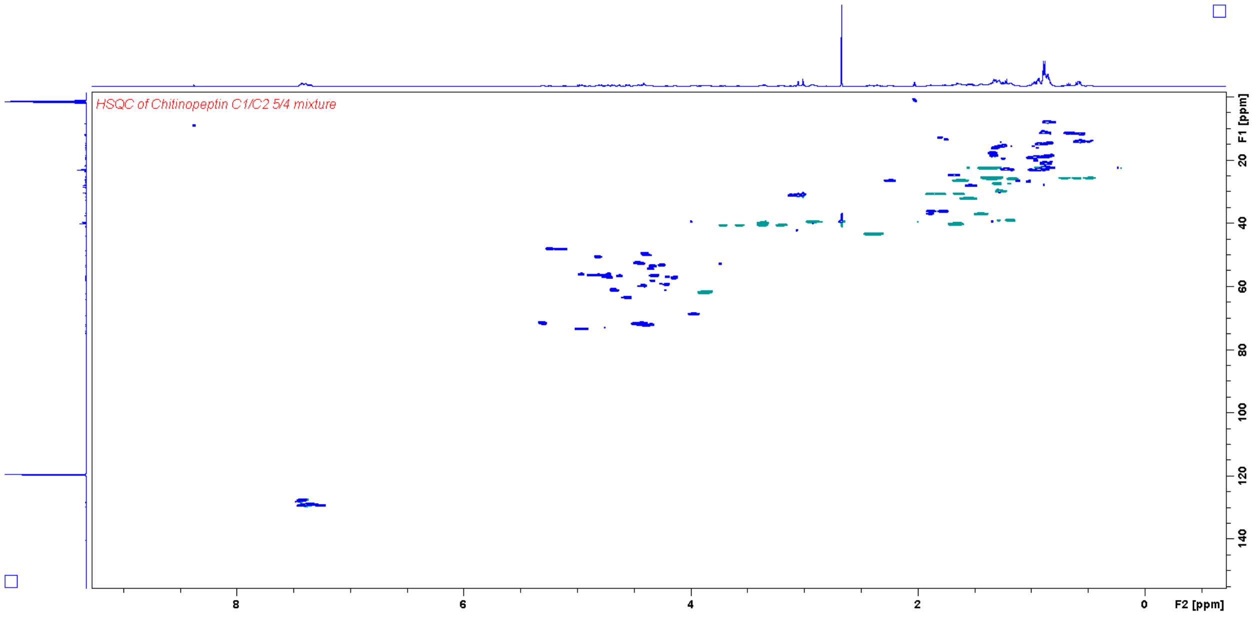

Fig. S21 HSQC NMR spectrum (500 MHz, D_2O/CD_3CN 1:1) of chitinopeptin C1 and C2 5/4.

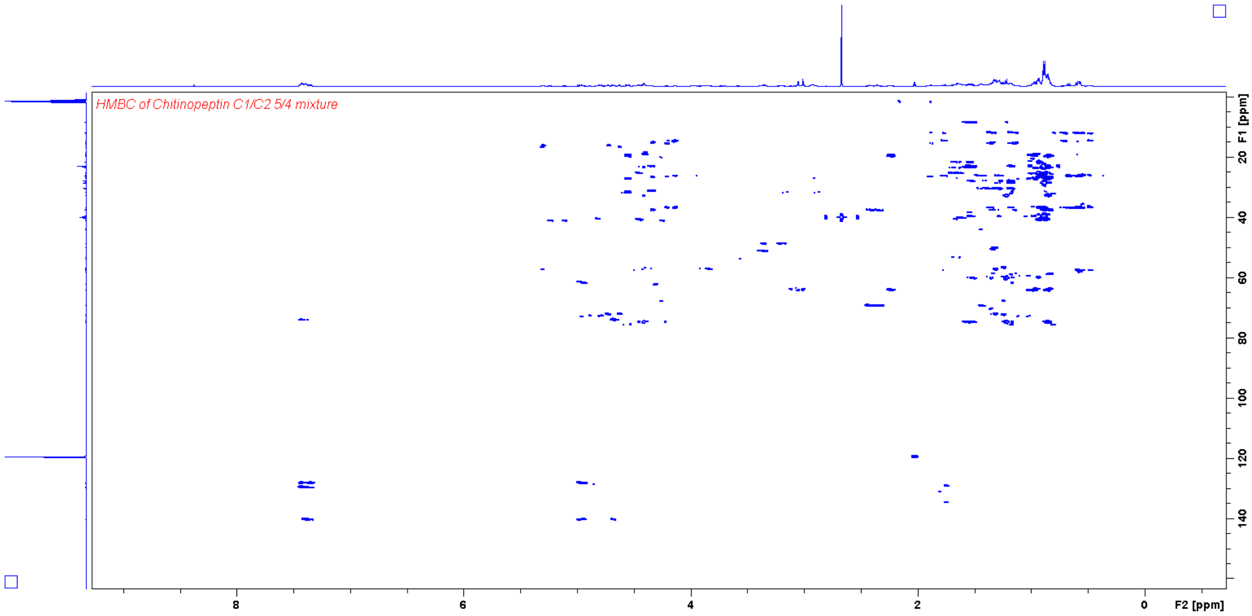

Fig. S22 HMBC NMR spectrum (500 MHz, D_2O/CD_3CN 1:1) of chitinopeptin C1 and C2 5/4.

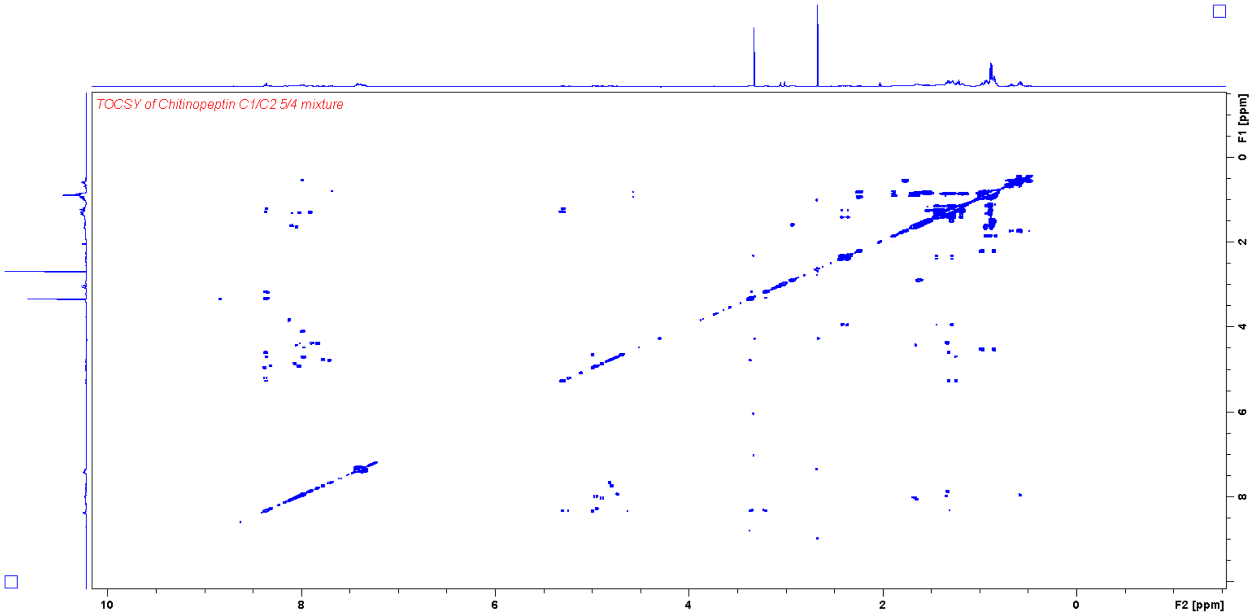

Fig. S23 TOCSY NMR spectrum (500 MHz, H_2O/CD_3CN 1:1) of chitinopeptin C1 and C2 5/4 mixture.

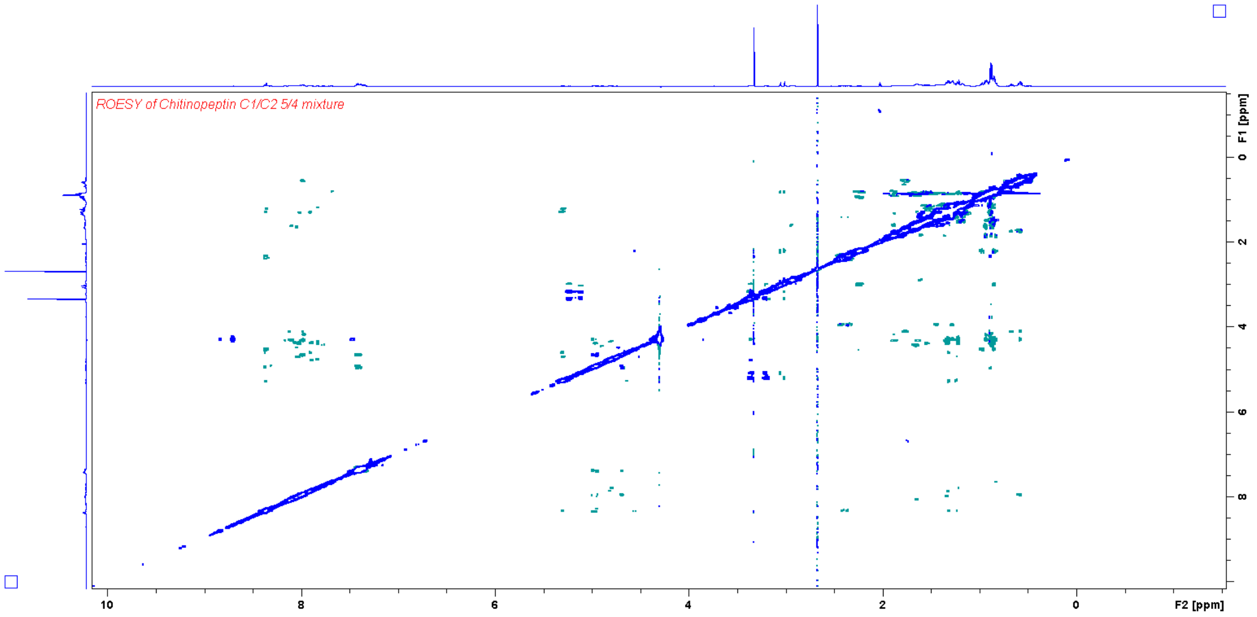

Fig. S24 ROESY NMR spectrum (500 MHz, H_2O/CD_3CN 1:1) of chitinopeptin C1 and C2 5/4 mixture.

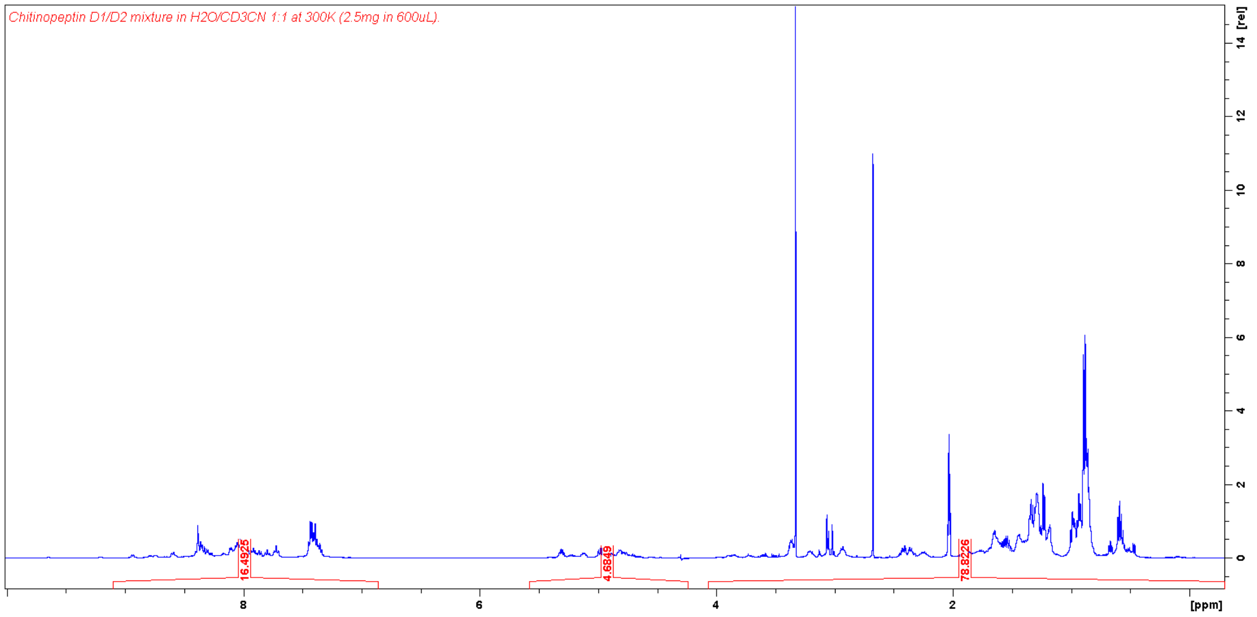

Fig. S25 ^1H NMR spectrum (500 MHz, H_2O/CD_3CN 1:1) of chitinopeptin D1 and D2 mixture.

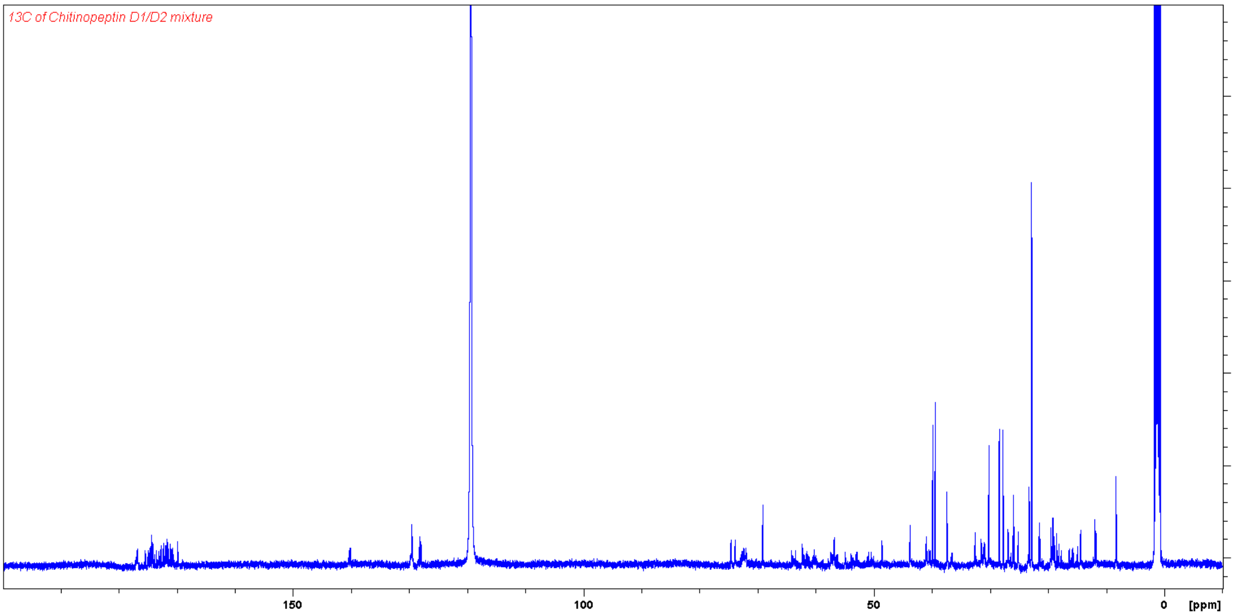

Fig. S26 ^{13}C NMR spectrum (125 MHz, D_2O/CD_3CN 1:1) of chitinopeptin D1 and D2.

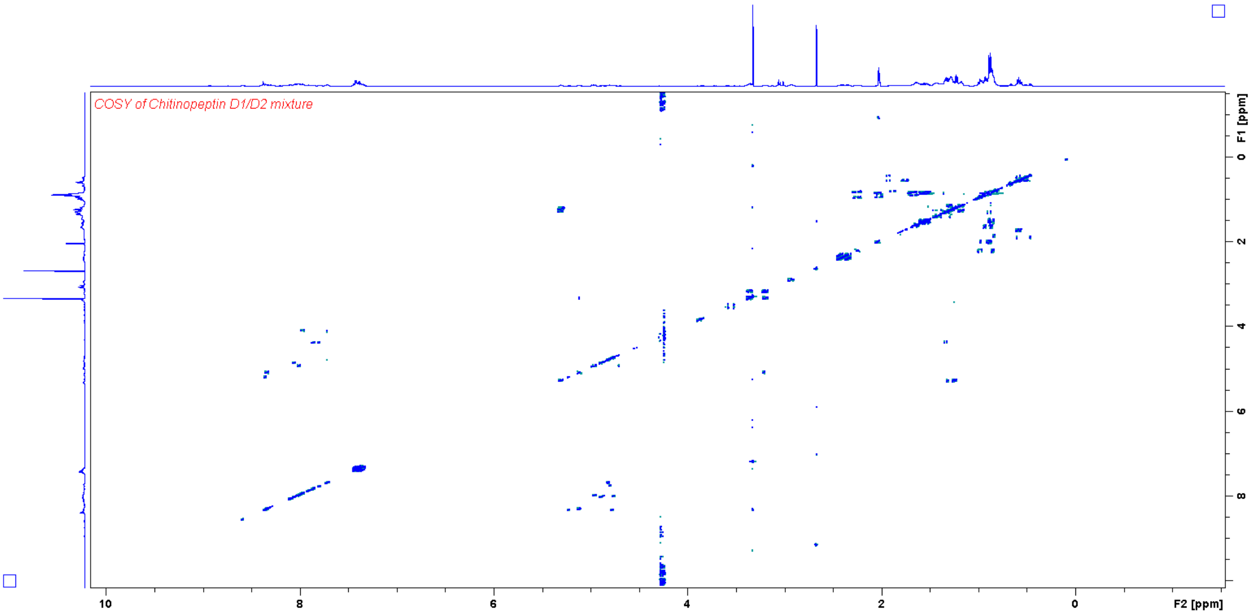

Fig. S27 COSY NMR spectrum (500 MHz, H_2O/CD_3CN 1:1) of chitinopeptin D1 and D2.

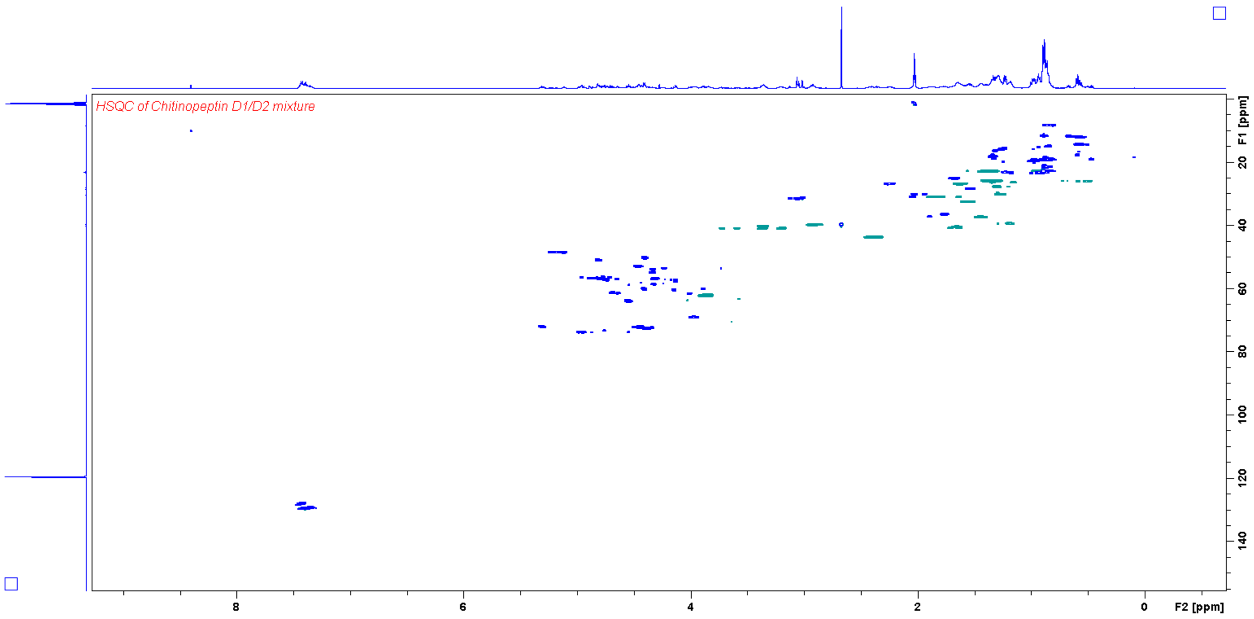

Fig. S28 HSQC NMR spectrum (500 MHz, D_2O/CD_3CN 1:1) of chitinopeptin D1 and D2.

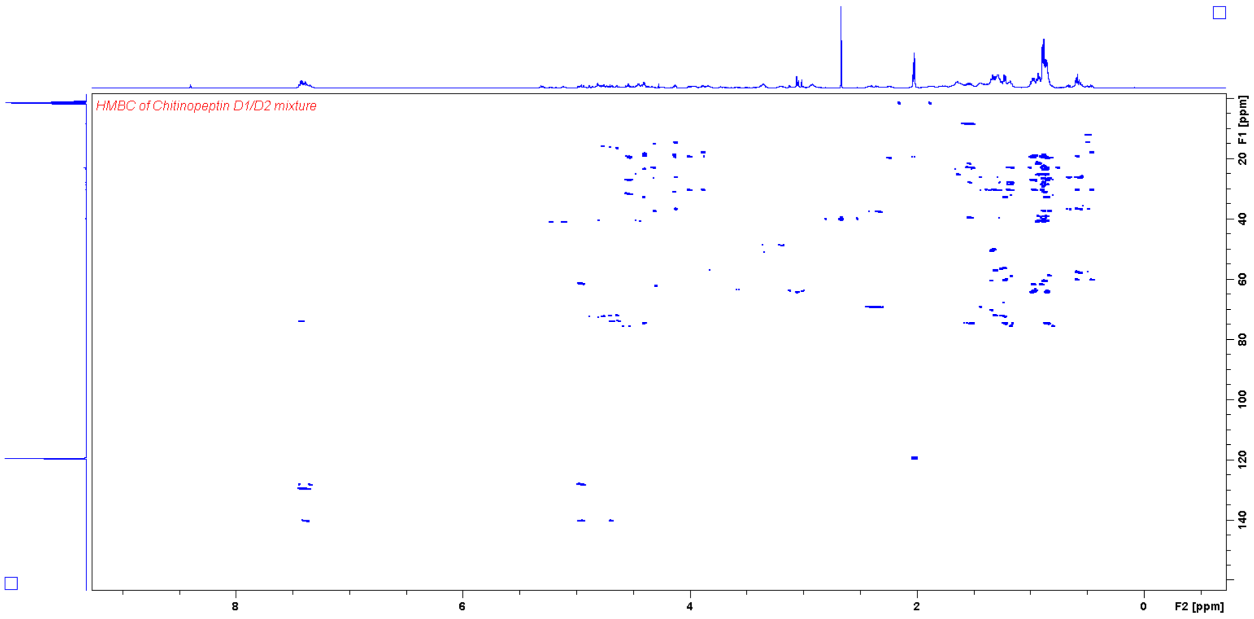

Fig. S29 HMBC NMR spectrum (500 MHz, D_2O/CD_3CN 1:1) of chitinopeptin D1 and D2.

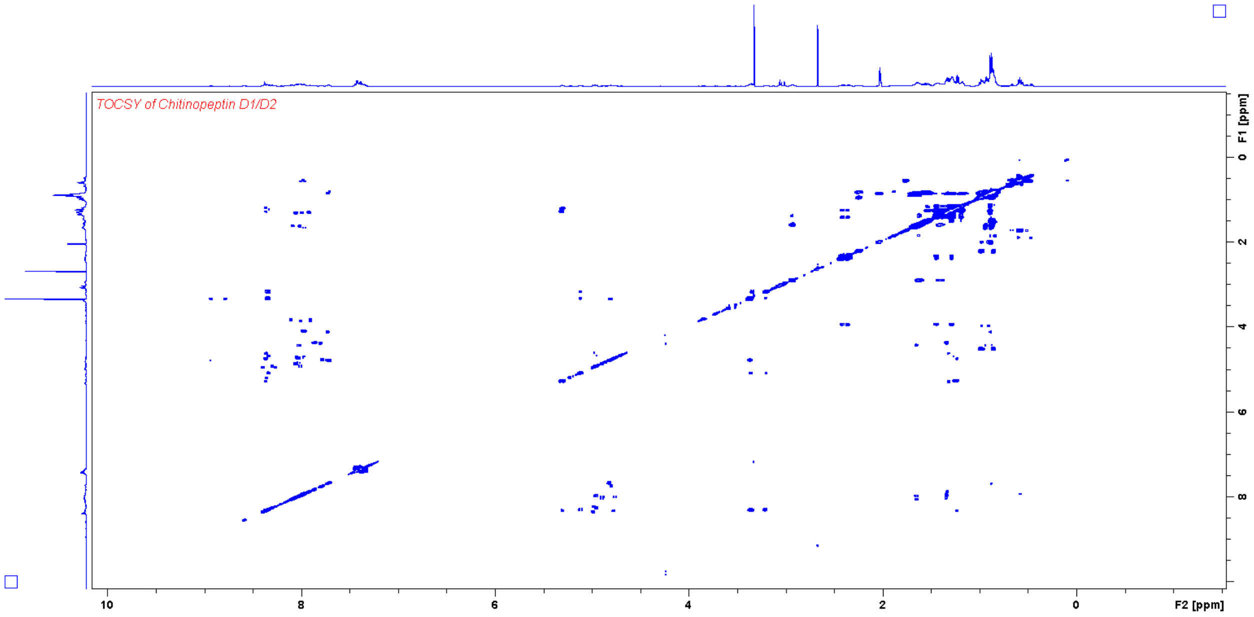

Fig. S30 COSY NMR spectrum (500 MHz, H_2O/CD_3CN 1:1) of chitinopeptin D1 and D2.

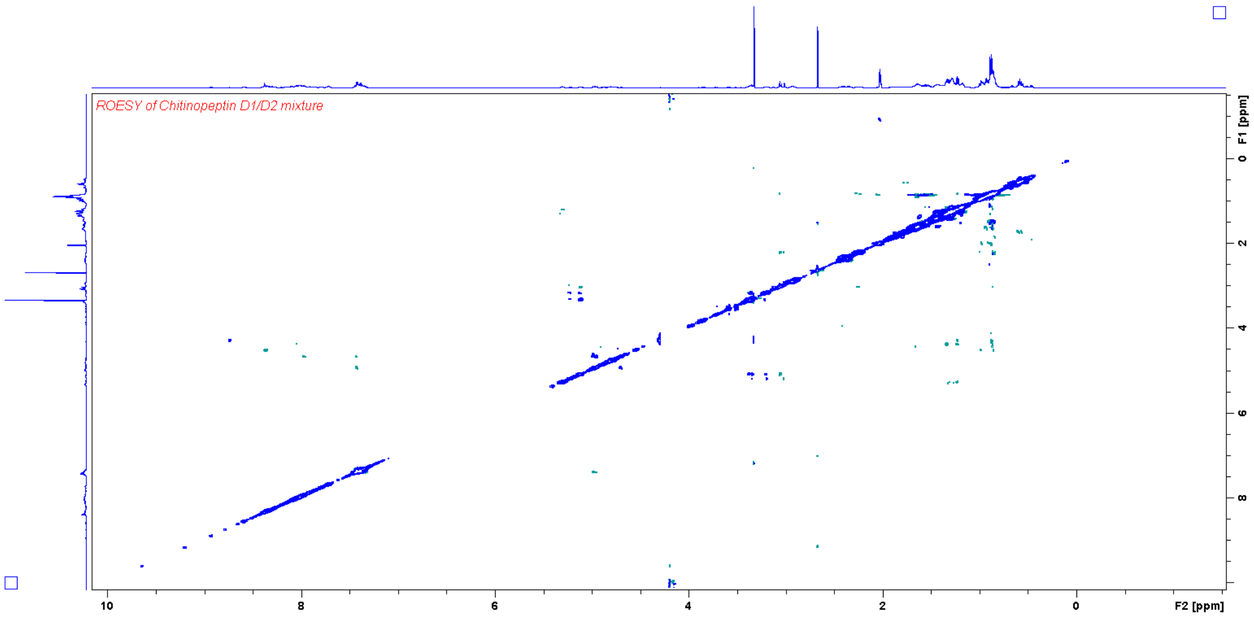

Fig. S31 ROESY NMR spectrum (500 MHz, H_2O/CD_3CN 1:1) of chitinopeptin D1 and D2.

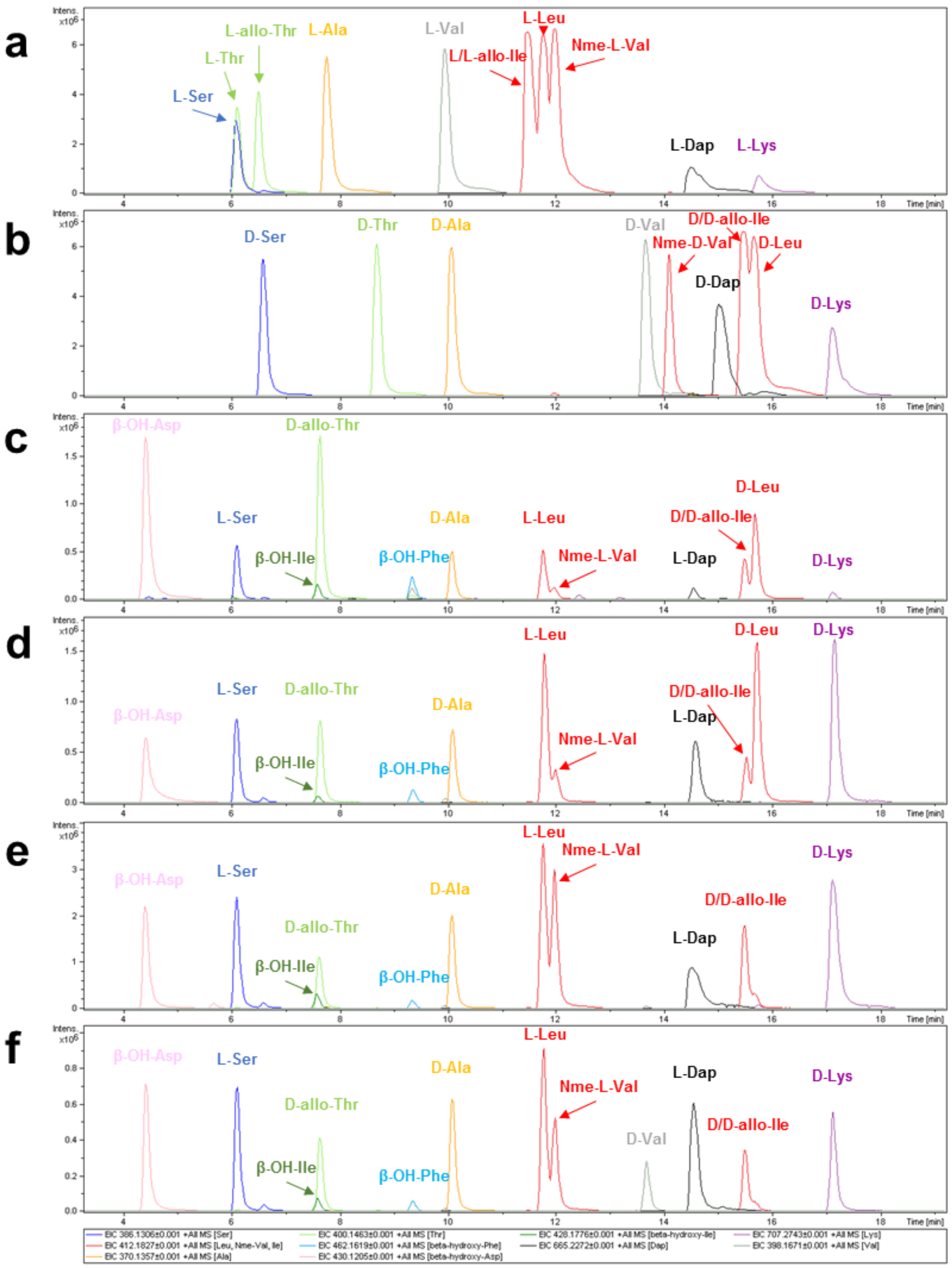

Fig. S32 Comparison of the Marfey derivatization products of the chitinopeptin A-D DCl hydrolysates and commercially available amino acid standards derivatized with L-FDVA. (**A**) Commercially available L-amino acid standards. (**B**) Commercially available D-amino acid standards. (**C**) DCl hydrolysate of chitinopeptin A. (**D**) DCl hydrolysate of chitinopeptin B. (**E**) DCl hydrolysate of chitinopeptin C1+C2. (**F**) DCl hydrolysate of chitinopeptin D1+D2.

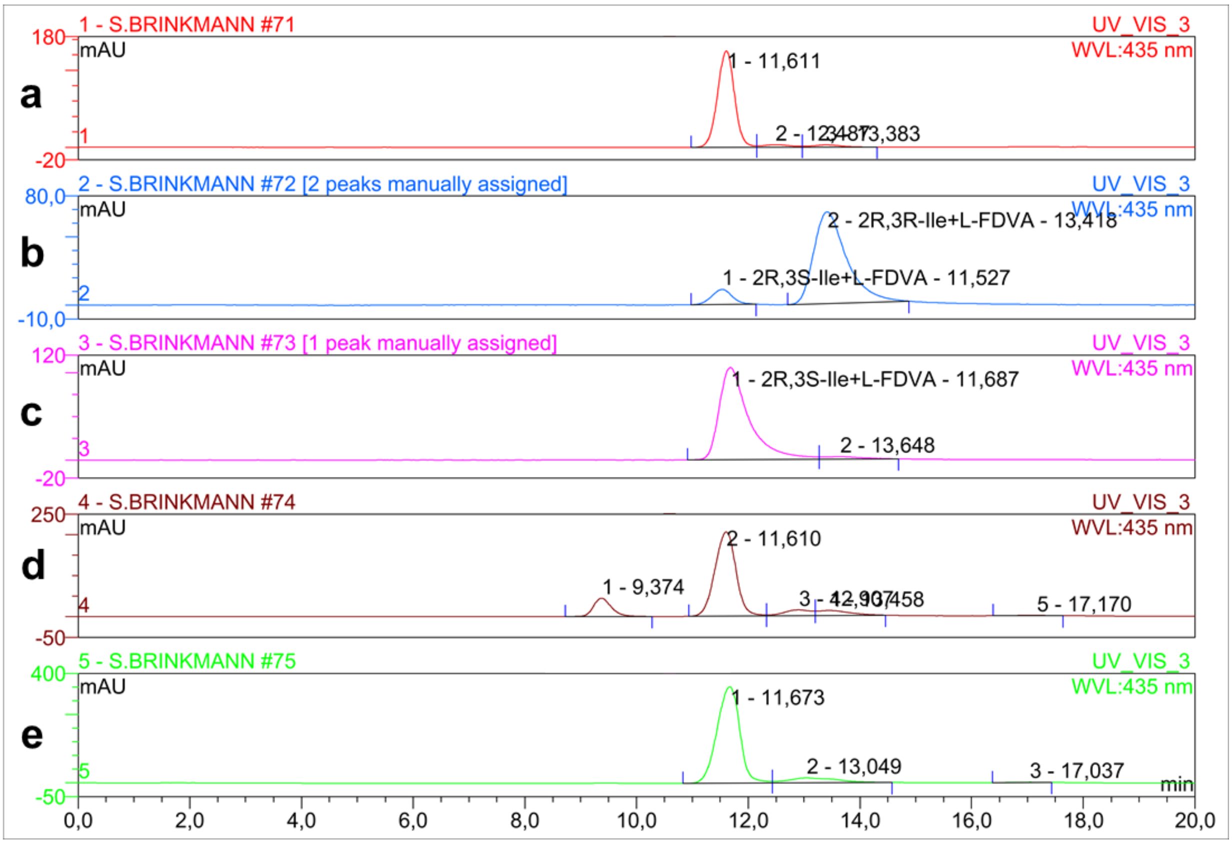

Fig. 33 Chiral HPLC of 2*R*,3*S*-Ile and 2*R*,3*R*-Ile standards and chitinopeptin samples derivatized with L-FDVA. Chiral HPLC conditions: Chiralpak IC; hexane:isopropyl alcohol:formic acid 75:25:0.2. (**A**) chitinopeptin D1+D2, (**B**) 2*R*,3*R*-Ile, (**C**) 2*R*,3*S*-Ile, (**D**) mixture of chitinopeptin A and B, (**E**) chitinopeptin C1+C2

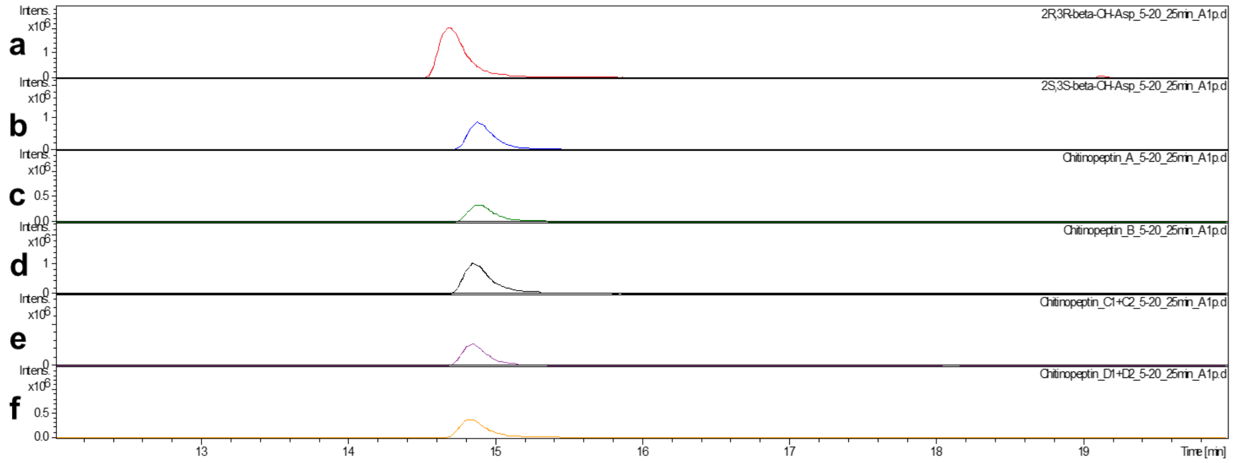

Fig. S34 C18-RP UHPLC-MS extracted ion chromatograms of β-hydroxyaspartic acids derivatized with L-FDVA at *m/z* 430.1205 [M+H]$^+$ within synthesized β-hydroxyaspartic acid standards and chitinopeptin A-D samples. (**A**) (2*R*,3*R*)-3-hydroxyaspartic acid, (**B**) (2*S*,3*S*)-3-hydroxyaspartic acid, (**C**) chitinopeptin A, (**D**) chitinopeptin B, (**E**) chitinopeptin C1+C2, (**F**) chitinopeptin D1+D2

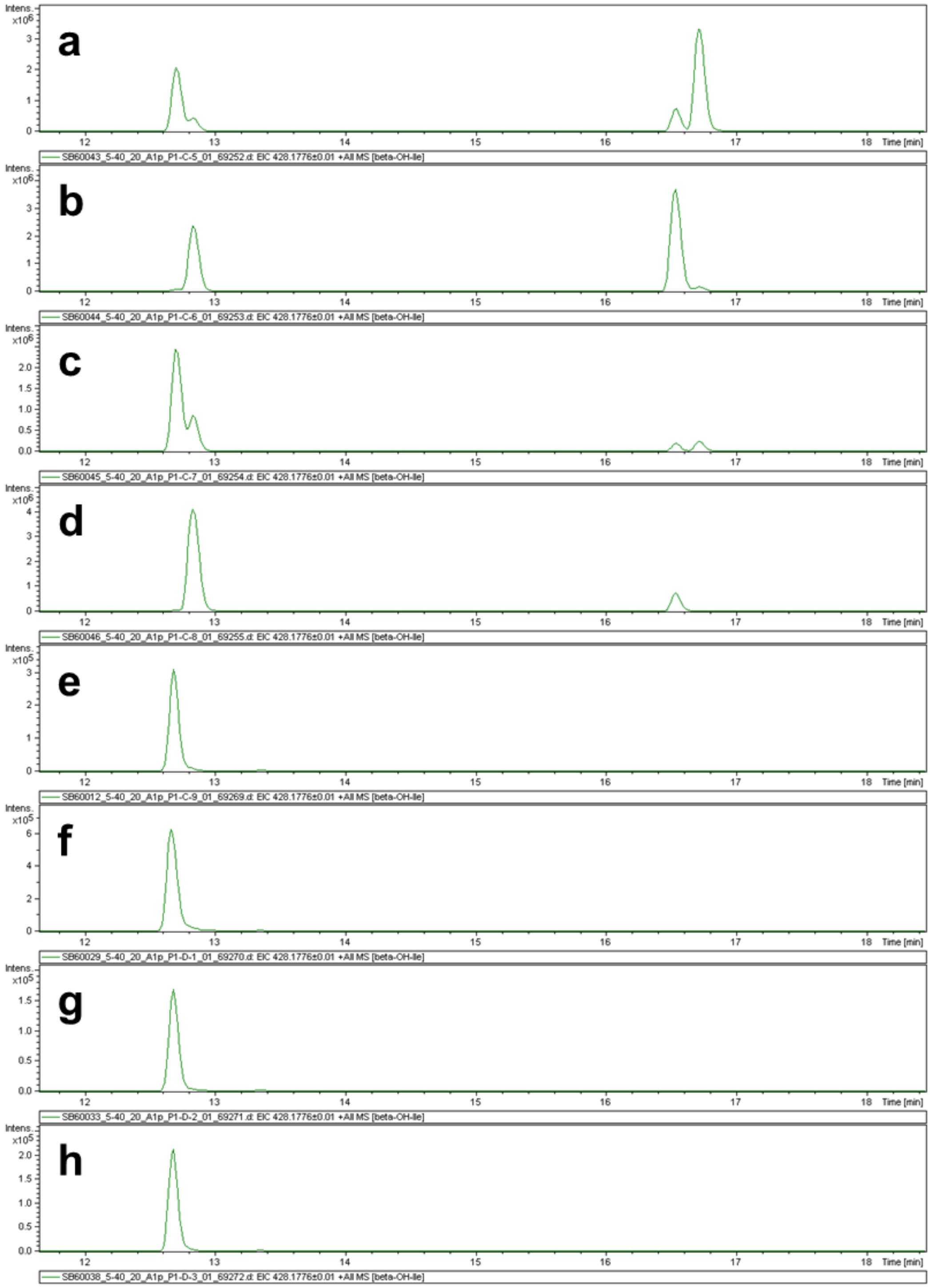

Fig. S35 C18-RP UHPLC-MS extracted ion chromatograms of β-hydroxyisoleucines derivatized with L-FDVA at *m/z* 428.1776 [M+H]$^+$ within synthesized β- hydroxyisoleucine standards and chitinopeptin A-D samples. (**A**) (2*S*,3*R*)- and (2*R*,3*S*)-3-hydroxyisoleucine, (**B**) (2*S*,3*S*)- and (2*R*,3*R*)-3-hydroxyisoleucine, (**C**) (2*S*,3*R*)-3-hydroxyisoleucine, (**D**) (2*S*,3*S*)-3-hydroxyisoleucine, (**E**) chitinopeptin A, (**F**) chitinopeptin B, (**G**) chitinopeptin C1+C2, (**H**) chitinopeptin D1+D2

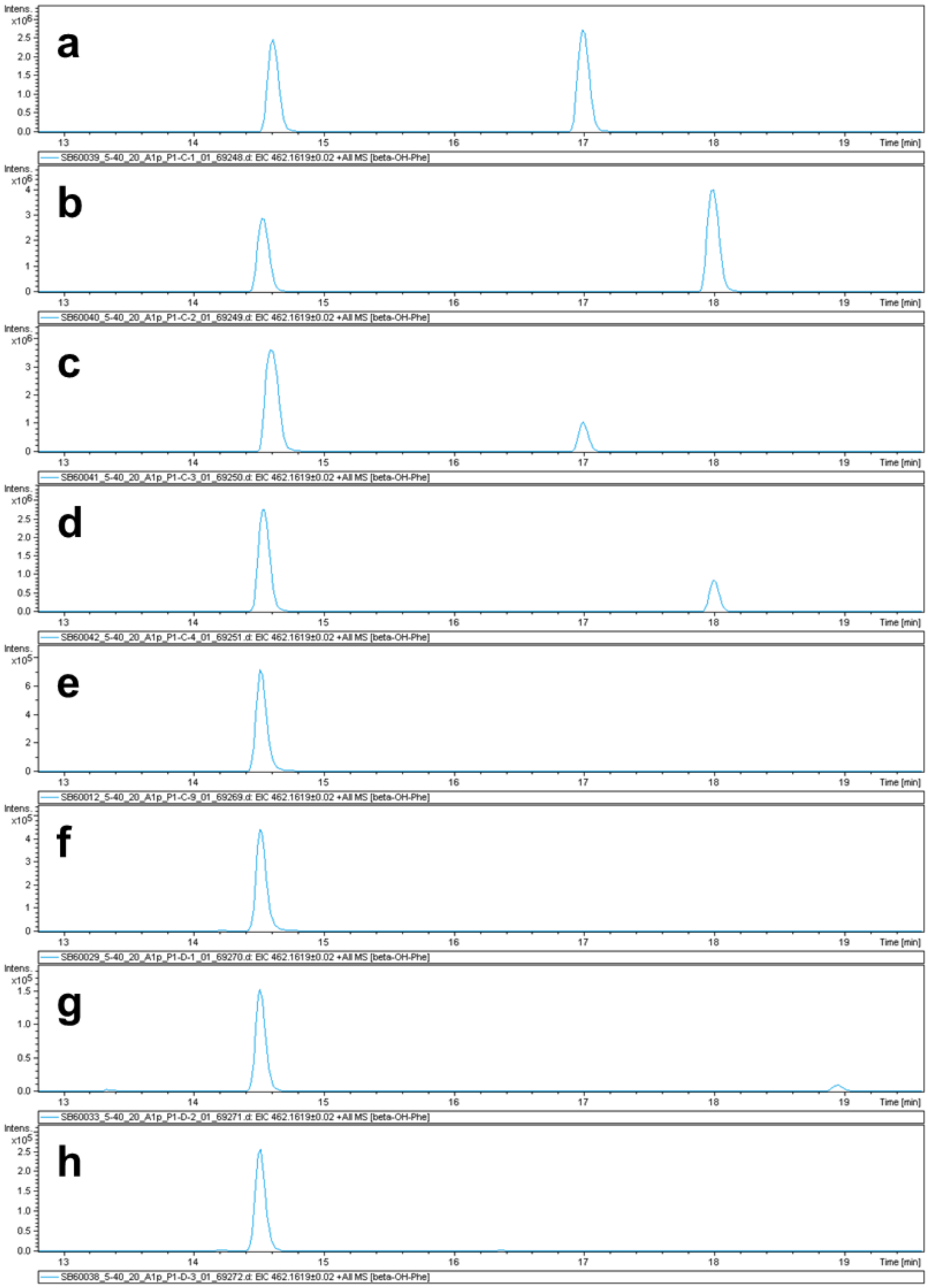

Fig. S36 C18-RP UHPLC-MS extracted ion chromatograms of β hydroxyphenylalanines derivatized with L-FDVA at *m/z* 462.1619 [M+H]$^+$ within synthesized β-hydroxyphenylalanine standards and chitinopeptin A-D samples. (**A**) (2*S*,3*S*)- and (2*R*,3*R*)-3-hydroxyphenylalanine, (**B**) (2*S*,3*R*)- and (2*R*,3*S*)-3-hydroxyphenylalanine, (**C**) (2*S*,3*S*)-3-hydroxyphenylalanine, (**D**) (2*S*,3*R*)-3-hydroxyphenylalanine, (**E**) chitinopeptin A, (**F**) chitinopeptin B, (**G**) chitinopeptin C1+C2, (**H**) chitinopeptin D1+D2

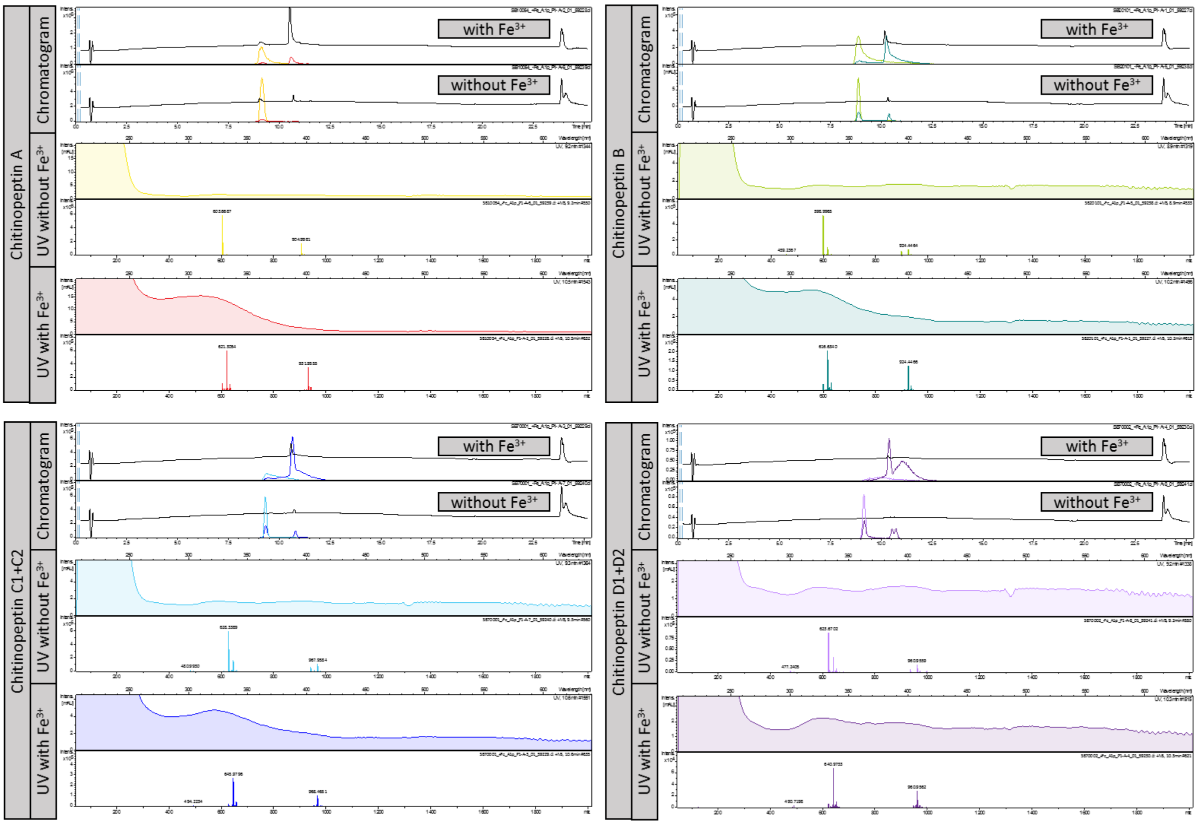

Fig. S37 Iron chelating properties of chitinopeptin A-D. Fe(III)Citrate was added in excess to pure compounds.

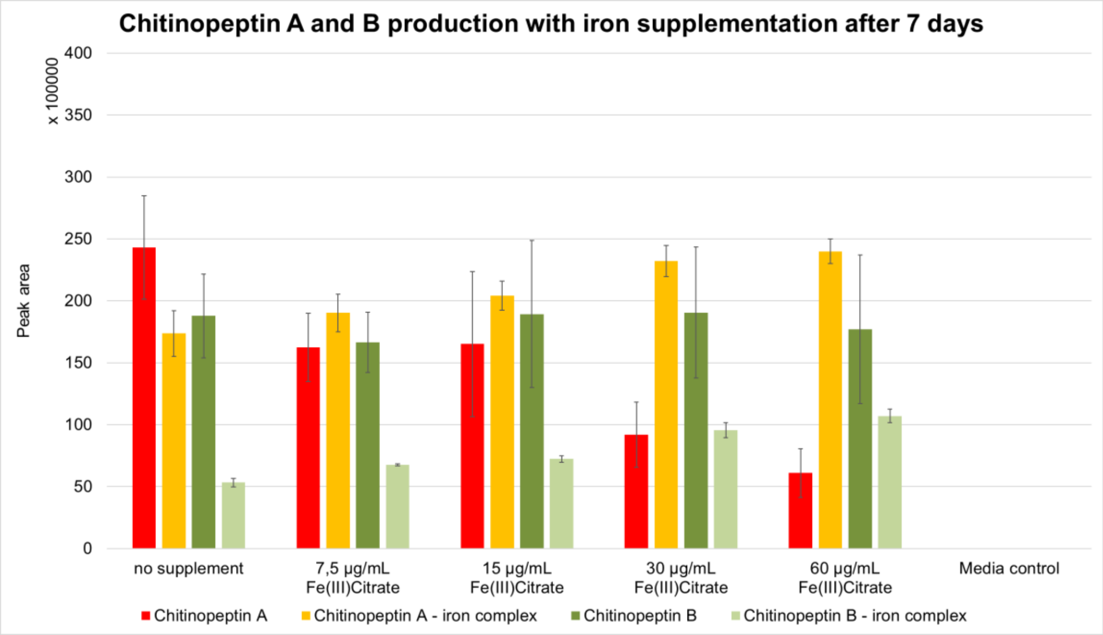

Fig. S38 Production of chitinopeptin A and B in medium 3018 after 7 days of cultivation in 24 well plate cultivation.

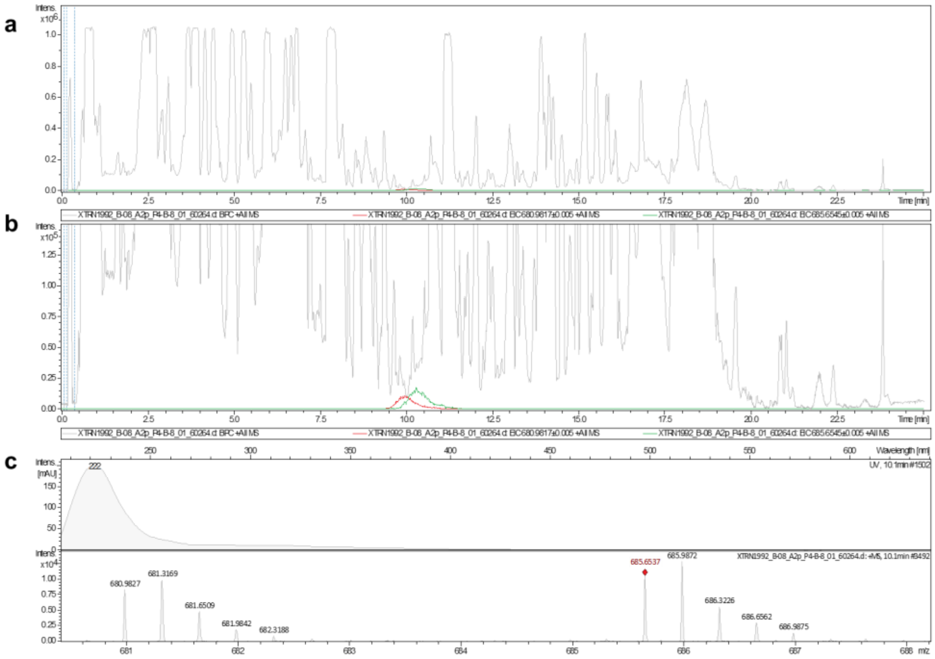

Fig. S 39 Analytics of putatively cyclic lipodepsipeptides produced by *C. niastensis* DSM 24859 with m/z of 680.9817 [M+3H]$^{3+}$ and 685.6545 [M+3H]$^{3+}$. (**A**) overlaid Base Peak Chromatogram (BPC, grey) and Extracted Ion Chromatogram (EIC) of *m/z* 680.9817 [M+3H]$^{3+}$ (red) and 685.6545 [M+3H]$^{3+}$ (green). (**B**) Zoom into the chromatogram. (**C**) Isotope pattern of both derivatives.

3. Supplementary Tables 1 to 7

Tab. S1 Strain list of all *Chitinophaga* strains used for the chemical barcoding matrix and bioactivity-guided NP discovery process.

Phylum	Class	Order	Family	Genus	Species	Strain
Bacteroidetes	Chitinophagia	Chitinophagales	Chitinophagaceae	*Chitinophaga*	*alhagiae*	KCTC62518
Bacteroidetes	Chitinophagia	Chitinophagales	Chitinophagaceae	*Chitinophaga*	*arvensicola*	DSM3695
Bacteroidetes	Chitinophagia	Chitinophagales	Chitinophagaceae	*Chitinophaga*	*barathri*	KCTC42472
Bacteroidetes	Chitinophagia	Chitinophagales	Chitinophagaceae	*Chitinophaga*	*caeni*	KCTC62265
Bacteroidetes	Chitinophagia	Chitinophagales	Chitinophagaceae	*Chitinophaga*	*cymbidii*	KCTC23738
Bacteroidetes	Chitinophagia	Chitinophagales	Chitinophagaceae	*Chitinophaga*	*dinghuensis*	DSM29821
Bacteroidetes	Chitinophagia	Chitinophagales	Chitinophagaceae	*Chitinophaga*	*eiseniae*	DSM22224
Bacteroidetes	Chitinophagia	Chitinophagales	Chitinophagaceae	*Chitinophaga*	*filiformis*	DSM527
Bacteroidetes	Chitinophagia	Chitinophagales	Chitinophagaceae	*Chitinophaga*	*flava*	KCTC62435
Bacteroidetes	Chitinophagia	Chitinophagales	Chitinophagaceae	*Chitinophaga*	*ginsengisegetis*	DSM18108
Bacteroidetes	Chitinophagia	Chitinophagales	Chitinophagaceae	*Chitinophaga*	*ginsengisoli*	DSM18107
Bacteroidetes	Chitinophagia	Chitinophagales	Chitinophagaceae	*Chitinophaga*	*japonensis*	DSM13484
Bacteroidetes	Chitinophagia	Chitinophagales	Chitinophagaceae	*Chitinophaga*	*jiangningensis*	DSM27406
Bacteroidetes	Chitinophagia	Chitinophagales	Chitinophagaceae	*Chitinophaga*	*niabensis*	DSM24787
Bacteroidetes	Chitinophagia	Chitinophagales	Chitinophagaceae	*Chitinophaga*	*niastensis*	DSM24859
Bacteroidetes	Chitinophagia	Chitinophagales	Chitinophagaceae	*Chitinophaga*	*pinensis*	DSM2589
Bacteroidetes	Chitinophagia	Chitinophagales	Chitinophagaceae	*Chitinophaga*	*pinensis*	DSM2588
Bacteroidetes	Chitinophagia	Chitinophagales	Chitinophagaceae	*Chitinophaga*	*rupis*	DSM21039
Bacteroidetes	Chitinophagia	Chitinophagales	Chitinophagaceae	*Chitinophaga*	*sancti*	DSM784
Bacteroidetes	Chitinophagia	Chitinophagales	Chitinophagaceae	*Chitinophaga*	*sedimenti*	KCTC52590
Bacteroidetes	Chitinophagia	Chitinophagales	Chitinophagaceae	*Chitinophaga*	*silvisoli*	KCTC62860
Bacteroidetes	Chitinophagia	Chitinophagales	Chitinophagaceae	*Chitinophaga*	*skermanii*	DSM23857
Bacteroidetes	Chitinophagia	Chitinophagales	Chitinophagaceae	*Chitinophaga*	*sp.*	DSM18078
Bacteroidetes	Chitinophagia	Chitinophagales	Chitinophagaceae	*Chitinophaga*	*terrae*	DSM23920
Bacteroidetes	Chitinophagia	Chitinophagales	Chitinophagaceae	*Chitinophaga*	*varians*	KCTC52926

Tab. S2 | ^{1}H-NMR spectroscopic data of chitinopeptins in a mixture of H_2O and CD_3CN in a ratio of 1:1. Chitinopeptin A: 700.13 MHz, 299 K; chitinopeptins B-D: 500.30 MHz, 300 K; ^{1}H-chemical shifts are referenced to sodium-3-(trimethylsilyl)propionate-2,2,3,3-d_4. The chitinopeptin D sample was a mixture of at least four derivatives with chitinopeptin D1 being the major component. Only the relevant part (Val_{10}-Dab_9) of chitinopeptin D2 was assigned due to the signal overlap.[a]

Pos.	Chitinopeptin A δ_H, mult. [J (Hz)]	Chitinopeptin B δ_H, mult. [J (Hz)]	Chitinopeptin C1 δ_H, mult. [J (Hz)]	Chitinopeptin C2 δ_H, mult. [J (Hz)]	Chitinopeptin D1 δ_H, mult. [J (Hz)]	Chitinopeptin D2 δ_H, mult. [J (Hz)]
**FA**						
1	-	-	-	-	-	
2	3.22, m	3.23, m	2.44/2.35, m	2.42/2.34, m	2.41/2.35, m	
2-Me	1.26, m	1.26, m	-		-	
3	3.27, m	3.26,m	3.98, b	3.96, b	3.96, m	
4	1.61/1.51, m	1.61/1.52, m	1.44, m	1.44, m	1.44, m	
5	1.34/1.27, m	1.33/1.27, m	1.38/1.29, m/b	1.38/1.29, m/b	1.38/1.28, m	
6	1.27/1.21, m	1.29/1.24, m	1.28, b	1.28, b	1.28, m	
7	1.27, m	1.27, m	1.29, b	1.29, b	1.29, m	
8	1.17, m	1.27, m	1.18, b	1.18, b	1.18, b	
9	1.52, m	1.30, m	1.53, m	1.53, b	1.53, b	
10	0.88, m	0.90, m	0.88, d (6.7)	0.88, d (6.7)	0.89, m	
11	0.88, m	-	0.88, (6.7)	0.88, d (6.7)	0.89, m	
**Dab0**						
NH			8.37, d (8.1)	8.345, b	8.34, b	
α			5.24, dd (7.2/15.1)	5.12, dd (7.5/13.8)	5.11, b	
β			3.37/3.20, m	3.36/3.22, m	3.36/3.20, m	
C′			-	-	-	
**NMe-Val1**						
NMe	3.09, s	3.09, s	3.01, s	3.05, s	3.06, s	
α	4.67, d (11.1)	4.67, d (11.3)	4.56, d (10.9)	4.55, d (10.9)	4.55, d (6.1)	
β	2.26, m	2.26, m	2.23, m	2.24, m	2.24, m	
γ	1.02, d (6.7)	1.02, d (6.7)	0.96, b	0.98, d (3.1)	0.97, m	
γ'	0.88, m	0.88, m	0.85, m	0.85, m	0.85, m	
C′	-	-	-	-	-	
**Thr2**						
NH	8.35, d (7.5)	8.35, d (5.4)	8.37, b	8.36, b	8.37, b	
α	4.75, m	4.75, m	4.63, m	4.73, m	4.78, m	
β	5.23, m	5.23, m	5.31, m	5.30, m	5.30, m	
γ	1.17, d (6.5)	1.17, d (6.6)	1.31, d (5.5)	1.24, d (7.5)	1.23, m	
C′	-	-	-	-	-	
**Ala3**						
NH	8.22 (b)	8.22, d (4.6)	7.91, d (7.5)	8.02	8.05, d (5.9)	
α	4.39, m	4.39, m	4.41, b	4.415	4.40, b	
β	1.33, d (7.0)	1.33, d (7.0)	1.33, d (5.1)	1.34	1.34, d (9.4)	
C′	-	-	-	-	-	
**β-OH-Asp4**						
NH	8.13, d (8.9)	8.13, d (9.3)	8.02, b	8.07, b	8.06, b	
α	4.83,m	4.83, m	4.96, m	4.895, m	4.89, b	
β	4.50, m	4.51, b	4.42, d (7.0)	4.48, b	4.46, b	
γ	-	-	-	-	-	
C′	-	-	-	-	-	
**β-OH-Phe5**						
NH	8.34, 3 (6.8)	8.34, 2 (3.4)	8.38, b	8.315, d (5.8)	8.39, b	
α	4.72, m	4.72, m	4.68, m	4.69, m	4.70, m	
β	5.05, d (9.5)	5.05, d (9.4)	4.99, d (8.3)	4.95, d (8.8)	4.98, d (9.0)	
γ	-	-	-	-	-	
δ	7.44, m	7.44, m	7.42, m	7.44, m	7.44, m	
ε	7.37, m	7.37, m	7.39, m	7.39, m	7.39, m	
ζ	7.35,m	7.35, m	7.35, m	7.35, m	7.35, m	
C′	-	-	-	-	-	
**Ile6**						
NH	7.88, d (8.1)	7.88, d (7.8)	7.99, d (7.7)	7.98, d (7.9)	7.97, b	
α	4.12, d (4.5)	4.11, m	4.14, m	4.13, m	4.13, m	
β	1.75, m	1.75, m	1.77, m	1.77, m	1.76, m	
β-Me	0.63, d (7.2)	0.62, d (7.1)	0.57, d (7.5)	0.59, d (7.8)	0.59, b	
γ	0.54/0.49, m/m	0.54/0.49, m/m	0.69/0.48, m/m	0.60/0.48, m/m	0.59/0.50, m/m	
δ	0.56, m	0.56, m	0.67, t (6.4)	0.58, m	0.59, t (7.5)	
C′	-	-	-	-	-	

Tab. S2 (continued)

Pos.	Chitinopeptin A δ_H, mult. [J (Hz)]	Chitinopeptin B δ_H, mult. [J (Hz)]	Chitinopeptin C1 δ_H, mult. [J (Hz)]	Chitinopeptin C2 δ_H, mult. [J (Hz)]	Chitinopeptin D1 δ_H, mult. [J (Hz)]	Chitinopeptin D2 δ_H, mult. [J (Hz)]
**Ser7**						
NH	7.77, d (6.1)	7.77, d (6.1)	8.12, d (6.6)	7.92, d (8.0)	7.90, b	
α	4.28, m	4.29, m	4.32, b	4.32, b	4.32, b	
β	3.93/3.84, d/d (4.4/4.4)	3.92/3.85 (4.2/4.4)	3.87, dd (4.6/12.1)	3.92/3.86, b/b	3.90/3.85, d/b (5.8/-)	
C′	-	-	-	-	-	
**Lys8**						
NH	8.65, d (8.1)	8.65, d (7.9)	8.095, b	8.64, d (7.2)	8.59, d (7.6)	
α	4.39, m	4.38, m	4.34, m	4.36, m	4.33, m	
β	1.93/1.78, m/m	1.93/1.77, m/m	1.85/1.64, m/m	1.90/1.79, m/m	1.88/1.78, m/m	
γ	1.44/1.38, m/m	1.41, m	1.35, m	1.42, m	1.41, m	
δ	1.64, m	1.64, m	1.64, m	1.64, m	1.63, m	
ε	2.95, m	2.95, m	2.93, m	2.94, m	2.93, m	
C′	-	-	-	-	-	
**β-NH_2-Ala9/ β-Dab9/Dab9**						
NH	8.92, d (8.3)	8.90, d (8.5)	8.17, d (5.5)	8.84, d (8.4)	8.93, d (8.3)	3.72/3.59
α	4.79, m	4.79 m	3.72/3.56, m	4.82, m	4.82, m	4.23
β	3.38/3.34, d/d (7.3/5.0)	3.36, m	4.24, m	3.37, m	3.37, m	-
C′	-	-	-	-	-	-
**Leu/Ile/Val10**						
NH	7.94, d (7.3)	7.94, d (6.7)	8.71, d (6.9)	7.68, d (7.6)	7.73, d (7.9)	8.74, d (5.8)
α	4.37, m	4.37, m	4.21, m	4.34, m	4.15, m	4.01, m
β	1.57, m	1.57, m	1.88, m	1.89, m	2.04, (a)	2.03, (a)
γ	1.56, m	1.56, m	1.36/1.17, m/m	1.34/1.15, m/m	0.89, d (1.6)	0.97, b
γ'	-	-	0.93, m	0.83, m	0.88, d (1.6)	0.90, b
δ	0.91, d (6.3)	0.90, d (6.5)	0.89, m	0.89, m		
δ'	0.87, m	0.88, m	-	-		
C′	-	-	-	-		
**β-OH-Asp11**						
NH	7.97, d (8.8)	7.98, d (8.5)	7.97, d (8.0)	7.98, d (8.0)	7.97, b	
α	4.70, m	4.70, m	4.74, b	4.75, b	4.75, d (2.8)	
β	4.41, b	4.43, b	4.52, b	4.45, b	4.46, b	
γ	-	-	-	-	-	
C′	-	-	-	-	-	
**Leu12**						
NH	8.14, b	8.15, d (7.9)	8.05, d (8.0)	8.10, d (7.6)	8.02, b	
α	4.42, b	4.42, b	4.45, b	4.466, b	4.47, b	
β	1.64, m	1.65, m	1.67, m	1.65, m	1.65, m	
γ	1.64, m	1.65, m	1.67, m	1.65, m	1.67, m	
δ	0.93, d (6.3)	0.93, d (6.3)	0.94, b	0.927, d (2.1)	0.93, b	
δ'	0.86, m	0.86, m	0.88, b	0.86, b	0.87, b	
C′	-	-	-	-	-	
**β-OH-Asp13**						
NH	7.68, d (5.9)	7.69, d (8.9)	7.78, d (8.6)	7.71, b	7.71, d (9.3)	
α	4.84, m	4.85, m	4.79, m	4.82, m	4.82, m	
β	4.33, b	4.35, m	4.36, m	4.38, m	4.36, m	
γ	-	-	-	-	-	
C′	-	-	-	-	-	
**β-OH-Ile14**						
NH	7.88, d (8.3)	7.88, d (7.9)	7.83, d (8.5)	7.89, d (9.3)	7.86, d (8.6)	
α	4.40, b	4.41, b	4.42, b	4.41, b	4.40, b	
β	-	-	-	-	-	
β-Me	1.25, s	1.25, s	1.21, s	1.23, s	1.23, s	
γ	1.58/1.53, m	1.58/1.54, m	1.55, m	1.57, m	1.56, m	
δ	0.85, m	0.86, m	0.85, m	0.86, m	0.86, m	
C′	-	-	-	-	-	

[a]Abbreviations: b = broad signal; a = ^{1}H-signal below solvent signal; n.a. = not assigned

Tab. S3 ^{13}C-NMR spectroscopic data of chitinopeptins A-D in a mixture of D_2O and CD_3CN in a ratio of 1:1. Chitinopeptin A: 176.05 MHz, 299 K; chitinopeptins B-D: 125.82 MHz, 300 K. ^{13}C-chemical shifts were referenced to the solvent signal (CD_3CN, ^{13}C: 1.30 ppm). The chitinopeptin D sample was a mixture of at least four derivatives with chitinopeptin D1 being the major component. Only the relevant part (Val_{10}-Dab_9) of chitinopeptin D2 was assigned due to the signal overlap.[a]

Pos.	Chitinopeptin A δ_C, mult.	Chitinopeptin B δ_C, mult.	Chitinopeptin C1 δ_C, mult.	Chitinopeptin C2 δ_C, mult.	Chitinopeptin D1 δ_C, mult.	Chitinopeptin D2 δ_C, mult.
**FA**						
1	177.81, C	177.79, C	174.27,C	174.35, C	n.a.	
2	36.08, CH	36.05, CH	43.85, CH_2	43.80, CH_2	43.80, CH_2	
2-Me	14.77, CH_3	14.76, CH_3	-	-	-	
3	55.85, CH	55.60, CH	69.19, CH	69.18, CH	69.19	
4	32.37, CH_2	~32.2 (b), CH_2	37.49, CH_2	37.49, CH_2	37.48, CH	
5	26.29, CH_2	26.25, CH_2	26.02, CH_2	25.99, CH_2	25.99, CH_2	
6	30.11, CH_2	29.79, CH_2	30.28, CH_2	30.28, CH_2	30.28, CH_2	
7	27.56, CH_2	29.46, CH_2	27.83, CH_2	27.83, CH_2	27.83, CH_2	
8	39.40, CH_2	32.24, CH_2	39.52, CH_2	39.52, CH_2	39.52, CH_2	
9	28.37, CH	23.09, CH_2	28.47, CH	28.47, CH	28.47, CH_2	
10	22.84, CH_3	14.31, CH_3	22.88, CH_3	22.88, CH_3	22.88, CH_3	
11	22.81, CH_3	-	22.88, CH_3	22.88, CH_3	22.88, CH_3	
**Dab0**						
NH	-		-	-	-	
α	-		48.52, CH	48.67, CH	48.68, CH	
β	-		41.02, CH_2	41.07, CH_2	41.04, CH_2	
C′	-		171.75, C	171.76, C	171.76, C	
**N-Me-Val1**						
NMe	31.64, CH_3	31.65, CH_3	31.44, CH_3	31.60, CH_3	31.65, CH_3	
α	63.22, CH	63.21, CH	63.88, CH	64.14, CH	64.17, CH	
β	26.70, CH	26.71, CH	26.98, CH	26.90, CH	26.93, CH	
γ	19.74, CH_3	19.73, CH_3	19.51, CH_3	19.60, CH_3	19.53, CH_3	
γ'	19.30, CH_3	19.28, CH_3	19.06, CH_3	19.23, CH_3	19.09, CH_3	
C′	172.16, C	172.11, C	171.90, C	172.17, C	171.76, C	
**Thr2**						
NH	-	-	-	-	-	
α	~56.4 (b), CH	~56.3 (b), CH	57.14, CH	56.56, CH	56.34, CH	
β	73.06, CH	73.03, CH	71.96, CH	72.21, CH	72.10, CH	
γ	15.40, CH_3	15.44, CH_3	16.54, CH_3	15.90, CH_3	15.75, CH_3	
C′	169.65, C	169.57, C	169.91, C	169.88, C	169.90, C	
**Ala3**						
NH	-	-	-	-	-	
α	51.12, CH	50.98, CH	50.03, CH	50.48, CH	50.55, CH	
β	17.90, CH_3	17.86, CH_3	18.93, CH_3	18.34, CH_3	18.20, CH_3	
C′	175.19, C	175.10, C	174.50, C	174.97, C	175.00, C	
**β-OH-Asp4**						
NH	-	-	-	-	-	
α	57.36, CH	57.24, CH	56.59, CH	56.86, CH	56.84, CH	
β	72.56, CH	72.45, CH	72.85, CH	72.00, CH	72.44, CH	
γ	176.69, C	176.7 (b), C	177.11, C	176.76, C	n.a.	
C′	173.27, C	173.20, C	172.47, C	172.95, C	n.a.	
**β-OH-Phe5**						
NH	-	-	-	-	-	
α	61.62, CH	61.50, CH	61.26, CH	61.64, CH	61.53, CH	
β	73.78, CH	73.66, CH	73.91, CH	73.91, CH	73.95, CH	
γ	140.39, C	140.34, CH	140.32, C	140.17, C	140.17, C	
δ	128.23,CH	128.25, CH	128.05, CH	128.26, CH	128.25, C	
ε	129.49, CH	129.52, CH	129.58, CH	129.64, CH	~129.6 (a), CH	
ζ	129.38, CH	129.42, CH	129.42, CH	129.53, CH	~129.5 (a), CH	
C′	171.29, C	171.24, CH	171.58, C	171.65, C	171.57, C	
**Ile6**						
NH	-	-	-	-	-	
α	57.48, CH	57.39, CH	57.89, CH	57.47, CH	57.49, CH	
β	36.57, CH	36.55, CH	36.70, CH	36.50, CH	36.55, CH	
β-Me	14.46, CH_3	14.45, CH_3	14.58, CH_3	14.42, CH_3	14.45, CH_3	
γ	26.21, CH_2	26.22, CH_2	26.12, CH_2	26.12, CH_2	26.13, CH_2	
δ	12.16, CH_3	12.15, CH_3	11.86, CH_3	12.05, CH_3	12.04, CH_3	
C′	174.31, C	174.25, C	174.17, C	174.12, C	174.11, C	

Tab. S3 (continued)

Position	Chitinopeptin A δ_C, mult.	Chitinopeptin B δ_C, mult.	Chitinopeptin C1 δ_C, mult.	Chitinopeptin C2 δ_C, mult.	Chitinopeptin D1 δ_C, mult.	Chitinopeptin D2 δ_C, mult.
**Ser7**						
NH	-	-	-	-	-	
α	56.71, CH	56.60, CH	57.02, CH	56.98, CH	56.98, CH	
β	62.55, CH_2	62.40, CH_2	62.09, CH_2	62.40, CH_2	62.36, CH_2	
C′	173.16, C	173.10, C	172.77, C	173.09, C	173.09, C	
**Lys8**						
NH	-	-	-	-	-	
α	54.75, CH	54.64, CH	53.98, CH	54.93, CH	55.03	
β	31.26, CH_2	31.20, CH_2	31.07, CH_2	31.18, CH_2	31.16, CH_2	
γ	22.97, CH_2	22.97, CH_2	22.92, CH_2	22.95, CH_2	22.97, CH_2	
δ	27.06, CH_2	26.91, CH_2	26.88, CH_2	26.98, CH_2	26.98, CH_2	
ε	40.31, CH_2	40.02, CH_2	39.91, CH_2	40.04, CH_2	39.93, CH_2	
C′	175.32, C	175.25, C	174.85, C	175.46, C	175.48, C	
**β-NH_2-Ala9/ β-Dab9/Dab9**						
NH	-	-	-	-	-	-
α	51.28, CH	51.03, CH	41.07, CH	51.05, CH	50.99, CH	41.04,
β	40.76, CH_2	40.48, CH_2	53.70, CH_2	40.34, CH_2	40.31, CH_2	53.67
C′	170.76, C	170.67, C	168.96, C	170.78, C	170.71, C	~169.0 (a)
**Leu/Ile/Val10**						
NH	-	-	-	-	-	-
α	53.64, CH	53.54, CH	59.84, CH	58.62, CH	60.52, CH	~61.7 (a)
β	40.57, CH_2	40.52, CH_2	36.61, CH	37.43, CH	31.00, CH	~ 30.3(a)
γ	25.18, CH	25.20, CH	15.33, CH_2	14.97, CH_2	19.30, CH_2	~19.3 (a)
γ'	-	-	26.34, CH_3	26.53, CH_3	18.60, CH_3	~19.3 (a)
δ	23.12, CH_3	23.12, CH_3	11.68, CH_3	11.79, CH_3	-	-
δ'	21.82, CH_3	21.80, CH_3	-	-	-	-
C′	174.57, C	174.49, C	174.02, C	173.47, C	173.55, C	174.17
**β-OH-Asp11**						
NH	-	-	-	-	-	
α	57.18, CH	57.05, CH	57.42, CH	57.20, CH	57.37, CH	
β	72.06, CH	71.94, CH	72.29, CH	72.48, CH	72.10, CH	
γ	176.80, C	~176.8 (b), C	176.68, C	176.76, C	n.a.	
C′	172.33, C	172.25, C	172.05, C	172.29, C	n.a.	
**Leu12**						
NH	-	-	-	-	-	
α	53.23, CH	53.11, CH	53.14, CH	52.91, CH	~53.0 (a), CH	
β	40.37, CH_2	40.33, CH_2	40.71, CH_2	40.46, CH_2	40.57, CH_2	
γ	25.18, CH	25.18, CH	25.23, CH	25.14, CH	25.15, CH	
δ	23.34, CH_3	23.34, CH_3	23.37, CH_3	23.37, CH_3	23.36, CH_3	
δ'	21.52, CH_3	21.51, CH_3	21.58, CH_3	21.49, CH_3	21.53, CH_3	
C′	174.55, C	174.49, C	174.66, C	174.58, C	n.a.	
**β-OH-Asp13**						
NH	-	-	-	-	-	
α	56.71, CH	56.60, CH	56.86, CH	56.81, CH	~56.8 (a), CH	
β	72.88, CH	72.77, CH	72.51, CH	72.68, CH	~72.7 (a), CH	
γ	176.82, C	~176.8 (b), C	176.88, C	176.88, C	n.a.	
C′	171.94, C	171.87, C	171.84, C	171.98, C	n.a.	
**β-OH-Ile14**						
NH	-	-	-	-	-	
α	60.68, CH	60.59, CH	60.04, CH	60.29, CH	60.35, CH	
β	74.68, C	74.61, C	74.72, C	74.63, C	74.58, C	
β-Me	23.34, CH_3	23.28, CH_3	23.25, CH_3	23.28, CH_3	23.26, CH_3	
γ	32.69, CH_2	32.65, CH_2	32.61, CH_2	32.64, CH_2	32.64, CH_2	
δ	8.41, CH_3	8.39, CH_3	8.35, CH_3	8.35, CH_3	8.35, CH_3	

[a]Abbreviations: b = broad signal; a = ^{13}C-signal below solvent signal; n.a. = not assigned

Tab. S4 Bioactivity screening data of chitinopeptins A-D.*

MIC (µg/mL)		Compounds	Chitinopeptin								Reference data		
		Derivatives	A		B		C1+C2		D1+D2		Rifamicin	Tetracycline	Gentamycin/ Isoniazid[a]
		Iron	-	+	-	+	-	+	-	+	Tebuconazole[b]	Amphotericin B[c]	Nystatin[d]
Organism and genotype	Gram -	*E. coli* ATCC 35218	>64	n.d.	>64	n.d.	>64	n.d.	>64	n.d.	4	4	0.125
		E. coli ATCC 25922 ΔTolC	>64	n.d.	>64	n.d.	>64	n.d.	>64	n.d.	2	0.5	0.25
		E. coli MG1655	>256	n.d.	n.d.	n.d.	n.d.	n.d.	n.d.	n.d.	16	0.5	0.5
		P. aeruginosa ATCC 27853	>64	n.d.	>64	n.d.	>64	n.d.	>64	n.d.	32	64	1
		P. aeruginosa PAO750	>64	n.d.	n.d.	n.d.	n.d.	n.d.	n.d.	n.d.	16	0.25	0.125
		K. pneumoniae ATCC 13883	>64	n.d.	n.d.	n.d.	n.d.	n.d.	n.d.	n.d.	8	2	≤0.031
		M. catarrhalis ATCC 25238	2	4	2	n.d.	2	n.d.	2	n.d.	≤0.031	1	0.125
		A. baumannii ATCC 19606	16	n.d.	64	n.d.	16	n.d.	16	n.d.	2	16	16
	Gram +	*B. subtilis* DSM 10	4	8	4	n.d.	4	n.d.	4	n.d.	≤0.031	4	≤0.031
		S. aureus ATCC 25923	>64	n.d.	>64	n.d.	>64	n.d.	>64	n.d.	≤0.031	1	0.063
		M. luteus DSM 20030	32	n.d.	32	n.d.	32	n.d.	32	n.d.	≤0.031	0.5	≤0.031
		L. monocytogenes DSM 20600	>64	n.d.	n.d.	n.d.	n.d.	n.d.	n.d.	n.d.	≤0.031	0.5	≤0.031
		M. smegmatis ATCC 607	>64	n.d.	>64	n.d.	>64	n.d.	>64	n.d.	8	0.5	1[a]
	Yeast/Fungi	*C. albicans* FH2173	4	32	8	n.d.	16	n.d.	16	n.d.	2[b]	4[c]	16[d]
		A. flavus ATCC 9170	>64	n.d.	>64	n.d.	64	n.d.	64	n.d.	2[b]	16-8[c]	16d
		Z. tritici MUCL45407	16	32-16	16	n.d.	16	n.d.	16-8	n.d.	0.5[b]	0.031[c]	0.5[d]
		F. oxysporum ATCC 7601	>64	n.d.	>64	n.d.	>64	n.d.	>64	n.d.	>64[b]	4[c]	4[d]

*Abbreviation: n.d. = not determined

Tab. S5 Partial Clustal W alignment (111) of the proposed dinuclear binding motif (His-X-His-X-Asp-His) by Makris *et al.* (2010) (79) in all three diiron-monooxygenases with other homologs in NRPS antibiotic biosynthetic pathways.

Species	Antibiotic	Protein-ID	Consensus sequence						
Chitinophaga eiseniae	Chitinopeptin A-B	SKA37886.1	H^{303}-N-H-Q-D-H	X_{66}	E	X_{25}	D	X_{26}	E
Chitinophaga flava	Chitinopeptin C-D	RBL90130.1	H^{303}-N-H-Q-D-H	X_{66}	E	X_{25}	D	X_{26}	E
Chitinophaga oryziterrae	/	MVT41610.1	H^{301}-N-H-Q-D-H	X_{66}	E	X_{25}	D	X_{26}	E
Chitinophaga nistansis	/	PSL45608.1	H^{303}-N-H-Q-D-H	X_{66}	E	X_{25}	D	X_{26}	E
Streptomyces venezuelae	Chloramphenicol	CCA54208.1	H^{305}-N-H-Q-D-H	X_{66}	E	X_{25}	D	X_{26}	E
Actinoplanes teichomycetius	Teicoplanin	CAE53366.1	H^{301}-G-H-S-D-H	X_{65}	E	X_{25}	D	X_{26}	E
Nonomuraea sp.	A40926	CAD91223.1	H^{301}-G-H-S-D-H	X_{65}	E	X_{25}	D	X_{26}	E
Streptomyces toyacaensis	A47934	AAM80528.1	H^{301}-G-H-S-D-H	X_{65}	E	X_{25}	D	X_{26}	E
Burkholderia pyrrocinia	Occidiofungin	KFL51884.1	H^{308}-S-H-H-D-H	X_{66}	E	X_{25}	D	X_{26}	E
Lysobacter sp.	Lysobactin	AEH59101.1	H^{303}-P-H-Q-D-H	X_{66}	E	X_{25}	D	X_{26}	E
Burkholderia thailandensis	Bactobolin	ABC35075.1	H^{300}-S-H-H-D-H	X_{66}	E	X_{25}	D	X_{26}	E

Tab. S6 Manual BLASTp search results between chitinopeptin enzymes no. 5+6 and reported enzymes to L-Dap biosynthesis.

Organism	Related products	Homologous enzymes	Protein-ID	Identities (enzyme no. 5/ no. 6)				Positives (enzyme no. 5/ no. 6)				Ref.
				C. eiseniae	*C. flava*	*C. oryziterrae*	*C. niastensis*	*C. eiseniae*	*C. flava*	*C. oryziterrae*	*C. niastensis*	
Bacillus thuringiensis	Zwittermycin A	Zwa5A/Zwa5B	ACM79805/ACM79806	48/	48/52	49/52	49/52	66/	66/73	68/71	66/71	[85]
Staphylococcus aureus	Staphyloferrin B	SbnA/SbnB	TLW72643/TLW72644	37/	38/34	40/34	38/35	57/	58/54	60/53	58/54	[80,81]
Saccharothrix mutabilis	Capreomycin	CmnB/CmnK	ABR67745/ABR67754	33/	32/24	33/24	31/23	50/	49/41	50/41	49/42	[83,84]
Streptomyces sp.	Viomycin	VioB/VioK	AAP92492/AAP92501	30/	26/24	27/24	30/23	47/	47/41	49/40	49/42	[82]
Amycolatopsis japonicum	[S,S]-EDDS	AesC/AesA	AIG74590/AIG74588	25/	26/17	28/18	26/-	45/	44/37	47/37	46/-	[86]
Streptomyces sp.	EDHA	MA_5143a_00503/00505	-	23/	24/23	25/24	24/23	46/	45/52	47/48	45/48	[87]

Tab. S7 Comparison of the amino acid prediction based on Stachelhaus code (88) and the assembled amino acids verified by NMR and Marfey's analysis. Amino acids marked in grey are most likely all L-Dap based on the same Stachelhaus code.

C. eiseniae				*C. flava*				*C. oryziterrae*		*C. niastensis*	
Chitinopeptin A & B		predicted		Chitinopeptin C1+C2 & D1+D2		predicted		predicted		predicted	
NMR		AA	Stachelhaus	NMR		AA	Stachelhaus	AA	Stachelhaus	AA	Stachelhaus
-	-	-	-	2*S*	Dap	hydrophilic	DiWeLtadDK	hydrophilic	DiWeLtadDK	hydrophilic	DiWeLtadDK
2*S*	*N*-Me-Val	Val (nMT)	DAiWmGGTFK	2*S*	*N*-Me-Val	Val (nMT)	DAiWmGGTFK	Val (nMT)	DAiWmGGTFK	hydrophilic	DiWeLtadDK
2*R*,3*R*	Thr	Thr (E)	DFWNIGMVHK	2*R*,3*R*	Thr	Thr (E)	DFWNIGMVHK	Thr (E)	DFWNIGMVHK	Thr (E)	DFWNIGMVHK
2*R*	Ala	Ser (E)	DVWHLSLIeK	2*R*	Ala	Ser (E)	DVWHISLIEK	Ser (E)	DVWHISLIeK	X (Lys?) (E)	DAEhIGeVtK
2*S*,3*S*	β-OH-Asp (Hya)	Asp	DLTKiGHIGK	2*S*,3*S*	β-OH-Asp	Asp	DLTKiGHIGK	Asp	DLTKiGHIGK	Asp	DLTKiGHIGK
-	-	-	-	-	-	-	-	-	-	Tyr	DASTVAAVCK
-	-	-	-	-	-	-	-	-	-	Leu (E)	DAWFLGNVVK
-	-	-	-	-	-	-	-	-	-	Asp	DLTKiGHIGK
2*S*,3*R*	β-OH-Phe	Phe	DAyTVAAVCK	2*S*,3*R*	β-OH-Phe	Phe	DAyTVAAVCK	Phe	DAyTVAAVCK	Phe	DAyTVAAVCK
2*R*,3*S*	Ile	Ile (E)	DAFFIGITFK	2*R*,3*S*	Ile	Ile (E)	DAFFIGITFK	Val (E)	DAiWmGGTFK	-	-
2*S*	Ser	Ser	DVWHLSLIDK	2*S*	Ser	Ser	DVWHLSLIDK	Ser	DVWHLSLIDK	-	-
2*R*	Lys	Tyr (E)	DAedVGEVVK	2*R*	Lys	Tyr (E)	DAedVGEVVK	hydrophilic (E)	DAediGEiVK	-	-
2*S*	Dap	hydrophilic	DiWeLtadDK	2*S*	Dap	Ala	DiWeMtadDK	hydrophilic	DiWeLtadDK	hydrophilic	DiWeLtadDK
2*R*	Leu	Leu (E)	DAWFLGNVVK	2*R*,3*S*	Ile	Ile (E)	DAFFIGITFK	Leu (E)	DAWFLGNVVK	Leu (E)	DAWFLGNVVK
2*S*,3*S*	β-OH-Asp (Hya)	Asp	DLTKiGHIGK	2*S*,3*S*	β-OH-Asp	Asp	DLTKVGHIGK	Asp	DLTKiGHIGK	Asp	DLTKVGHIGK
2*S*	Leu	Leu	DAWFLGNVVK	2*S*	Leu	Leu	DAWFLGNVVK	Leu	DAWFLGNVVK	Ala	DiWeLtadDK
2*S*,3*S*	β-OH-Asp (Hya)	Asp	DLTKiGHIGK	2*S*,3*S*	β-OH-Asp	Asn	DLTKIGhVGK	Asp	DLTKiGHIGK	Asp	DLTKVGHIGK
-	-	-	-	-	-	-	-	-	-	Gly	DILQLGLIWK
2*S*,3*R*	β-OH-Ile	Ile	DAFFIGITFK	2*S*,3*R*	β-OH-Ile	Ile	DAFFIGITFK	Ile	DAFFIGITFK	Ile	DAFw LGVTFK

4. Synthesis of β-hydroxyamino acids (Phe, Ile, Asp)

4.1. Synthetic procedures and compound characterization

4.1.1. General Methods

All chemicals and solvents/anhydrous solvents were commercially supplied and used without further purification. Reactions were monitored using thin layer chromatography (TLC) or using Agilent 1100 series LCMS with UV detection at 254 nm and a low resonance electrospray mode (ESI). TLC was performed on pre-coated silica gel glass plates (Merck TLC Silica gel 60 F254) and compounds were detected under UV light (254 nm) and/or by staining with an aqueous solution of Phosphomolybdic acid, Cerium(IV) sulfate and H_2SO_4 followed by heating with a heat gun. Products were purified by using an automated flash column chromatography system (puriFlash® XS520Plus from Intechim) equipped with PF-15SIHC flash columns of different sizes from Interchim (eluants are given in parentheses). Alternatively, purification was performed by HPLC with a Waters AutoPurification HPLC/MS system using RP-18 columns with H_2O/TFA 0.1% as mobile phase A and acetonitrile as mobile phase B. The product containing fractions were collected and freeze-dried. NMR spectra were recorded on a Bruker AVANCE II WB spectrometer (400 MHz), a AVANCE III HD spectrometer (400 MHz) or a AVANCE III spectrometer (500 MHz) equipped with a TCI CryoProbe with $CDCl_3$, D_2O or DMSO-d_6 as solvent with chemical shifts (δ) quoted in parts per million (ppm) and referenced to the solvent signal ($\delta^1H/^{13}C$: $CDCl_3$ 7.26/77.16, δ^1H: D_2O 4.79, $\delta^1H/^{13}C$: DMSO-d_6 2.50 / 39.52 ppm) or TSPA ($\delta^1H/^{13}C$: 0.00/0.00) as external standard. Assignment was confirmed based on COSY, ROESY, HSQC and HMBC correlations. High resolution mass spectrometry was performed on a maXis II (Bruker) ESI TOF MS equipped with 1290 UPLC (Agilent) with DAD and ELSD. Specific rotation was measured by a polarimeter (P 3000 series) from Krüss.

4.1.2. Preparation of the β-hydroxyaspartic acids

All four stereoisomers of β-hydroxyaspartic acid were obtained according to a modified procedure described by Breuning *et al.*[1] Commercial available (−)-dibenzyl D-tartrate or (+)-dibenzyl L-tartrate were chosen as starting material since the benzyl esters allow for accurate *ee*-determination by chiral HPLC (due to their UV absorption at a detection wavelength of 207 nm) and traceless deprotection accompanied by azide reduction *via* catalytic hydrogenation as a last step. While the *anti*-isomers are directly accessible, the *syn*-isomers were obtained by selective base-induced epimerization of the azido-intermediates and separation of the two isomers by HPLC (Scheme S1). The high measured *ee* values of the products **SI2**, **SI4**, ***ent*-SI2** and ***ent*-SI4** proved a selective epimerization at the azido-bearing 2-position.

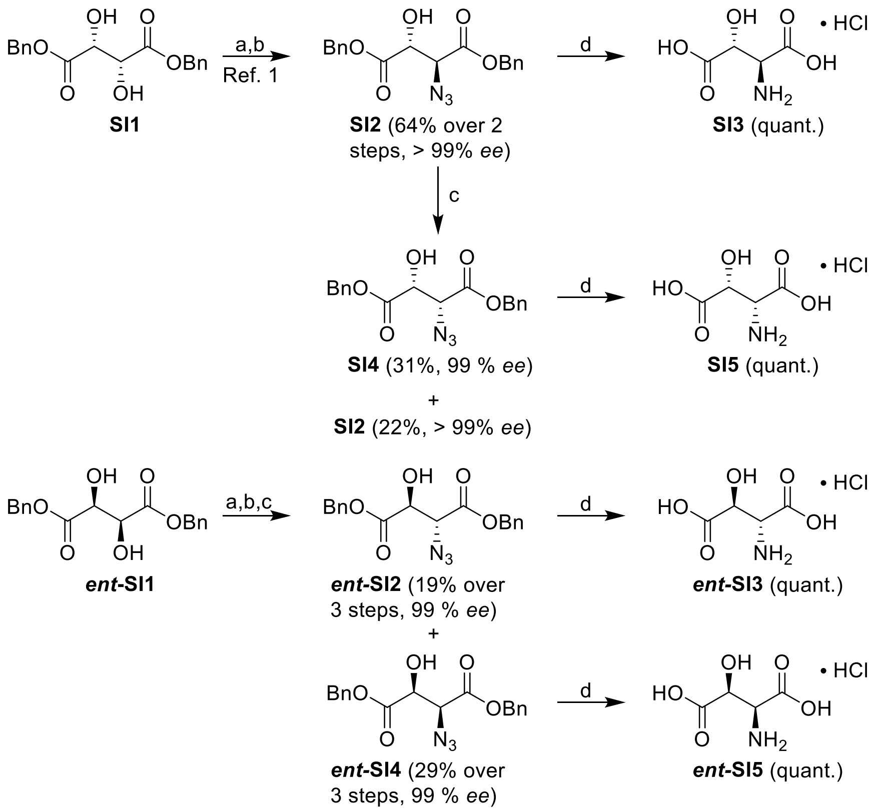

***Scheme S1*: Preparation of the β-hydroxyaspartic acids, exemplarily shown for one diastereomeric series.[a]**

[a] Conditions: a) $SOCl_2$, DCM; b) NaN_3, DMF; c) DBU, THF, 40 °C; d) H_2, Pd/C; 1 M HCl_{aq}, 1,4-dioxane.

[1] Breuning, A., Vicik, R. & Schirmeister, T. An improved synthesis of aziridine-2,3-dicarboxylates via azido alcohols—epimerization studies. *Tetrahedron: Asymmetry* **14,** 3301–3312; 10.1016/j.tetasy.2003.09.015 (2003).

Dibenzyl (2*S*,3*R*)-2-azido-3-hydroxysuccinate (SI2):

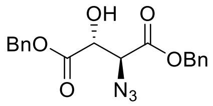

SI2

$SOCl_2$ (0.389 mL, 5.45 mmol, 1.20 eq.) was added to a solution of (+)-dibenzyl L –tartrate (**SI1**, 1.50 g, 4.54 mmol, 1.00 eq.) in anhydrous DCM (20 mL) at room temperature. A catalytic amount of anhydrous DMF (1 drop) was added and the reaction mixture was stirred for 7 h, until LC-MS indicated complete conversion. The reaction mixture was concentrated *in vacuo* and crude cyclic sulfite was used for the next step without further purification.

The crude product was dissolved in anhydrous DMF (4 mL), NaN_3 (0.590 g, 9.08 mmol, 2.00 eq.) was added and the reaction mixture was stirred overnight at room temperature, until LC-MS indicated complete conversion. DCM (10 mL) and H_2O (3 mL) were added and the reaction mixture was allowed to stir for 2 h. Ethyl acetate (50 mL) was added and the mixture was washed with saturated aqueous NaCl (2 x 20 mL), dried over $MgSO_4$, filtered and concentrated under reduced pressure. The crude product was purified by flash column chromatography (0–50% ethyl acetate in *n*-heptane) to give **SI2** (1.04 g, 2.92 mmol, 64% over two steps) as colorless solid.

^{1}H-NMR ($CDCl_3$, 400 MHz): 7.38–7.31 (m, 6H, aryl-*H*), 7.31–7.35 (m, 4H, aryl-*H*), 5.13 (dd, 1H, *J* = 11.9 Hz, CH_2), 5.08 (s, 2H, CH_2), 5.02 (dd, 1H, *J* = 11.9 Hz, CH_2), 4.68 (d, 1H, *J* = 2.7 Hz, C*H*), 4.37 (d, 1H, *J* = 2.7 Hz, C*H*), 3.15 (s, br, 1H, O*H*); **^{13}C-NMR** ($CDCl_3$, 100 MHz): 170.7 (*C*O), 166.9 (*C*O), 134.6 (aryl-C_q), 134.4 (aryl-C_q), 129.0 (aryl-*C*), 128.9 (aryl-*C*), 128.9 (aryl-*C*), 128.8 (aryl-*C*), 128.8 (aryl-*C*), 72.2 (*C*H), 68.6 (*C*H_2), 68.3 (*C*H_2), 64.5 (*C*H); **HRMS (ESI)** m/z calcd. for $C_{18}H_{17}N_3O_5Na$: $(M+Na)^+$, 378.1060; found: 378.1067 $(M+Na)^+$; **R_f** (*n*-heptane/ethyl acetate 2:1): 0.37; **Chiral HPLC** (Chiralpak AS-H/122, 250x4.6 mm; EtOH:MeOH 1:1): **SI2** (Rt = 3.9 min) : ***ent*-SI2** (Rt = 4.3 min) 100 : 0 (> 99% *ee*); **Specific rotation** $[\alpha]_D^{20.6}$ = + 19.8 (c = 1.11; $CHCl_3$).

Dibenzyl (2*S*,3*R*)-2-azido-3-hydroxysuccinate (SI2) and dibenzyl (2*R*,3*R*)-2-azido-3-hydroxysuccinate (SI4):

SI2 (0.300 g, 0.844 mmol, 1.00 eq.) was dissolved in anhydrous THF (10 mL), DBU (0.382 mL, 2.53 mmol, 3.00 eq.) was added and the reaction mixture was stirred at 40 °C. After 1 h the epimerization was stopped by addition of ethyl acetate (50 mL) and 1 M aqueous HCl (10 mL). The layers were separated, the organic layer was washed with saturated aqueous NaCl (10 mL), dried over $MgSO_4$, filtered and concentrated

under reduced pressure. The crude product was purified *via* HPLC (RP-18, 15 min, 50-80% MeCN in H_2O/TFA 0.1%) to yield **SI2** (0.065 g, 0.18 mmol, 22%) as colorless solid and **SI4** (0.092 g, 0.26 mmol, 31%) as colorless oil.

Dibenzyl (2*R*,3*R*)-2-azido-3-hydroxysuccinate (SI4)

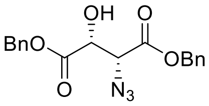

SI4

^{1}H-NMR ($CDCl_3$, 400 MHz): 7.42–7.32 (m, 10H, aryl-*H*), 5.28 (s, 2H, C*H*$_2$), 5.27 (d, 2H, J = 2.1 Hz, C*H*$_2$), 4.81 (d, 1H, J = 2.3 Hz, C*H*), 4.25 (d, 1H, J = 2.3 Hz, C*H*), 3.20 (s, br, 1H, O*H*); **^{13}C-NMR** ($CDCl_3$, 100 MHz): 171.1 (*C*O), 167.4 (*C*O), 134.8 (aryl-C_q), 134.6 (aryl-C_q), 129.1 (aryl-*C*), 128.9 (aryl-*C*), 128.8 (aryl-*C*), 128.7 (aryl-*C*), 128.6 (aryl-*C*), 72.2 (*C*H), 68.6 (*C*H_2), 68.3 (*C*H_2), 63.5 (*C*H); **HRMS (ESI)** m/z calcd. for $C_{18}H_{17}N_3O_5Na$: $(M+Na)^+$, 378.1060; found: 378.1065 $(M+Na)^+$; **R_f** (*n*-heptane/ethyl acetate 2:1): 0.38; **Chiral HPLC** (Chiralpak AS-H/148, 250x4.6 mm; EtOH:MeOH 1:1): **SI4** (Rt = 6.8 min) : ***ent*-SI4** (Rt = 5.5 min) 99.3 : 0.7 (99% *ee*); **Specific rotation** $[\alpha]_D^{20.6}$ = + 59.9 (c = 0.87; $CHCl_3$).

Dibenzyl (2*R*,3*S*)-2-azido-3-hydroxysuccinate (*ent*-SI2) and dibenzyl (2*S*,3*S*)-2-azido-3-hydroxysuccinate (*ent*-SI4):

$SOCl_2$ (0.389 mL, 5.45 mmol, 1.20 eq.) was added to a solution of (−)-dibenzyl D-tartrate (***ent*-SI1**, 1.50 g, 4.54 mmol, 1.00 eq.) in anhydrous DCM (20 mL) at room temperature. A catalytic amount of anhydrous DMF (1 drop) was added and the reaction mixture was stirred for 7 h, until LC-MS indicated complete conversion. The reaction mixture was concentrated *in vacuo* and crude cyclic sulfite was used for the next step without further purification.

The crude product was dissolved in anhydrous DMF (4 mL), NaN_3 (0.590 g, 9.08 mmol, 2.00 eq.) was added and the reaction mixture was stirred overnight at room temperature, until LC-MS indicated complete conversion. DCM (10 mL) and H_2O (3 mL) were added and the reaction mixture was allowed to stir for 2 h. Ethyl acetate (50 mL) was added and the mixture was washed with saturated aqueous NaCl (2 x 20 mL), dried over $MgSO_4$, filtered and concentrated under reduced pressure. The crude product (1.62 g, 4.54 mmol), quant.) was divided in two parts: 400 mg (1.13 mmol) were used for the epimerization (see below), the rest (1.22 g, 3.41 mmol) was purified *via* HPLC (RP-18, 15 min, 55-75% MeCN in H_2O/TFA 0.1%) to yield ***ent*-SI2** (0.470 g, 1.32 mmol, 39% over two steps) as colorless solid.

Crude ***ent*-SI2** (0.400 g, 1.13 mmol, 1.00 eq.) was dissolved in anhydrous THF (10 mL), DBU (0.509 mL, 3.38 mmol, 3.00 eq.) was added and the reaction mixture was stirred at 40 °C. After 1 h the epimerization was stopped by addition of ethyl acetate (50 mL) and 1 M aqueous HCl (10 mL). The layers were separated, the organic layer was washed with saturated aqueous NaCl (10 mL), dried over $MgSO_4$, filtered and concentrated under reduced pressure. The crude product was purified *via* HPLC (RP-18, 15 min, 60-70% MeCN in H_2O/TFA 0.1%) to yield ***ent*-SI2** (0.077 g, 0.22 mmol, 19% over three steps) as colorless solid and ***ent*-SI4** (0.115 g, 0.324 mmol, 29% over three steps) as colorless oil.

Dibenzyl (2*R*,3*S*)-2-azido-3-hydroxysuccinate (*ent*-SI2)

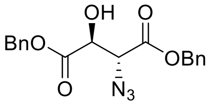

***ent*-SI2**

The NMR data are identical to the ones reported for **SI2**.

HRMS (ESI) m/z calcd. for $C_{18}H_{17}N_3O_5Na$: $(M+Na)^+$, 378.1060; found: 378.1066 $(M+Na)^+$; **R_f** (*n*-heptane/ethyl acetate 2:1): 0.37; **Chiral HPLC** (Chiralpak AS-H/122, 250x4.6 mm; EtOH:MeOH 1:1): ***ent*-SI2** (Rt = 4.3 min) : **SI2** (Rt = 3.9 min): 99.5 : 0.50 (99% *ee*); **Specific rotation** $[\alpha]_D^{20.6}$ = − 19.3 (c = 1.34; $CHCl_3$).

Dibenzyl (2*S*,3*S*)-2-azido-3-hydroxysuccinate (*ent*-SI4)

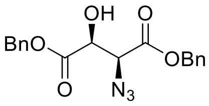

***ent*-SI4**

The NMR data are identical to the ones reported for **SI4**.

HRMS (ESI) m/z calcd. for $C_{18}H_{17}N_3O_5Na$: $(M+Na)^+$, 378.1060; found: 378.1065 $(M+Na)^+$; **R_f** (*n*-heptane/ethyl acetate 2:1): 0.38; **Chiral HPLC** (Chiralpak AS-H/148, 250x4.6 mm; EtOH:MeOH 1:1): ***ent*-SI4** (Rt = 5.5 min) : **SI4** (Rt = 6.8 min) 99.7 : 0.3 (99% *ee*); **Specific rotation** $[\alpha]_D^{20.6}$ = − 67.9 (c = 0.68; $CHCl_3$).

General procedure for hydrogenation:

The acid **SI2**, ***ent*-SI2**, **SI4** or ***ent*-SI4** (0.1–0.2 mmol) was dissolved in 1,4-dioxane (5 mL) and 1 M aqueous HCl (1 mL), Pd/C (10 %, 0.05 eq.) was added and the reaction mixture was hydrogenated at 4 bar pressure in an autoclave at room temperature. After reaction overnight, LC-MS indicated complete hydrogenation to **SI3**, ***ent*-SI3**, **SI5** or ***ent*-SI5**, respectively. The reaction mixture was filtered, diluted with H_2O (20 mL) and freeze-dried to yield the desired product (HCl salt, quant.) as colourless solid.

(2*S*,3*R*)-2-Amino-3-hydroxysuccinic acid ((2*S*,3*R*)-3-hydroxyaspartic acid) (SI3):

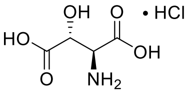

SI3

^{1}H-NMR (D_2O, 400 MHz): 4.73 (d, 1H, *J* = 2.7 Hz, C*H*), 4.59 (d, 1H, *J* = 2.7 Hz, C*H*); **^{13}C-NMR** (D_2O, 100 MHz): 176.1 (*C*OOH), 71.7 (*C*H), 58.5 (*C*H); **HRMS (ESI)** m/z calcd. for $C_4H_8NO_5$: $(M+H)^+$, 150.0397; found: 150.0397 $(M+H)^+$; **Specific rotation** $[\alpha]_D^{20.8}$ = + 46.6 (c = 1.03; H_2O).

(2*R*,3*S*)-2-Amino-3-hydroxysuccinic acid ((2*R*,3*S*)-3-hydroxyaspartic acid) (*ent*-SI3):

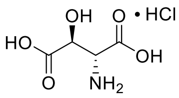

***ent*-SI3**

The NMR data are identical to the ones reported for **SI3**.

HRMS (ESI) m/z calcd. for $C_4H_8NO_5$: $(M+H)^+$, 150.0397; found: 150.0397 $(M+H)^+$; **Specific rotation** $[\alpha]_D^{20.7}$ = − 44.3 (c = 0.65; H_2O).

(2*R*,3*R*)-2-Amino-3-hydroxysuccinic acid ((2*R*,3*R*)-3-hydroxyaspartic acid) (SI5):

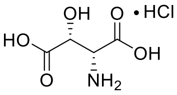

SI5

^{1}H-NMR (D_2O, 400 MHz): 4.86 (d, 1H, *J* = 2.4 Hz, C*H*), 4.40 (d, 1H, *J* = 2.4 Hz, C*H*); **^{13}C-NMR** (D_2O, 100 MHz): 71.5 (*C*H), 58.4 (*C*H); **HRMS (ESI)** m/z calcd. for $C_4H_8NO_5$: $(M+H)^+$, 150.0397; found: 150.0398 $(M+H)^+$; **Specific rotation** $[\alpha]_D^{20.8}$ = + 9.7 (c = 0.51; H_2O).

(2*S*,3*S*)-2-Amino-3-hydroxysuccinic acid ((2*S*,3*S*)-3-hydroxyaspartic acid) (*ent*-SI5):

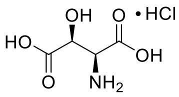

***ent*-SI5**

The NMR data are identical to the ones reported for **SI5**.

HRMS (ESI) m/z calcd. for $C_4H_8NO_5$: $(M+H)^+$, 150.0397; found: 150.0397 $(M+H)^+$; **Specific rotation** $[\alpha]_D^{20.6}$ = − 8.0 (c = 0.63; H_2O).

4.1.3. Preparation of the β-hydroxyphenylalanines

The L-isomers of the β-hydroxyphenylalanines and β-hydroxyisoleucines were synthesized by diastereoselective addition of Grignard reagents to orthoester protected L-serine aldehyde **SI7**, initially described by Blaskovich and Lajoie[2]. This synthetic pathway offers several advantages for our envisaged access to reference samples of these β-hydroxyamino acids:

- High *threo*-selectivities are reported for the addition of Grignard reagents to aldehyde **SI7** and ketones **SI18** and **SI25** (Scheme S2 and Scheme S3).
- The addition products are bearing the oxidation stage of the desired amino acids and only protecting group manipulations are required to obtain the reference samples. In contrast to the commonly used synthetic strategy for β-hydroxyamino acids starting from Garner's Aldehyde[3,4], no protection of the secondary alcohol and no further oxidation step are required.
- The Cbz-group allows for accurate *ee*-determination by chiral HPLC (due to their UV absorption at a detection wavelength of 207 nm) and convenient deprotection *via* catalytic hydrogenation or under acidic conditions at the last step.
- The epimerization of aldehyde **SI7** *via* simple flash chromatography on silica exhibits a practical access to racemic reference samples which are required for the *ee*-determination by chiral HPLC and to cover the corresponding *R*-series of the β-hydroxyamino acids.

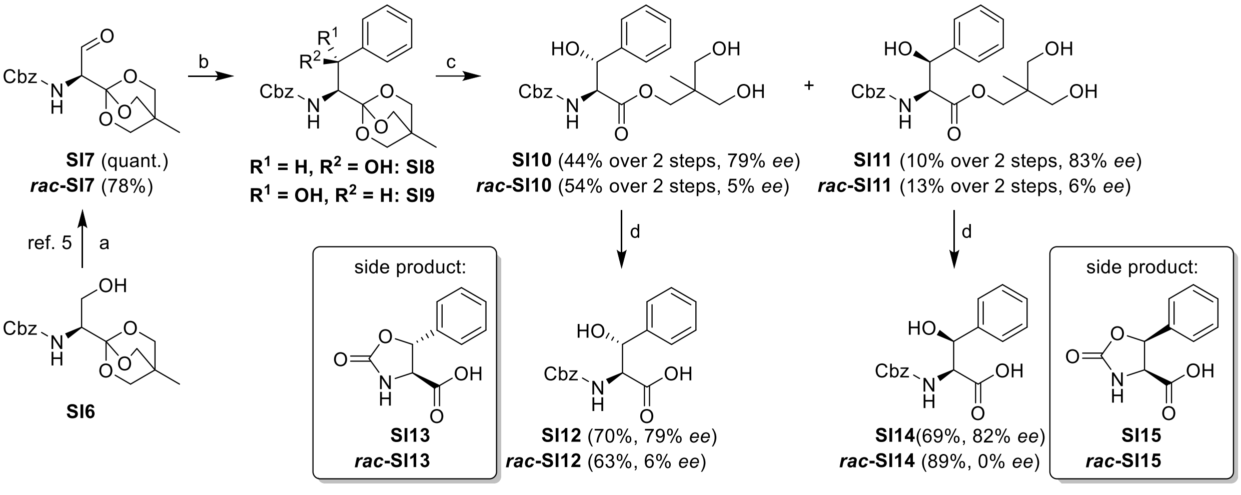

***Scheme S2.* Preparation of β-hydroxyphenylalanines.[a]**

---

[2] Blaskovich, M. A. *et al.* Stereoselective Synthesis of Threo and Erythro β-Hydroxy and β-Disubstituted-β-Hydroxy α-Amino Acids. *J. Org. Chem.* **63,** 3631–3646 (1998).
[3] Garner, P. Stereocontrolled addition to a penaldic acid equivalent: an asymmetric of threo-β-hydroxy-L-glutamic acid. *Tetrahedron Lett*. **25,** 5855–5858 (1984).
[4] Passiniemi, M. & Koskinen A. M. P. Garner's aldehyde as a versatile intermediate in the synthesis of enantiopure natural products. *Beilstein J. Org. Chem*. **9,** 2641–2659 (2013).

[a] Conditions: a) $(COCl)_2$, DMSO, DCM, −78 °C, 15 min; **SI6**, −78 °C, 90 min; DIPEA, −78 °C to 0 °C, 30 min; **SI7** used as a crude; ***rac*-SI7** purified by flash column chromatography; b) PhMgBr, THF, rt; c) 1 M HCl_{aq}, 1,4-dioxane; d) LiOH, MeCN/H_2O 4:1.

The synthesis of **SI7** and ***rac*-SI7** was performed in close analogy to a literature known procedure from **SI6**[5]. To obtain both diastereomers in one reaction and in sufficient quantities, the addition of PhMgBr was performed at room temperature (Scheme S2). Under these conditions, a selectivity of 84:16 for **SI8**:**SI9** is described[2]. In our hands, complete separation of **SI8** and **SI9** *via* flash chromatography was difficult on 1 g-scale. Therefore, the mixture of **SI8** and **SI9** was directly treated by aqueous HCl to achieve orthoester hydrolysis and resulting products **SI10** and **SI11** were separated by preparative HPLC. Previous orthoester hydrolysis is required since the OBO-orthoesters are unstable to acidic HPLC conditions. Contrary to Ref. 2, we performed a saponification of **SI10** and **SI11** to Cbz-protected amino acids **SI12** and **SI14** to obtain UV-active compounds for *ee*-determination. Careful monitoring of the base equivalents and the reaction progress is required since an excess of LiOH resulted in formation of cyclic carbamates **SI13** and **SI15**, respectively.[6] However, we were able to suppress this side reaction to an acceptable level. For the Marfey-protocol, both the ester **SI10** and **SI11** and their corresponding acids **SI12** and **SI14** were suitable because the acidic conditions cleave the Cbz-proteting group and the ester. In our synthesis, we achieved acceptable *ee*s for the *S*-series (79-83%) and the epimerization of **SI7** *via* flash chromatography was successful (*ee*s from 0-6% for the *rac*-series).

Benzyl (*S*)-(1-(4-methyl-2,6,7-trioxabicyclo[2.2.2]octan-1-yl)-2-oxoethyl)carbamate (SI7):

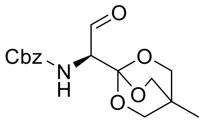

SI7

SI7 was synthesized according to a slightly modified literature-known procedure[5]: The reaction was carried out in moisture-free glassware under inert atmosphere. To a solution of $(COCl)_2$ (0.441 mL, 5.11 mmol, 1.70 eq.) in anhydrous DCM (20 mL) anhydrous DMSO

[5] Rose, N. G. W. *et al.* Preparation of 1-[N-Benzyloxycarbonyl-(1S)-1-amino-2-oxoethyl]-4-methyl-2,6,7-trioxabicyclo-[2.2.2]octane. *Organic Syntheses, Coll. Vol. 10,* 73 (2004); *Vol. 79*, 216 (2002).
[6] The carbamates were only isolated for the racemic series for characterization.

(0.683 mL, 9.62 mmol, 3.20 eq.) was added carefully at −78 °C. After stirring for 15 min, alcohol **SI6** (0.972 g, 3.01 mmol, 1.00 eq.) dissolved in anhydrous DCM (20 mL) was added. The reaction mixture was stirred for 1.5 h at −78 °C. Then DIPEA (3.30 mL, 18.7 mmol, 6.23 eq.) was added and the solution was stirred for 30 min at −78 °C and for further 30 min without cooling bath. A mixture of cooled toluene/saturated aqueous NH_4Cl (4:1, 250 mL, pre-cooled to 0 °C) was added to the reaction mixture. The layers were separated and the organic layer was washed with saturated aqueous NH_4Cl (3 x 50 mL, pre-cooled to 0 °C), with saturated aqueous $NaHCO_3$ (50 mL, pre-cooled to 0 °C), and with saturated aqueous NaCl (50 mL, pre-cooled to 0 °C). The organic layer was dried over $MgSO_4$, filtered, and concentrated under reduced pressure to yield crude aldehyde **SI7** (1.00 g, max. 3.01 mmol, quant.) as slightly yellow oil which was used in the next stage without further purification.

***Rac*-SI7** was synthesized starting from **SI6** in a similar manner with an additional purification step via chromatography, which led to full epimerization of the stereocenter[7]: The reaction was carried out in moisture-free glassware under inert atmosphere. To a solution of $(COCl)_2$ (1.22 mL, 14.0 mmol, 1.70 eq.) in anhydrous DCM (60 mL) anhydrous DMSO (2.09 mL, 30.0 mmol, 3.60 eq.) was added carefully at −78 °C. After stirring for 15 min, alcohol **SI6** (2.65 g, 8.21 mmol, 1.00 eq.) dissolved in anhydrous DCM (60 mL) was added. The reaction mixture was stirred for 1.5 h at −78 °C. Then DIPEA (9.00 mL, 51.2 mmol, 6.23 eq.) was added and the solution was stirred for 30 min at −78 °C and for further 30 min without cooling bath. Saturated aqueous NH_4Cl (100 mL) and DCM (100 mL) were added to the reaction mixture, the layers were separated and the organic layer was washed with saturated aqueous NH_4Cl (50 mL), with saturated aqueous $NaHCO_3$ (50 mL), and with saturated aqueous NaCl (50 mL). The organic layer was dried over $MgSO_4$, filtered, and concentrated under reduced pressure. The crude product was purified by flash column chromatography[8] (100% DCM, then DCM : EtOAc 4:1) to give ***rac*-SI7** (2.06 g, 6.40 mmol, 78 %) as a colorless solid and was directly used for the next step.

---

[7] The epimerization of the stereocenter of OBO-protected serinals such as **SI7** *via* chromatography is a known phenomenon and was described by Blascovich and Lajoie (Blaskovich, M. A. & Lajoie, G. A. Synthesis of a chiral serine aldehyde equivalent and its conversion to chiral .alpha.-amino acid derivatives. *J. Am. Chem. Soc.* **115,** 5021–5030 (1993).) In our case, the purification *via* column chromatography resulted in measured *ee*-values of 0–6% for the *rac*-series.

[8] Conditioning of the silica column was performed with a mixture of DCM and NEt_3 (98:2) to neutralize the slightly acidic silica and to prevent partial orthoester hydrolysis during chromatography.

R_f (*n*-heptane/ethyl acetate 1:3): 0.65

3-Hydroxy-2-(hydroxymethyl)-2-methylpropyl (2*S*,3*R*)-2-(((benzyloxy)carbonyl)amino)-3-hydroxy-3-phenylpro-panoate (SI10) and 3-Hydroxy-2-(hydroxymethyl)-2-methylpropyl (2*S*,3*S*)-2-(((benzyloxy)carbonyl)amino)-3-hydroxy-3-phenylpro-panoate (SI11):

The reaction was carried out in moisture-free glassware under inert atmosphere. To a solution of aldehyde **SI7** (0.965 g, 3.00 mmol, 1.00 eq.) in anhydrous THF (30 mL) was added PhMgBr (1 M in THF, 12.0 mL, 12.0 mmol, 4.00 eq.) at room temperature. After 90 min, TLC indicated full conversion of the starting material (R_f of **SI8**/**SI9** (*n*-heptane/ethyl acetate 1:3): 0.60). Saturated aqueous NH_4Cl (20 mL) and EtOAc (100 mL) were added to the reaction mixture, the layers were separated and the organic layer was washed with saturated aqueous NaCl (20 mL). The organic layer was dried over $MgSO_4$, filtered, and concentrated under reduced pressure. The crude mixture of **SI8**/**SI9** was dissolved in 1,4-dioxane (20 mL) and aqueous HCl (1 N, 1 mL) and the reaction mixture was stirred for 30 min until full hydrolysis of the orthoester was observed by TLC. All volatiles were removed under reduced pressure and the residue was purified *via* HPLC (RP-18, 15 min, 35-45% MeCN in H_2O/TFA 0.1%) to yield **SI10** (0.549 g, 1.32 mmol, 44% over two steps) and **SI11** (0.124 g, 0.297 mmol, 10% over two steps) as colorless oils.

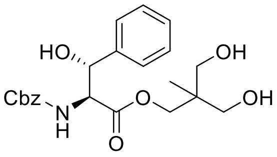

SI10

For **SI10**: **^{1}H-NMR** (DMSO-d_6, 400 MHz): 7.42–7.19 (m, 11H, N*H*, aryl-*H*), 5.69 (d, 1H, *J* = 6.2 Hz, CH-O*H*), 5.11 (dd, 1H, *J* = 6.1, 3.9 Hz, C*H*-OH), 4.95 (s, 2H, Ph-CH_2), 4.47 (m, 2H, CH_2-O*H*), 4.40 (dd, 1H, *J* = 9.3, 3.7 Hz, C*H*-NH), 3.96 (d, 1H, *J* = 10.5 Hz, CH_2), 3.90 (d, 1H, *J* = 10.5 Hz, CH_2), 3.30–3.21 (m, 4H, CH_2-OH), 0.77 (s, 3H, CH_3); **^{13}C-NMR** (DMSO-d_6, 100 MHz): 170.4 (*C*O), 156.2 (*C*O-NH), 141.6 (aryl-C_q), 136.9 (aryl-C_q), 128.3 (aryl-*C*), 127.9 (aryl-*C*), 127.7 (aryl-*C*), 127.3 (aryl-*C*), 127.2 (aryl-*C*), 126.1 (aryl-*C*), 72.3 (*C*H-OH), 67.1 (CH_2), 65.4 (Ph-CH_2), 63.5 (CH_2-OH), 60.5 (*C*H-NH), 40.7 (C_q-CH_3), 16.3 (CH_3); **HRMS (ESI)** m/z calcd. for $C_{22}H_{27}NO_7Na$: $(M+Na)^+$, 440.1680; found: 440.1685 $(M+Na)^+$; **R_f** (*n*-heptane/ethyl acetate 1:3): 0.25; **Chiral HPLC** (Chiralpak ID/174, 250x4.6 mm; *n*-heptane:EtOH:MeOH 5:1:1 + 0.1% TFA): **SI10** (Rt = 8.6 min) : ***ent*-SI10** (Rt = 5.6 min) 89.3 : 10.7 (79% *ee*); **Specific rotation** $[\alpha]_D^{20.0}$ = − 35.1 (c = 0.29; MeCN).

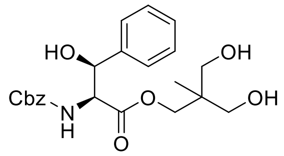

SI11

For **SI11**: **^{1}H-NMR** (DMSO-d$_6$, 400 MHz): 7.64 (d, 1H, *J* = 9.2 Hz, N*H*), 7.42–7.37 (m, 2H, aryl-*H*), 7.35–7.23 (m, 6H, aryl-*H*), 7.21–7.10 (m, 2H, aryl-*H*), 5.76 (d, 1H, *J* = 4.7 Hz, CH-O*H*), 4.93 (d, 1H, *J* = 13.4 Hz, Ph-C*H*$_2$), 4.90 (d, 1H, *J* = 13.4 Hz, Ph-C*H*$_2$), 4.76 (dd, 1H, *J* = 8.3, 4.2 Hz, C*H*-OH), 4.42 (s, br, 2H, CH$_2$-O*H*), 4.24 (t, 1H, *J* = 8.9 Hz, C*H*-NH), 3.96 (d, 1H, *J* = 10.6 Hz, C*H*$_2$), 3.91 (d, 1H, *J* = 10.6 Hz, C*H*$_2$), 3.26 (s, 4H, C*H*$_2$-OH), 0.75 (s, 3H, C*H*$_3$); **^{13}C-NMR** (DMSO-d$_6$, 100 MHz): 170.9 (*C*O), 155.5 (*C*O-NH), 141.9 (aryl-*C*$_q$), 136.8 (aryl-*C*$_q$), 128.3 (aryl-*C*), 127.8 (aryl-*C*), 127.7 (aryl-*C*), 127.4 (aryl-*C*), 127.4 (aryl-*C*), 126.8 (aryl-*C*), 72.3 (*C*H-OH), 66.7 (*C*H$_2$), 65.3 (Ph-*C*H$_2$), 63.5 (*C*H$_2$-OH), 60.5 (*C*H-NH), 40.7 (*C*$_q$-CH$_3$), 16.3 (*C*H$_3$); **HRMS (ESI)** m/z calcd. for $C_{22}H_{27}NO_7Na$: $(M+Na)^+$, 440.1680; found: 440.1685 $(M+Na)^+$; **R$_f$** (*n*-heptane/ethyl acetate 1:3): 0.25; **Chiral HPLC** (Chiralpak IF/181, 250x4.6 mm; *n*-heptane:EtOH:MeOH 5:1:1 + 0.1% TFA): **SI11** (Rt = 13.7 min) : ***ent*-S11** (Rt = 8.5 min) 91.6 : 8.4 (83% *ee*); **Specific rotation** $[\alpha]_D^{19.9}$ = + 6.2 (c = 0.32; MeCN).

***Rac*-SI10** (0.482 g, 1.16 mmol, 54% over two steps) and ***rac*-SI11** (0.118 g, 0.283 mmol, 13% over two steps) were synthesized from ***rac*-SI7** (0.685 g, 2.13 mmol, 1.00 eq.) in analogous manner. The NMR data are identical to the ones reported for **SI10** and **SI11**.

For ***rac*-SI10**: **R$_f$** (*n*-heptane/ethyl acetate 1:3): 0.25; **Chiral HPLC** (Chiralpak ID/174, 250x4.6 mm; *n*-heptane:EtOH:MeOH 5:1:1 + 0.1% TFA): **SI10** (Rt = 8.6 min) : ***ent*-SI10** (Rt = 5.6 min) 52.3 : 47.7 (5% *ee*).

For ***rac*-SI11**: **R$_f$** (*n*-heptane/ethyl acetate 1:3): 0.25; **Chiral HPLC** (Chiralpak IF/181, 250x4.6 mm; *n*-heptane:EtOH:MeOH 5:1:1 + 0.1% TFA): **SI11** (Rt = 14.0 min) : ***ent*-S11** (Rt = 8.5 min) 53.2 : 46.8 (6% *ee*).

(2*S*,3*R*)-2-(((Benzyloxy)carbonyl)amino)-3-hydroxy-3-phenylpropanoic acid (SI12):

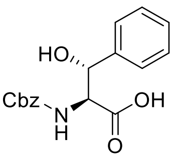

SI12

To a solution of **SI10** (0.509 g, 1.22 mmol, 1.00 eq.) in MeCN/H_2O (4:1, 20.4 mL/5.1 mL) was added LiOH (1 N in H_2O, 1.22 mL, 1.22 mmol, 1.00 eq.) at room temperature. After 20 min, another portion of LiOH (1 N in H_2O, 0.61 mL, 0.61 mmol, 0.50 eq.) was added to reach full conversion of starting material after 30 min monitored by LC-MS. The reaction was stopped by addition of acetic acid (0.1 mL). All volatiles were removed under reduced pressure and the residue was purified *via* HPLC (RP-18, 15 min, 10-55% MeCN in H_2O/TFA 0.1%) to yield **SI12** (0.270 g, 0.856 mmol, 70%) as a colorless solid.[9]

^{1}H-NMR (DMSO-d_6, 400 MHz): 12.81 (s, br, 1H, COO*H*), 7.45–7.19 (m, 10H, aryl-*H*), 7.08 (d, 1H, *J* = 9.5 Hz, N*H*), 5.68 (s, br, 1H, O*H*), 5.16 (s, br., 1H, C*H*-OH), 4.96 (d, 1H, *J* = 13.5 Hz, Ph-CH_2), 4.93 (d, 1H, *J* = 13.5 Hz, Ph-CH_2), 4.33 (dd, 1H, *J* = 9.5, 3.4 Hz, C*H*-NH); **^{13}C-NMR** (DMSO-d_6, 100 MHz): 171.9 (*C*OOH), 156.2 (*C*O-NH), 142.1 (aryl-C_q), 137.0 (aryl-C_q), 128.3 (aryl-*C*), 127.8 (aryl-*C*), 127.7 (aryl-*C*), 127.3 (aryl-*C*), 127.1 (aryl-*C*), 126.2 (aryl-*C*), 72.3 (*C*H-OH), 65.3 (Ph-CH_2), 60.4 (*C*H-NH); **HRMS (ESI)** m/z calcd. for $C_{17}H_{17}NO_5Na$: $(M+Na)^+$, 338.0999; found: 338.1000 $(M+Na)^+$; **Chiral HPLC** (Chiralpak IF/181, 250x4.6 mm; *n*-heptane:EtOH:MeOH 5:1:1 + 0.1% TFA): **SI12** (Rt = 6.8 min) : ***ent*-SI12** (Rt = 5.5 min) 89.7 : 10.3 (79% *ee*); **Specific rotation** $[\alpha]_D^{19.9}$ = − 23.0 (c = 0.65; MeCN).

***Rac*-SI12** (0.210 g, 0.666 mmol, 63%) and ***rac*-SI13** (0.055 g, 0.27 mmol, 25%) were synthesized from ***rac*-SI10** (0.442 g, 1.06 mmol, 1.00 eq.) in analogous manner. The NMR data of ***rac*-SI12** are identical to the ones reported for **SI12**. ***Rac*-SI12** and ***rac*-SI13** were obtained as colorless solids.

For ***rac*-SI12**: **Chiral HPLC** (Chiralpak IF/181, 250x4.6 mm; *n*-heptane:EtOH:MeOH 5:1:1 + 0.1% TFA): **SI12** (Rt = 6.8 min) : ***ent*-SI12** (Rt = 5.5 min) 52.8 : 47.2 (6% *ee*).

(4*S*,5*R*)-2-Oxo-5-phenyloxazolidine-4-carboxylic acid (*rac*-SI13):

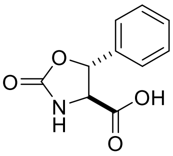

***rac*-SI13**

^{1}H-NMR (DMSO-d_6, 500 MHz): 13.45 (s, br., 1H, COO*H*), 8.36 (s, br., 1H, N*H*), 7.53–7.26 (m, 5H, aryl-*H*), 5.57 (d, 1H, *J* = 4.9 Hz, O-C*H*), 4.23 (dd, 1H, *J* = 4.9, 0.7 Hz, NH-C*H*); **^{13}C-NMR** (DMSO-d_6, 125 MHz): 171.8 (*C*OOH), 157.7 (*C*O), 139.0 (aryl-C_q), 128.8 (aryl-*C*), 128.8 (aryl-*C*), 125.8 (aryl-*C*),

[9] The formation of **SI13** was observed *via* LC-MS, but was not isolated for the *S*-series.

78.4 (O-*C*H), 60.6 (NH-*C*H); **HRMS (ESI)** m/z calcd. for $C_{10}H_9NO_4Na$: $(M+Na)^+$, 230.0424; found: 230.0425 $(M+Na)^+$.

(2*S*,3*S*)-2-(((Benzyloxy)carbonyl)amino)-3-hydroxy-3-phenylpropanoic acid (SI14):

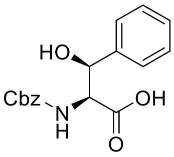

SI14

To a solution of **SI11** (0.083 g, 0.20 mmol, 1.00 eq.) in MeCN/H_2O (4:1, 3.2 mL/0.8 mL) was added LiOH (1 N in H_2O, 0.20 mL, 0.20 mmol, 1.00 eq.) at room temperature. After 20 min, another portion of LiOH (1 N in H_2O, 0.10 mL, 0.10 mmol, 0.50 eq.) was added to reach full conversion of starting material after 30 min monitored by LC-MS. The reaction was stopped by addition of acetic acid (0.1 mL). All volatiles were removed under reduced pressure and the residue was purified *via* HPLC (RP-18, 15 min, 10-55% MeCN in H_2O/TFA 0.1%) to yield **SI14** (0.043 g, 0.14 mmol, 69%) as a colorless solid.[10]

^{1}H-NMR (DMSO-d$_6$, 400 MHz): ~12.51 (s, v. br, 1H, COO*H*), 7.48 (d, 1H, *J* = 9.3 Hz, N*H*), 7.42–7.36 (m, 2H, aryl-*H*), 7.35–7.23 (m, 6H, aryl-*H*), 7.21–7.14 (m, 2H, aryl-*H*), ~5.74 (s, v. br, 1H, O*H*), 4.93 (d, 1H, *J* = 13.3 Hz, Ph-C*H*$_2$), 4.90 (d, 1H, *J* = 13.4 Hz, Ph-C*H*$_2$), 4.73 (d. 1H, *J* = 8.5 Hz, C*H*-OH), 4.15 (t, 1H, *J* = 8.8 Hz, C*H*-NH); **^{13}C-NMR** (DMSO-d$_6$, 100 MHz): 172.3 (*C*OOH), 155.5 (*C*O-NH), 142.2 (aryl-*C*$_q$), 136.9 (aryl-*C*$_q$), 128.3 (aryl-*C*), 127.7 (aryl-*C*), 127.7 (aryl-*C*), 127.4 (aryl-*C*), 127.3 (aryl-*C*), 126.9 (aryl-*C*), 72.4 (*C*H-OH), 65.2 (Ph-*C*H$_2$), 60.4 (*C*H-NH); **HRMS (ESI)** m/z calcd. for $C_{17}H_{17}NO_5Na$: $(M+Na)^+$, 338.0999; found: 338.0998 $(M+Na)^+$; **Chiral HPLC** (Chiralpak IF/181, 250x4.6 mm; *n*-heptane:EtOH:MeOH 5:1:1 + 0.1% TFA): **SI14** (Rt = 5.2 min) : ***ent*-SI14** (Rt = 6.2 min) 90.9 : 9.1 (82% *ee*); **Specific rotation** $[\alpha]_D^{20.0}$ = + 32.3 (c = 0.31; MeCN).

***Rac*-SI14** (0.053 g, 0.17 mmol, 89%) and ***rac*-SI15** (0.002 g, 0.01 mmol, 5%) were synthesized from ***rac*-SI11** (0.079 g, 0.19 mmol, 1.00 eq.) in analogous manner. The NMR data of ***rac*-SI14** are identical to the ones reported for **SI14**. ***Rac*-SI14** and ***rac*-SI15** were obtained as colorless solids.

For ***rac*-SI14**: **Chiral HPLC** (Chiralpak IF/181, 250x4.6 mm; *n*-heptane:EtOH:MeOH 5:1:1 + 0.1% TFA): **SI14** (Rt = 5.2 min) : ***ent*-SI14** (Rt = 6.2 min) 50.2 : 49.8 (0% *ee*).

[10] The formation of **SI15** was observed *via* LC-MS, but was not isolated for the *S*-series.

(4*S*,5*S*)-2-Oxo-5-phenyloxazolidine-4-carboxylic acid (*rac*-SI15):

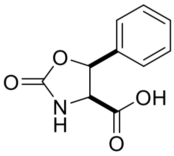

rac-SI15

^{1}H-NMR (DMSO-d$_6$, 500 MHz): 12.71 (s, br., 1H, COO*H*), 8.15 (s, 1H, N*H*), 7.43–7.33 (m, 5H, aryl-*H*), 5.85 (d, 1H, J = 8.9 Hz, O-C*H*), 4.59 (d, 1H, J = 8.9 Hz, NH-C*H*); **^{13}C-NMR** (DMSO-d$_6$, 125 MHz): 170.5 (*C*OOH), 158.4 (*C*O), 135.2 (aryl-*C*$_q$), 128.6 (aryl-*C*), 128.1 (aryl-*C*), 126.4 (aryl-*C*), 77.9 (O-*C*H), 59.3 (NH-*C*H); **HRMS (ESI)** m/z calcd. for $C_{10}H_{10}NO_4$: (M+H)$^+$, 208.0604; found: 208.0604 (M+H)$^+$.

4.1.4. Preparation of the β-hydroxyisoleucines

The synthesis of the β-hydroxyisoleucines was performed in close analogy to the synthesis of the β-hydroxyphenylalanines (see chapter 4.1.3). Addition of MeMgBr and EtMgBr, respectively, to aldehyde **SI7** was performed at room temperature and the crude mixture of the diastereomers **SI16**/**SI17** and **SI23**/**SI24** was subjected to Swern oxidation to yield crude ketones **SI19** and **SI26** (Scheme S3). High diastereoselectivities > 10:1 were achieved at the addition of MeMgBr and EtMgBr, respectively, to ketones **SI19** and **SI26**. The reactions, however, turned out to be slow and therefore were performed at room temperature. For the racemic series, an additional equivalent of Grignard reagent was added after 90 min, which led to a significant improvement of the isolated yields compared to the previously performed *S*-series. Saponifications of **SI20** and **SI27** were accompanied with faster formation of the cyclic carbamates **SI22** and **SI29** compared to the β-hydroxyphenylalanine series (see chapter 4.1.3) but sufficient amounts of target products **SI21** and **SI28** were obtained. Noteworthy, the formation of cyclic carbamate **SI22** is significantly quicker than **SI29**, which resulted in different diastereomeric ratios of the isolated products **SI21** and **SI28**. Neither at the stage of the ester **SI20** and **SI27** nor at the stage of the free carboxylic acids **SI21** and **SI28**, separation of the diastereomers was possible. However, the samples were sufficiently diastereomer-enriched to be used for Marfey's protocol. The ratio of the diastereomers was distinguished by 1H-NMR and chiral HPLC.

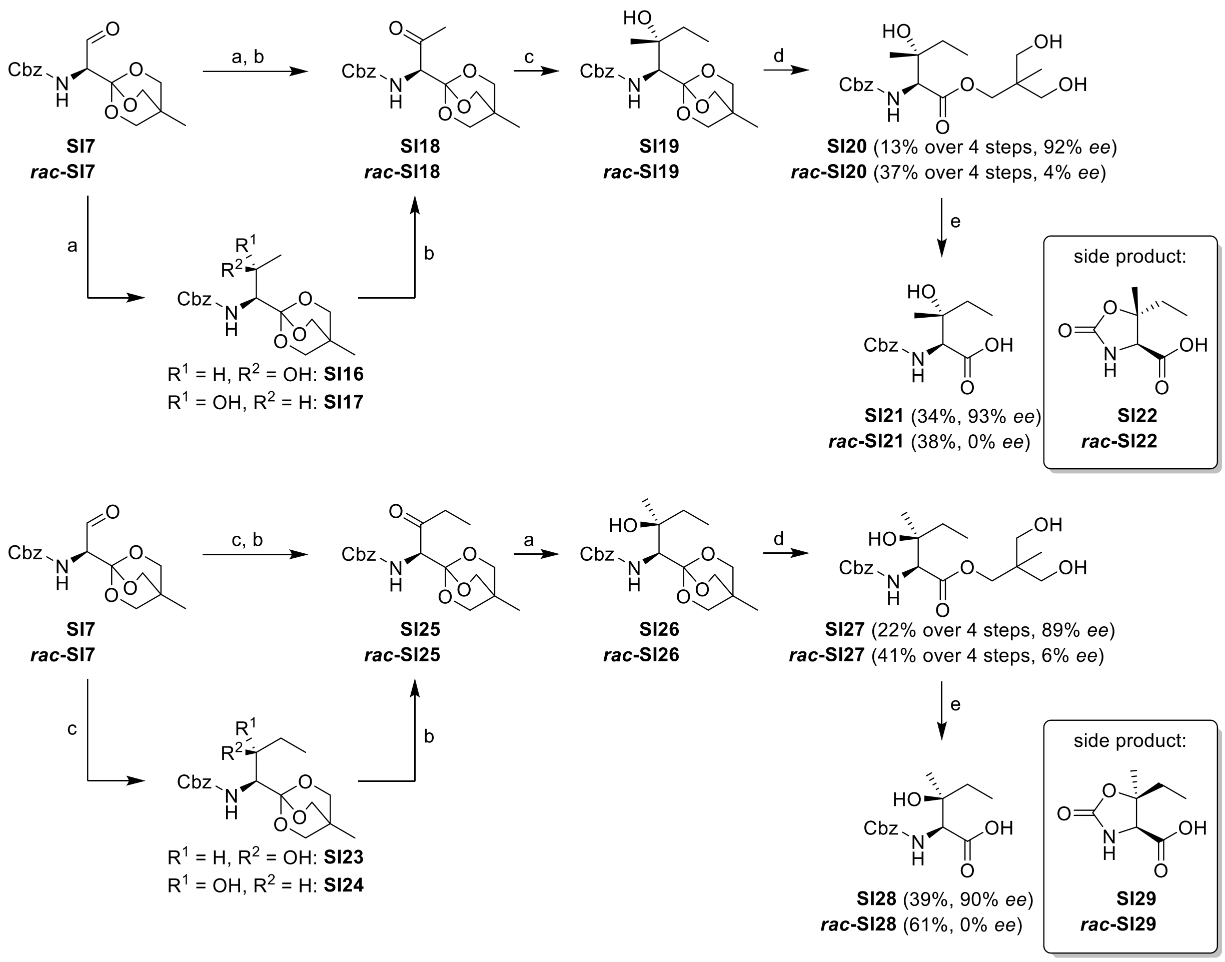

Scheme S3. **Preparation of β-hydroxyisoleucines.**[a]

[a] Conditions: a) MeMgBr, THF, rt; b) $(COCl)_2$, DMSO, DCM, −78 °C, 15 min; **SI16/SI17** or **SI23/SI24** or the corresponding racemic mixtures, −78 °C, 90 min; DIPEA, −78 °C to 0 °C, 30 min; **SI18**, ***rac*-SI18**, **SI25** or ***rac*-SI25** used as a crude; c) EtMgBr, THF, rt; d) 1 M HCl_{aq}, 1,4-dioxane; e) LiOH, MeCN/H_2O 4:1.

3-Hydroxy-2-(hydroxymethyl)-2-methylpropyl (2*S*,3*R*)-2-(((benzyloxy)carbonyl)amino)-3-hydroxy-3-methylpenta-oate (SI20):

The reaction was carried out in moisture-free glassware under inert atmosphere. To a solution of aldehyde **SI7** (0.830 g, 2.58 mmol, 1.00 eq.) in anhydrous THF (30 mL) was added MeMgBr (3 M in Et_2O, 3.44 mL, 10.3 mmol, 4.00 eq.) at room temperature. After 90 min, TLC indicated full conversion of the starting material (R_f of **SI16**/**SI17** (*n*-heptane/ethyl acetate 1:3): 0.48). Saturated aqueous NH_4Cl (20 mL) and EtOAc (100 mL) were added to the reaction mixture, the layers were separated and the organic layer was washed with saturated aqueous NaCl (20 mL). The organic layer was dried over $MgSO_4$, filtered, and concentrated under reduced pressure. The crude

mixture of **SI16**/**SI17** (0.801 g, 2.37 mmol, 92%) was used for the oxidation without further purification.

The reaction was carried out in moisture-free glassware under inert atmosphere. To a solution of $(COCl)_2$ (0.307 mL, 3.56 mmol, 1.50 eq.) in anhydrous DCM (20 mL) anhydrous DMSO (0.533 mL, 7.50 mmol, 3.16 eq.) was added carefully at −78 °C. After stirring for 15 min, the crude mixture of **SI16**/**SI17** (0.801 g, 2.37 mmol, 1.00 eq.) dissolved in anhydrous DCM (20 mL) was added. The reaction mixture was stirred for 1.5 h at −78 °C. Then DIPEA (2.30 mL, 13.1 mmol, 5.50 eq.) was added and the solution was stirred for 30 min at −78 °C and for further 30 min without cooling bath. A mixture of cooled toluene/saturated aqueous NH_4Cl (4:1, 250 mL, pre-cooled to 0 °C) was added to the reaction mixture. The layers were separated and the organic layer was washed with saturated aqueous NH_4Cl (3 x 50 mL, pre-cooled to 0 °C), with saturated aqueous $NaHCO_3$ (50 mL, pre-cooled to 0 °C), and with saturated aqueous NaCl (50 mL, pre-cooled to 0 °C). The organic layer was dried over $MgSO_4$, filtered, and concentrated under reduced pressure to yield crude ketone **SI18** (0.854 g, max. 2.37 mmol, quant., R_f (*n*-heptane/ethyl acetate 1:3): 0.64) as slightly yellow oil which was used in the next stage without further purification.

The reaction was carried out in moisture-free glassware under inert atmosphere. To a solution of ketone **SI18** (0.854 g, max. 2.37 mmol, 1.00 eq.) in anhydrous THF (30 mL) was added EtMgBr (1 M in THF, 9.49 mL, 9.49 mmol, 4.00 eq.) at room temperature. After 90 min, TLC indicated full conversion of the starting material (R_f of **SI19** (*n*-heptane/ethyl acetate 1:3): 0.61). Saturated aqueous NH_4Cl (20 mL) and EtOAc (100 mL) were added to the reaction mixture, the layers were separated and the organic layer was washed with saturated aqueous NaCl (20 mL). The organic layer was dried over $MgSO_4$, filtered, and concentrated under reduced pressure. The crude product of **SI19** was dissolved in 1,4-dioxane (20 mL) and aqueous HCl (1 N, 1 mL) and the reaction mixture was stirred for 30 min until full hydrolysis of the orthoester was observed by TLC. All volatiles were removed under reduced pressure and the residue was purified *via* HPLC (RP-18, 15 min, 30-40% MeCN in H_2O/TFA 0.1%) to yield **SI20** (0.126 g, 0.329 mmol, 13% over four steps)[11] as a colorless oil.

---

[11] 14:1 *dr* and >10:1 *dr* were determined for **SI20**:**SI27** by chirale HPLC and ^{1}H-NMR analysis.

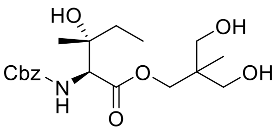

SI20

^{1}**H-NMR** (DMSO-d$_6$, 400 MHz): 7.42–7.26 (m, 5H, aryl-*H*), 7.21 (d, 1H, *J* = 8.8 Hz, N*H*), 5.04 (s, 2H, Ph-CH_2), 4.07 (d, 1H, *J* = 8.8 Hz, NH-C*H*), ~4.06 (s, v. br, 3H, O*H*), 3.90 (s, 2H, CH_2), 3.33–3.21 (m, 4H, CH_2-OH), 1.48 (m, 2H, CH_2-CH$_3$), 1.11 (s, 3H, CH_3), 0.85–0.76 (m, 6H, CH$_2$-CH_3, CH_3); ^{13}**C-NMR** (DMSO-d$_6$, 100 MHz): 170.8 (*C*O), 156.1 (*C*O-NH), 136.8 (aryl-C_q), 128.3 (aryl-*C*), 127.8 (aryl-*C*), 127.7 (aryl-*C*), 72.4 (C_q-OH), 67.0 (*C*H$_2$), 65.7 (Ph-*C*H$_2$), 63.5/63.5 (*C*H$_2$-OH), 61.7 (*C*H-NH), 40.5 (C_q-CH$_3$), 31.9 (*C*H$_2$-CH$_3$), 23.1 (*C*H$_3$), 16.5 (*C*H$_3$), 8.0 (CH$_2$-*C*H$_3$); **HRMS (ESI)** m/z calcd. for $C_{19}H_{29}NO_7Na$: $(M+Na)^+$, 406.1836; found: 406.1837 $(M+Na)^+$; **Chiral HPLC** (Chiralpak IF/181, 250x4.6 mm; *n*-heptane:EtOH:MeOH 5:1:1 + 0.1% TFA): **SI20** (Rt = 13.2 min) : ***ent*-SI20** (Rt = 8.2 min) 89.5 : 3.9 (92% *ee*); **Specific rotation** $[\alpha]_D^{20.0}$ = − 21.1 (c = 0.07; MeCN).

***Rac*-SI20** (0.300 g, 0.783 mmol, 37% over four steps)[12] was synthesized from ***rac*-SI7** (0.685 g, 2.13 mmol, 1.00 eq.) in analogous manner. In the conversion of ***rac*-SI18** to ***rac*-Si19**, an additional portion of EtMgBr (1 M in THF, 1.00 eq.) was added after 90 min and the reaction mixture was stirred for 60 min after addition. This modification resulted in a significantly improved overall yield. The NMR data are identical to the ones reported for **SI20**.

For ***rac*-SI20**: **Chiral HPLC** (Chiralpak IF/181, 250x4.6 mm; *n*-heptane:EtOH:MeOH 5:1:1 + 0.1% TFA): **SI20** (Rt = 13.5 min) : ***ent*-SI20** (Rt = 8.2 min) 47.9 : 44.6 (4% *ee*).

3-Hydroxy-2-(hydroxymethyl)-2-methylpropyl (2*S*,3*S*)-2-(((benzyloxy)carbonyl)amino)-3-hydroxy-3-methylpenta-noate (SI27):

The reaction was carried out in moisture-free glassware under inert atmosphere. To a solution of aldehyde **SI7** (0.830 g, 2.58 mmol, 1.00 eq.) in anhydrous THF (30 mL) was added EtMgBr (1 M in THF, 10.3 mL, 10.3 mmol, 4.00 eq.) at room temperature. After 90 min, TLC indicated full conversion of the starting material (R_f of **SI23**/**SI24** (*n*-heptane/ethyl acetate 1:3): 0.58). Saturated aqueous NH_4Cl (20 mL) and EtOAc (100 mL) were added to the reaction mixture, the layers were separated and the organic layer was washed with saturated aqueous NaCl (20 mL). The organic layer was dried over $MgSO_4$, filtered, and concentrated under reduced pressure. The crude

[12] 12:1 *dr* and >10:1 *dr* were determined for **SI20**:**SI27** by chirale HPLC and ^{1}H-NMR analysis.

mixture of **SI23**/**SI24** (0.813 g, 2.31 mmol, 90%) was used for the oxidation without further purification.

The reaction was carried out in moisture-free glassware under inert atmosphere. To a solution of $(COCl)_2$ (0.300 mL, 3.47 mmol, 1.50 eq.) in anhydrous DCM (20 mL) anhydrous DMSO (0.509 mL, 7.17 mmol, 3.10 eq.) was added carefully at −78 °C. After stirring for 15 min, the crude mixture of **SI23**/**SI24** (0.813 g, 2.31 mmol, 1.00 eq.) dissolved in anhydrous DCM (20 mL) was added. The reaction mixture was stirred for 1.5 h at −78 °C. Then DIPEA (2.24 mL, 12.7 mmol, 5.50 eq.) was added and the solution was stirred for 30 min at −78 °C and for further 30 min without cooling bath. A mixture of cooled toluene/saturated aqueous NH_4Cl (4:1, 250 mL, pre-cooled to 0 °C) was added to the reaction mixture. The layers were separated and the organic layer was washed with saturated aqueous NH_4Cl (3 x 50 mL, pre-cooled to 0 °C), with saturated aqueous $NaHCO_3$ (50 mL, pre-cooled to 0 °C), and with saturated aqueous NaCl (50 mL, pre-cooled to 0 °C). The organic layer was dried over $MgSO_4$, filtered, and concentrated under reduced pressure to yield crude ketone **SI25** (0.808 g, 2.31 mmol, quant., R_f (*n*-heptane/ethyl acetate 1:3): 0.68) as slightly yellow oil which was used in the next stage without further purification.

The reaction was carried out in moisture-free glassware under inert atmosphere. To a solution of ketone **SI25** (0.808 g, max. 2.31 mmol, 1.00 eq.) in anhydrous THF (30 mL) was added MeMgBr (3 M in Et_2O, 3.08 mL, 9.25 mmol, 4.00 eq.) at room temperature. After 90 min, TLC indicated full conversion of the starting material (R_f of **SI26** (*n*-heptane/ethyl acetate 1:3): 0.61). Saturated aqueous NH_4Cl (20 mL) and EtOAc (100 mL) were added to the reaction mixture, the layers were separated and the organic layer was washed with saturated aqueous NaCl (20 mL). The organic layer was dried over $MgSO_4$, filtered, and concentrated under reduced pressure. The crude product of **SI26** was dissolved in 1,4-dioxane (20 mL) and aqueous HCl (1 N, 1 mL) and the reaction mixture was stirred for 30 min until full hydrolysis of the orthoester was observed by TLC. All volatiles were removed under reduced pressure and the residue was purified *via* HPLC (RP-18, 15 min, 30-40% MeCN in H_2O/TFA 0.1%) to yield **SI27** (0.218 g, 0.569 mmol, 22% over four steps).[13] as a colorless oil.

[13] 32:1 *dr* and >10:1 *dr* were determined for **SI27**:**SI20** by chirale HPLC and ^{1}H-NMR analysis.

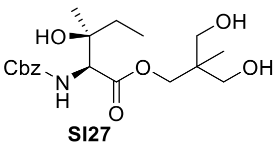
SI27

^{1}H-NMR (DMSO-d$_6$, 400 MHz): 7.42–7.26 (m, 6H, aryl-*H*, N*H*), 5.04 (s, 2H, Ph-CH_2), 4.54 (s, 1H, C$_q$-O*H*), 4.45 (t, 2H, J = 5.2 Hz, CH$_2$-O*H*), 4.10 (d, 1H, J = 9.2 Hz, NH-C*H*), 3.93 (d, 1H, J = 10.7 Hz, CH_2), 3.87 (d, 1H, J = 10.7 Hz, CH_2), 3.31–3.21 (m, 4H, CH_2-OH), 1.56–1.39 (m, 2H, CH_2-CH$_3$), 0.86 (s, 3H, CH_3), 0.84 (t, 3H, J = 7.4 Hz, CH$_2$-CH_3), 0.80 (s, 3H, CH_3); **^{13}C-NMR** (DMSO-d$_6$, 100 MHz): 170.8 (*C*O), 156.1 (*C*O-NH), 136.9 (aryl-C_q), 128.4 (aryl-*C*), 127.9 (aryl-*C*), 127.7 (aryl-*C*), 72.7 (C_q-OH), 67.0 (*C*H$_2$), 65.6 (Ph-*C*H$_2$), 63.6 (*C*H$_2$-OH), 61.6 (*C*H-NH), 40.5 (C_q-CH$_3$), 31.6 (*C*H$_2$-CH$_3$), 23.1 (*C*H$_3$), 16.5 (*C*H$_3$), 7.9 (CH$_2$-*C*H$_3$); **HRMS (ESI)** m/z calcd. for $C_{19}H_{29}NO_7Na$: $(M+Na)^+$, 406.1836; found: 406.1839 $(M+Na)^+$; **Chiral HPLC** (Chiralpak IF/181, 250x4.6 mm; *n*-heptane:EtOH:MeOH 5:1:1 + 0.1% TFA): **SI27** (Rt = 22.9 min) : ***ent*-SI27** (Rt = 6.9 min) 91.7 :5.2 (89% *ee*); **Specific rotation** $[\alpha]_D^{20.0}$ = − 11.6 (c = 0.35; MeCN).

***Rac*-SI27** (0.337 g, 0.879 mmol, 41% over four steps)[14] was synthesized from ***rac*-SI7** (0.685 g, 2.13 mmol, 1.00 eq.) in analogous manner. In the conversion of ***rac*-SI25** to ***rac*-Si26**, an additional portion of MeMgBr (3 M in Et$_2$O, 1.00 eq.) was added after 90 min and the reaction mixture was stirred for 60 min after addition. This modification resulted in a significantly improved overall yield. The NMR data are identical to the ones reported for **SI27**.

For ***rac*-SI27**: **Chiral HPLC** (Chiralpak IF/181, 250x4.6 mm; *n*-heptane:EtOH:MeOH 5:1:1 + 0.1% TFA): **SI27** (Rt = 23.0 min) : ***ent*-SI27** (Rt = 6.9 min) 51.4 :45.7 (6% *ee*).

(2*S*,3*R*)-2-(((Benzyloxy)carbonyl)amino)-3-hydroxy-3-methylpentanoic acid (SI21):

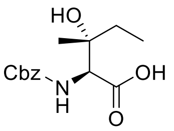
SI21

To a solution of **SI20** (0.084 g, 0.22 mmol, 1.00 eq.) in MeCN/H$_2$O (4:1, 3.2 mL/0.8 mL) was added LiOH (1 N in H$_2$O, 0.22 mL, 0.22 mmol, 1.00 eq.) at room temperature. After 20 min, another portion of LiOH (1 N in H$_2$O, 0.11 mL, 0.11 mmol, 0.50 eq.) was added to reach full conversion of starting material after 30 min monitored by LC-MS. The reaction was stopped by addition of acetic acid (0.1 mL). All volatiles were removed under reduced pressure

[14] 33:1 *dr* and >10:1 *dr* were determined for **SI27**:**SI20** by chirale HPLC and ^{1}H-NMR analysis.

and the residue was purified *via* HPLC (RP-18, 15 min, 5-55% MeCN in H_2O/TFA 0.1%) to yield **SI21** (0.021 g, 0.075 mmol, 34%)[15] as a colorless oil.[16]

^{1}H-NMR (DMSO-d$_6$, 400 MHz): 7.42–7.26 (m, 5H, aryl-*H*), 7.03 (d, 1H, *J* = 8.8 Hz, N*H*), 5.04 (s, 2H, Ph-C*H*$_2$), 3.96 (d, 1H, *J* = 9.0 Hz, NH-C*H*), 1.56–1.34 (m, 2H, C*H*$_2$-CH$_3$), 1.11 (s, 3H, C*H*$_3$), 0.80 (t, 3H, *J* = 7.5 Hz, CH$_2$-C*H*$_3$); **^{13}C-NMR** (DMSO-d$_6$, 100 MHz): 172.3 (*C*OOH), 156.2 (*C*O-NH), 137.0 (aryl-*C*$_q$), 128.4 (aryl-*C*), 127.8 (aryl-*C*), 127.7 (aryl-*C*), 72.4 (*C*$_q$-OH), 65.6 (Ph-*C*H$_2$), 61.4 (*C*H-NH), 32.0 (*C*H$_2$-CH$_3$), 23.1 (*C*H$_3$), 8.0 (CH$_2$-*C*H$_3$); **HRMS (ESI)** m/z calcd. for $C_{14}H_{19}NO_5Na$: $(M+Na)^+$, 304.1155; found: 304.1154 $(M+Na)^+$; **Chiral HPLC** (Chiralpak IF/181, 250x4.6 mm; *n*-heptane:EtOH 10:1 + 0.1% TFA): **SI21** (Rt = 16.7 min) : ***ent*-SI21** (Rt = 29.3 min) 75.2 : 2.6 (93% *ee*); **Specific rotation** $[\alpha]_D^{19.1}$ = 0 (c = 0.19; MeCN).[17]

***Rac*-SI21** (0.072 g, 0.26 mmol, 38%)[18] and ***rac*-SI22** (0.038 g, 0.22 mmol, 32%) were synthesized from ***rac*-SI20** (0.260 g, 0.678 mmol, 1.00 eq.) in analogous manner. The NMR data of ***rac*-SI21** are identical to the ones reported for **SI21**. ***Rac*-SI21** was obtained as colorless oil and ***rac*-SI22** as colorless solid.

For ***rac*-SI21**: **Chiral HPLC** (Chiralpak IF/181, 250x4.6 mm; *n*-heptane:EtOH 10:1 + 0.1% TFA): **SI21** (Rt = 16.7 min) : ***ent*-SI21** (Rt = 28.9 min) 43.0 : 43.0 (0% *ee*).

(4*S*,5*R*)-5-Ethyl-5-methyl-2-oxooxazolidine-4-carboxylic acid (ent-SI22):

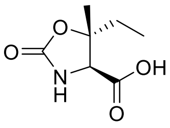

***rac*-SI22**

^{1}H-NMR (DMSO-d$_6$, 400 MHz): 13.23 (s, br, 1H, COO*H*), 7.89 (s, 1H, N*H*), 4.03 (d, 1H, *J* = 0.6 Hz, C*H*), 1.72 (q, 2H, *J* = 7.5 Hz, C*H*$_2$), 1.24 (s, 3H, C*H*$_3$), 0.91 (t, 3H, *J* = 7.5 Hz, CH$_2$-C*H*$_3$); **^{13}C-NMR** (DMSO-d$_6$, 100 MHz): 171.3 (*C*OOH), 157.4 (*C*O-NH), 82.7 (O-*C*$_q$), 61.2 (*C*H-NH), 33.2 (*C*H$_2$-CH$_3$), 20.7 (*C*H$_3$), 7.5 (CH$_2$-*C*H$_3$); **HRMS (ESI)** m/z calcd. for $C_7H_{12}NO_4$: $(M+H)^+$, 174.0761; found: 174.0761 $(M+H)^+$.

(2*S*,3*S*)-2-(((Benzyloxy)carbonyl)amino)-3-hydroxy-3-methylpentanoic acid (SI28):

[15] 3.5:1 *dr* and 3:1 *dr* were determined for **SI21**:**SI28** by chirale HPLC and ^{1}H-NMR analysis.
[16] The formation of **SI22** was observed *via* LC-MS, but was not isolated for the *S*-series.
[17] Since the sample is a mixture of diastereomers and the measured rotation was close to 0, no specific rotation was determined.
[18] 6:1 *dr* and >5:1 *dr* were determined for **SI21**:**SI28** by chirale HPLC and ^{1}H-NMR analysis.

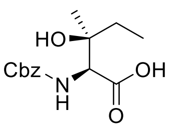

SI28

To a solution of **SI27** (0.178 g, 0.464 mmol, 1.00 eq.) in MeCN/H_2O (4:1, 7.2 mL/1.8 mL) was added LiOH (1 N in H_2O, 0.46 mL, 0.46 mmol, 1.00 eq.) at room temperature. After 20 min, another portion of LiOH (1 N in H_2O, 0.23 mL, 0.23 mmol, 0.50 eq.) was added to reach full conversion of starting material after 30 min monitored by LC-MS. The reaction was stopped by addition of acetic acid (0.1 mL). All volatiles were removed under reduced pressure and the residue was purified *via* HPLC (RP-18, 15 min,10-50% MeCN in H_2O/TFA 0.1%) to yield **SI28** (0.051 g, 0.18 mmol, 39%)[19] as a colorless oil.[20]

^{1}H-NMR (DMSO-d_6, 400 MHz): 7.42–7.27 (m, 5H, aryl-*H*), 7.18 (d, 1H, J = 9.2 Hz, N*H*), 5.05 (s, 2H, Ph-CH_2), 4.01 (d, 1H, J = 9.2 Hz, NH-C*H*), 1.58–1.40 (m, 2H, CH_2-CH_3), 1.09 (s, 3H, CH_3), 0.84 (t, 3H, J = 7.4 Hz, CH_2-CH_3); **^{13}C-NMR** (DMSO-d_6, 100 MHz): 172.3 (*C*OOH), 156.1 (*C*O-NH), 137.0 (aryl-C_q), 128.3 (aryl-*C*), 127.8 (aryl-*C*), 127.7 (aryl-*C*), 72.6 (C_q-OH), 65.5 (Ph-CH_2), 61.2 (*C*H-NH), 31.5 (CH_2-CH_3), 23.1 (CH_3), 7.8 (CH_2-CH_3); **HRMS (ESI)** m/z calcd. for $C_{14}H_{19}NO_5Na$: $(M+Na)^+$, 304.1155; found: 304.1157 $(M+Na)^+$; **Chiral HPLC** (Chiralpak IF/181, 250x4.6 mm; *n*-heptane:EtOH 10:1 + 0.1% TFA): **SI28** (Rt = 20.6 min) : ***ent*-SI28** (Rt = 27.2 min) 94.2 : 5.1 (90% *ee*); **Specific rotation** $[\alpha]_D^{19.9}$ = + 18.5 (c = 0.05; MeCN).

(4*S*,5*S*)-5-Ethyl-5-methyl-2-oxooxazolidine-4-carboxylic acid (SI29):

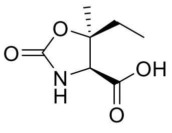

rac-SI29

^{1}H-NMR (DMSO-d_6, 400 MHz): 13.24 (s, br, 1H, COO*H*), 7.88 (s, 1H, N*H*), 4.05 (d, 1H, J = 0.6 Hz, C*H*), 1.73 (q, 2H, J = 7.5 Hz, CH_2), 1.24 (s, 3H, CH_3), 0.91 (t, 3H, J = 7.5 Hz, CH_2-CH_3); **^{13}C-NMR** (DMSO-d_6, 100 MHz): 171.0 (*C*OOH), 157.4 (*C*O-NH), 82.7 (O-C_q), 63.4 (*C*H-NH), 28.3 (CH_2-CH_3), 24.3 (CH_3), 7.7 (CH_2-CH_3); **HRMS (ESI)** m/z calcd. for $C_7H_{12}NO_4$: $(M+H)^+$, 174.0761; found: 174.0762 $(M+H)^+$.

***Rac*-SI28** (0.132 g, 0.469 mmol, 61%)[21] and ***rac*-SI29** (0.021 g, 0.12 mmol, 16%) were synthesized from ***rac*-SI27** (0.297 g, 0.775 mmol, 1.00 eq.) in analogous manner. The NMR data of ***rac*-SI28** are identical to the ones reported for **SI28**. ***Rac*-SI28** was obtained as colorless oil and ***rac*-SI29** as colorless solid.

[19] 138:1 *dr* and >100:1 *dr* were determined for **SI28**:**SI21** by chirale HPLC and ^{1}H-NMR analysis.
[20] The formation of **SI29** was observed *via* LC-MS, but was not isolated for the *S*-series.
[21] 33:1 *dr* and >50:1 *dr* were determined for **SI28**:**SI21** by chirale HPLC and ^{1}H-NMR analysis.

For ***rac*-SI28**: **Chiral HPLC** (Chiralpak IF/181, 250x4.6 mm; *n*-heptane:EtOH 10:1 + 0.1% TFA): **SI28** (Rt = 20.8 min) : ***ent*-SI28** (Rt = 26.8 min) 48.4 : 48.7 (0% *ee*).

4.2. NMR spectra of synthetic β-hydroxyamino acid intermediates

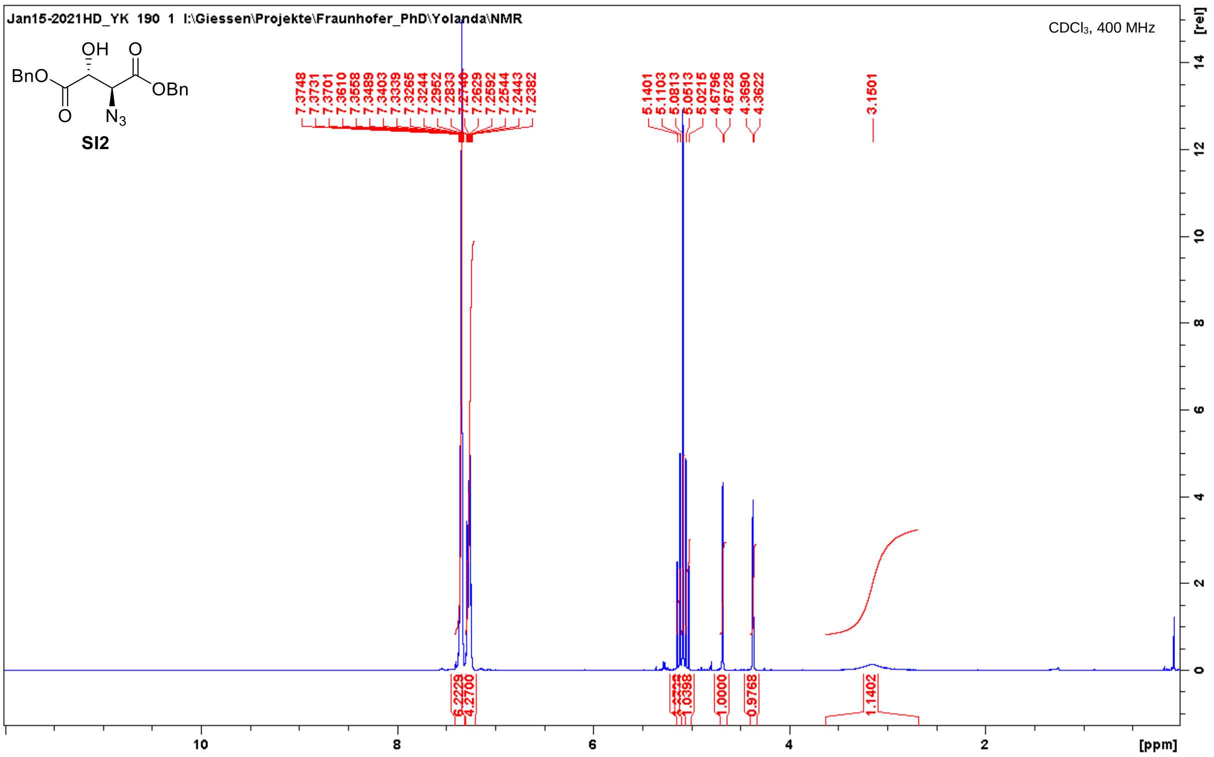

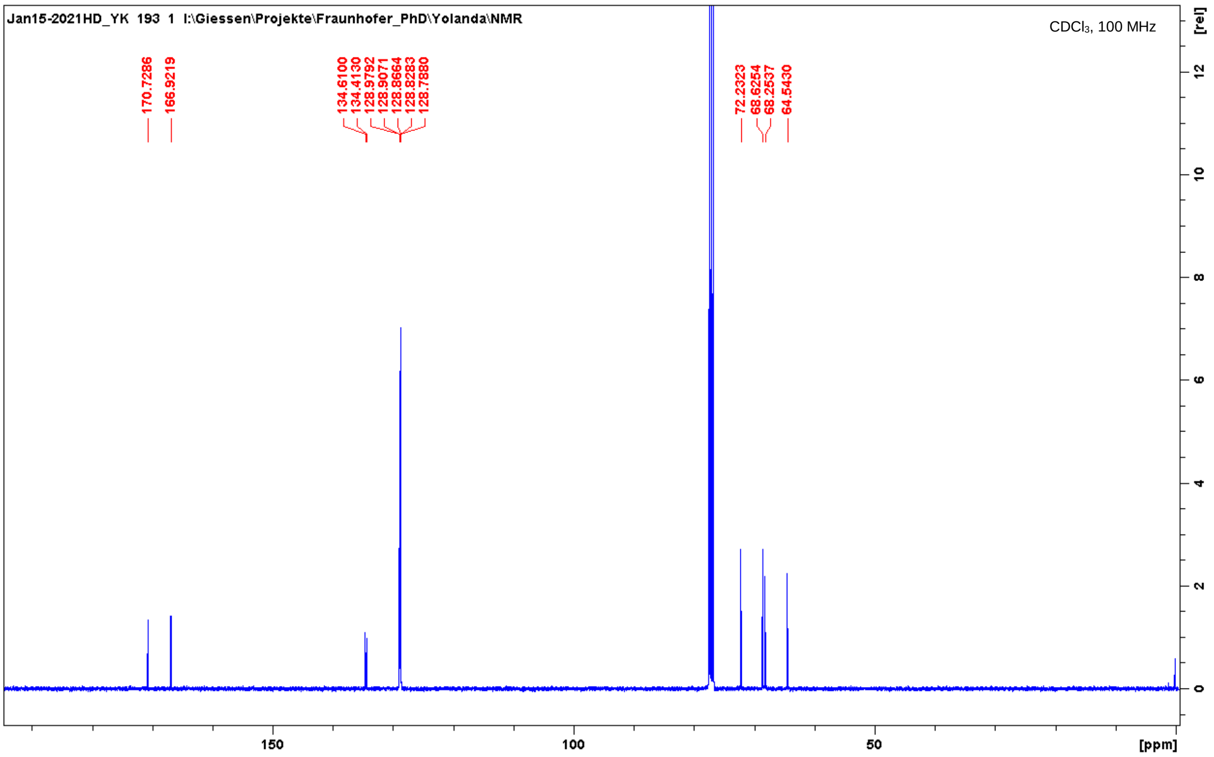

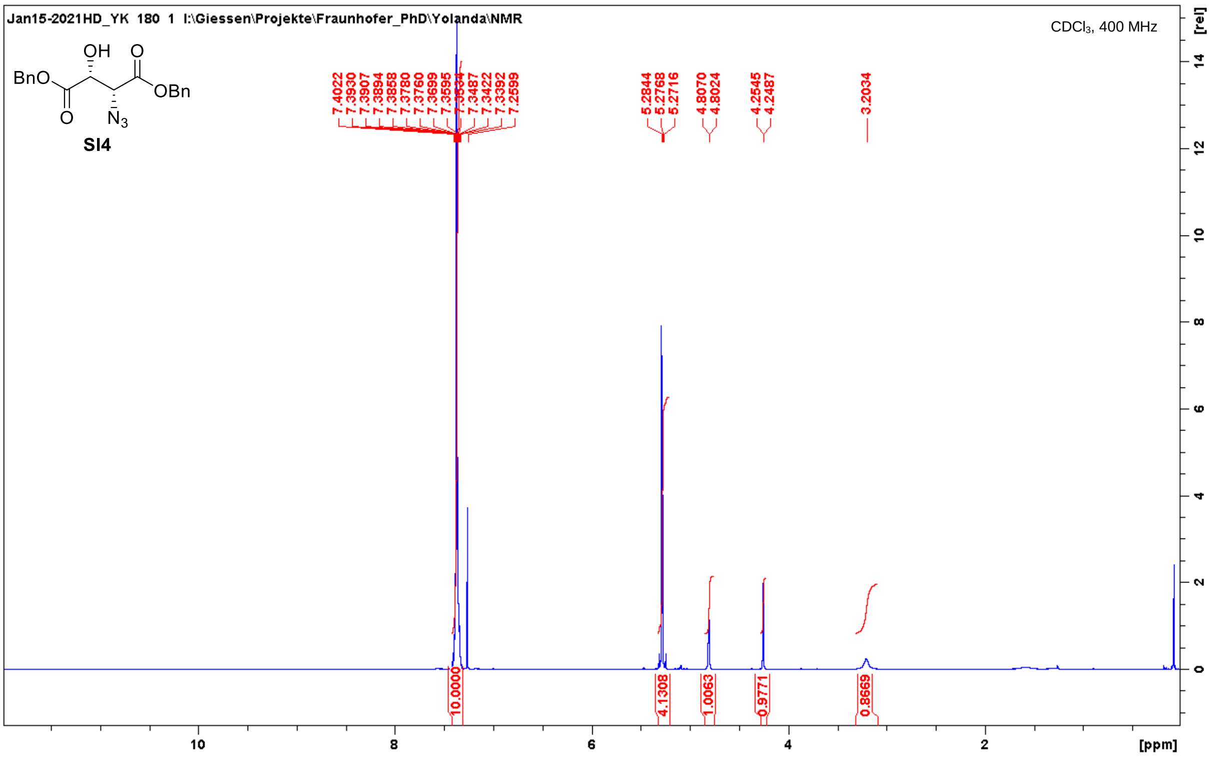
Jan15-2021HD_YK 180 1 I:\Giessen\Projekte\Fraunhofer_PhD\Yolanda\NMR
CDCl3, 400 MHz
BnO
OH
O
OBn
O
N3
SI4
7.4022
7.3930
7.3907
7.3894
7.3858
7.3780
7.3760
7.3699
7.3595
7.3487
7.3422
7.3392
7.2599
5.2844
5.2768
5.2716
4.8070
4.8024
4.2545
4.2487
3.2034
10.0000
4.1308
1.0063
0.9771
0.8669
[ppm]
[rel]

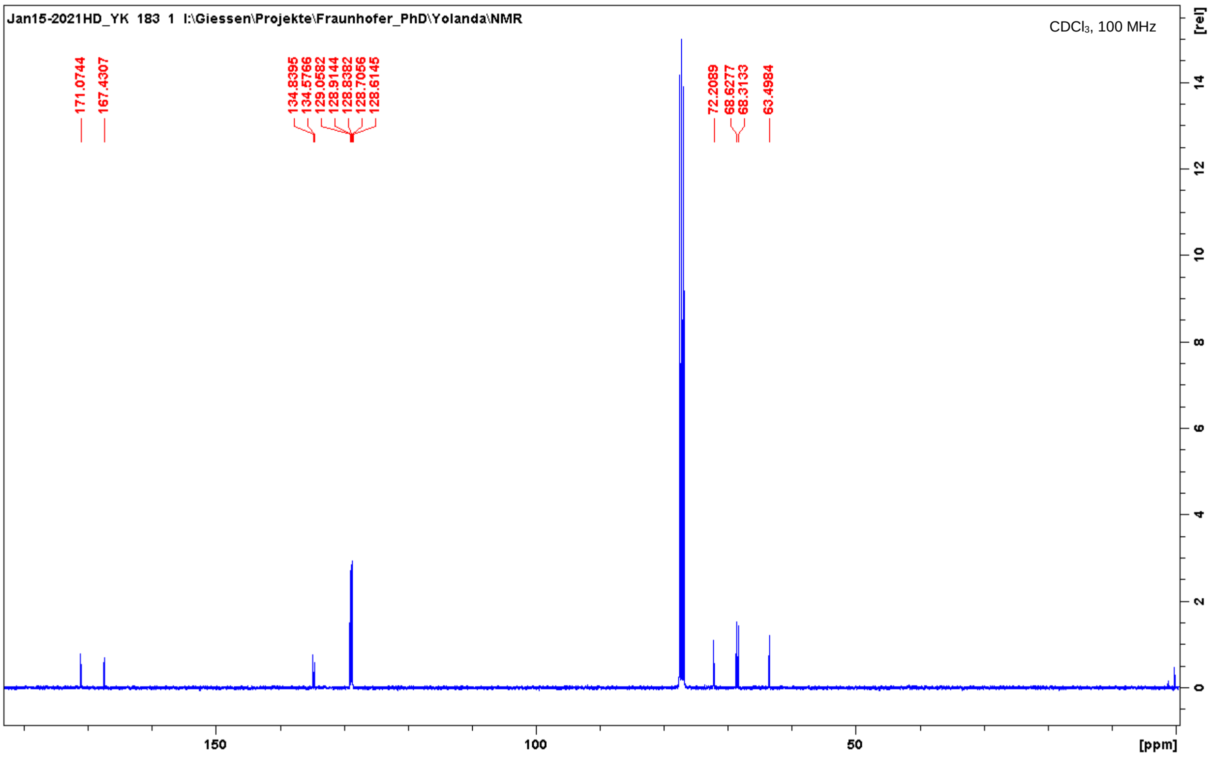
Jan15-2021HD_YK 183 1 I:\Giessen\Projekte\Fraunhofer_PhD\Yolanda\NMR
CDCl3, 100 MHz
171.0744
167.4307
134.8395
134.5786
129.0582
128.9144
128.8382
128.7056
128.6145
72.2089
68.6277
68.3133
63.4984
[ppm]
[rel]

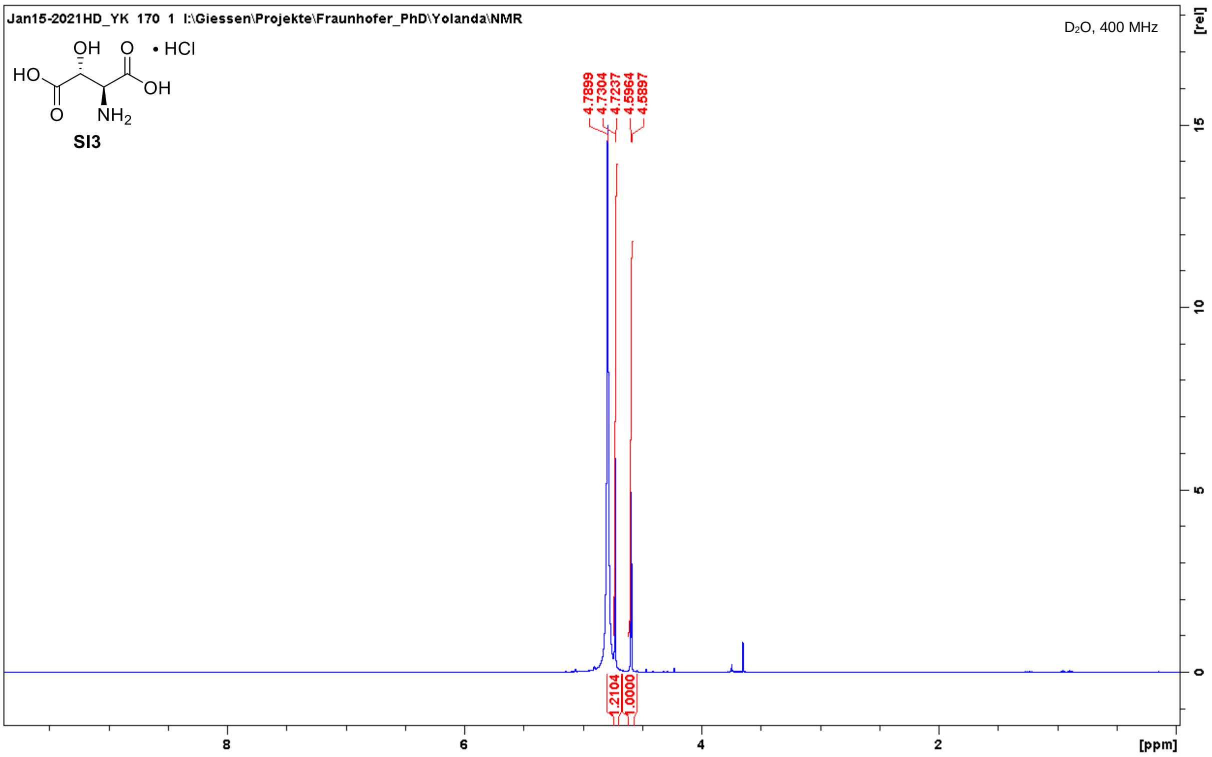
Jan15-2021HD_YK 170 1 I:\Giessen\Projekte\Fraunhofer_PhD\Yolanda\NMR
D2O, 400 MHz
SI3
4.7899
4.7304
4.7237
4.5964
4.5897
1.2104
1.0000
[ppm]
[rel]

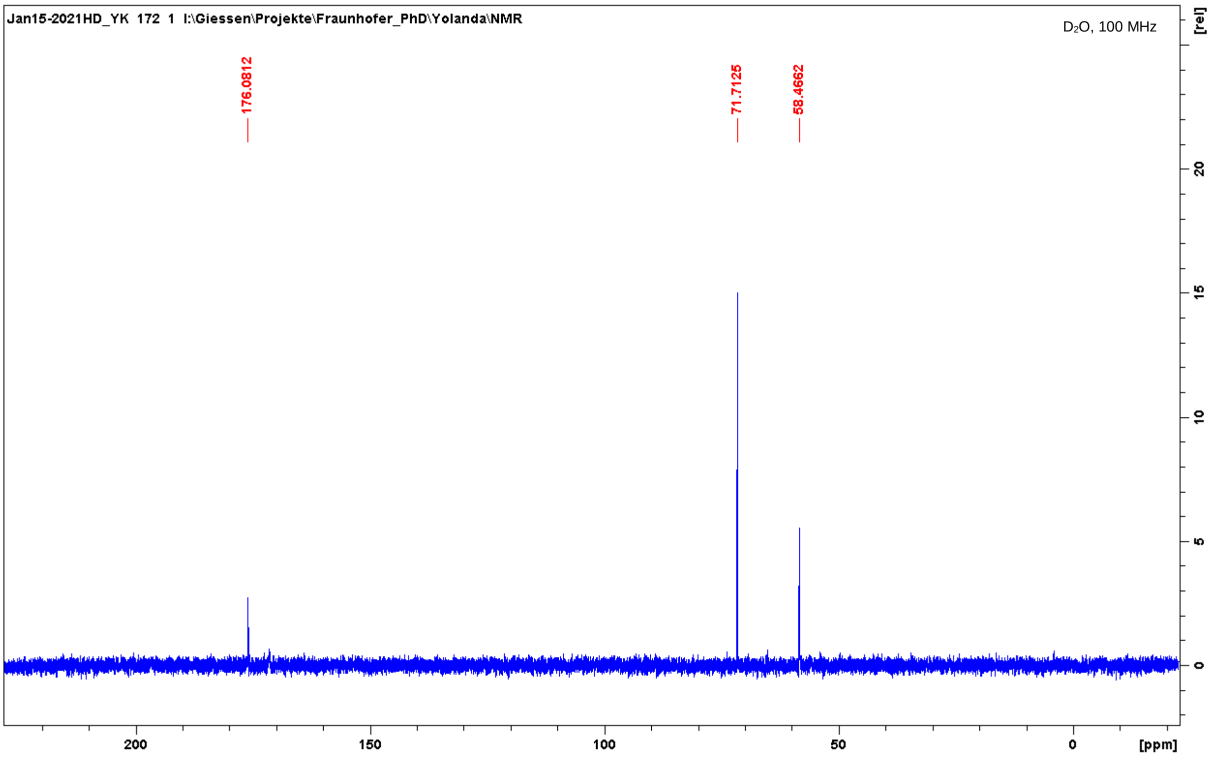
Jan15-2021HD_YK 172 1 I:\Giessen\Projekte\Fraunhofer_PhD\Yolanda\NMR
D2O, 100 MHz
176.0812
71.7125
58.4662
[ppm]
[rel]

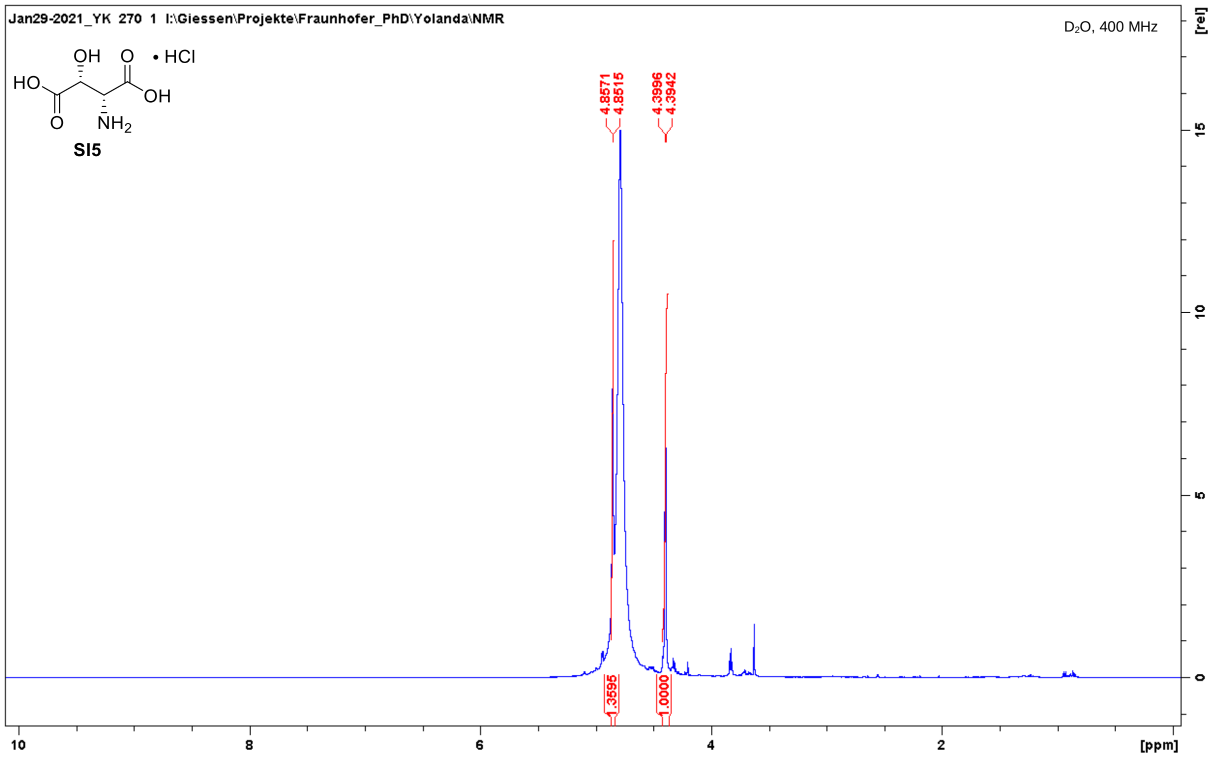
Jan29-2021_YK 270 1 I:\Giessen\Projekte\Fraunhofer_PhD\Yolanda\NMR
D₂O, 400 MHz
• HCl
SI5
4.8571
4.8515
4.3996
4.3942
1.3595
1.0000
[ppm]
[rel]

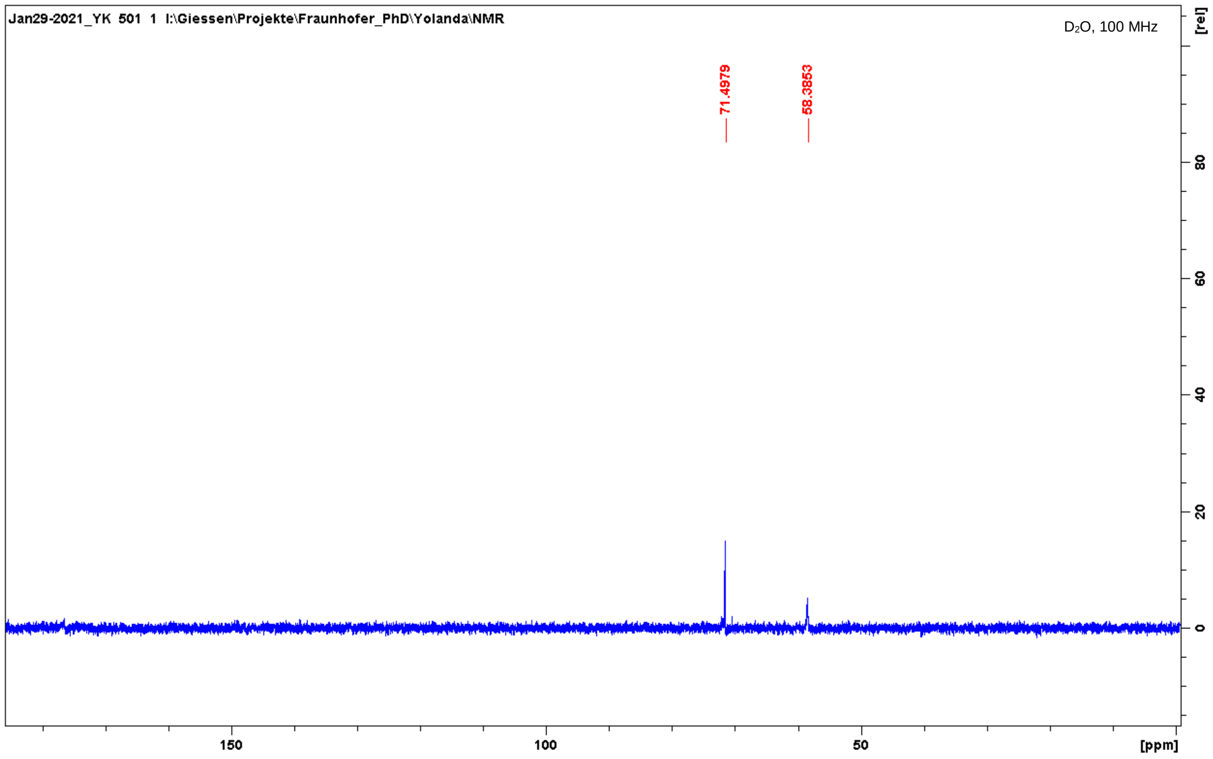
Jan29-2021_YK 501 1 I:\Giessen\Projekte\Fraunhofer_PhD\Yolanda\NMR
D₂O, 100 MHz
71.4979
58.3853
[ppm]
[rel]

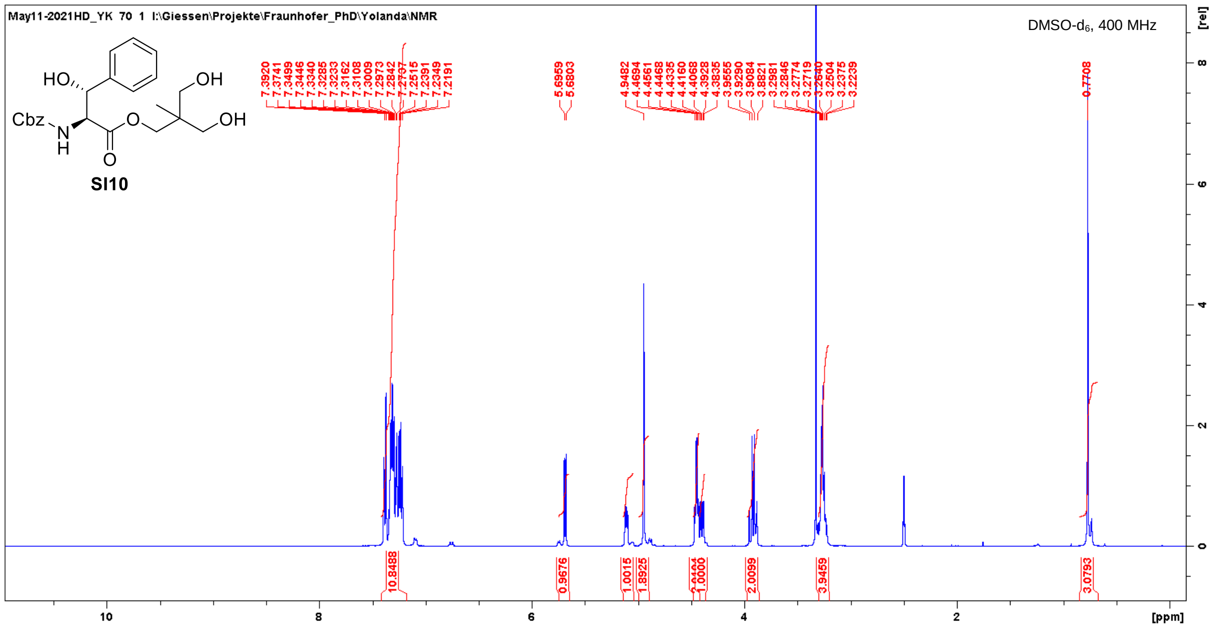
May11-2021HD_YK 70 1 I:\Giessen\Projekte\Fraunhofer_PhD\Yolanda\NMR
DMSO-d6, 400 MHz
HO
OH
Cbz
N
H
O
O
OH
SI10
7.3920 7.3741 7.3499 7.3446 7.3340 7.3285 7.3233 7.3162 7.3108 7.3009 7.2973 7.2842 7.2707 7.2515 7.2391 7.2349 7.2191
5.6959 5.6803
4.9482 4.4694 4.4561 4.4468 4.4335 4.4160 4.4068 4.3928 4.3835 3.9555 3.9290 3.9084 3.8821 3.2981 3.2846 3.2774 3.2719 3.2640 3.2504 3.2375 3.2239
0.7708
10.8488
0.9676
1.0015
1.8925
1.0000
1.0000
2.0099
3.9459
3.0793
[ppm]
[rel]

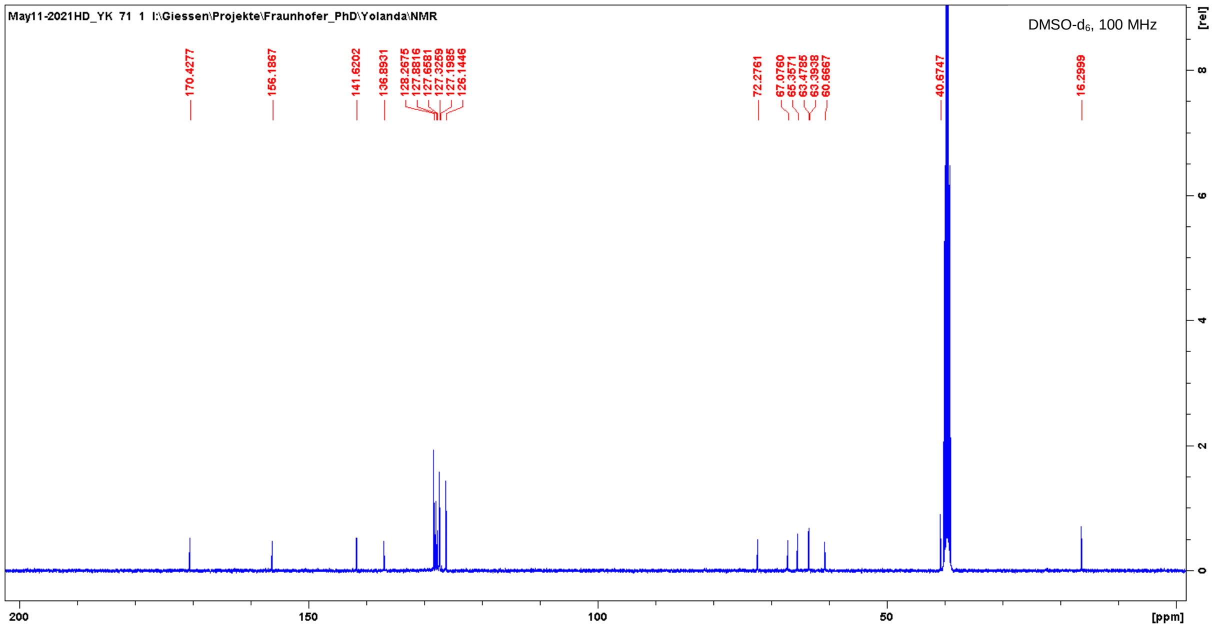
May11-2021HD_YK 71 1 I:\Giessen\Projekte\Fraunhofer_PhD\Yolanda\NMR
DMSO-d6, 100 MHz
170.4277
156.1867
141.6202
136.8931
128.2675 127.8816 127.6581 127.3259 127.1985 126.1446
72.2761
67.0760 65.3571 63.4785 63.3938 60.6667
40.6747
16.2999
[ppm]
[rel]

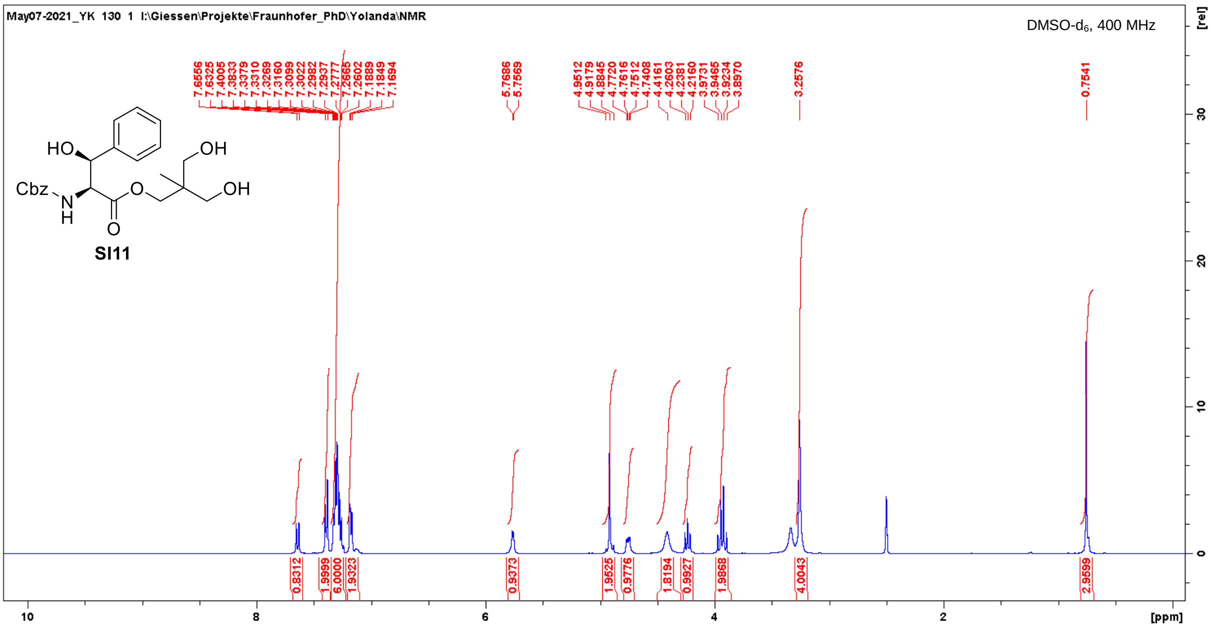
May07-2021_YK 130 1 I:\Giessen\Projekte\Fraunhofer_PhD\Yolanda\NMR
DMSO-d6, 400 MHz
SI11
[ppm]
[rel]

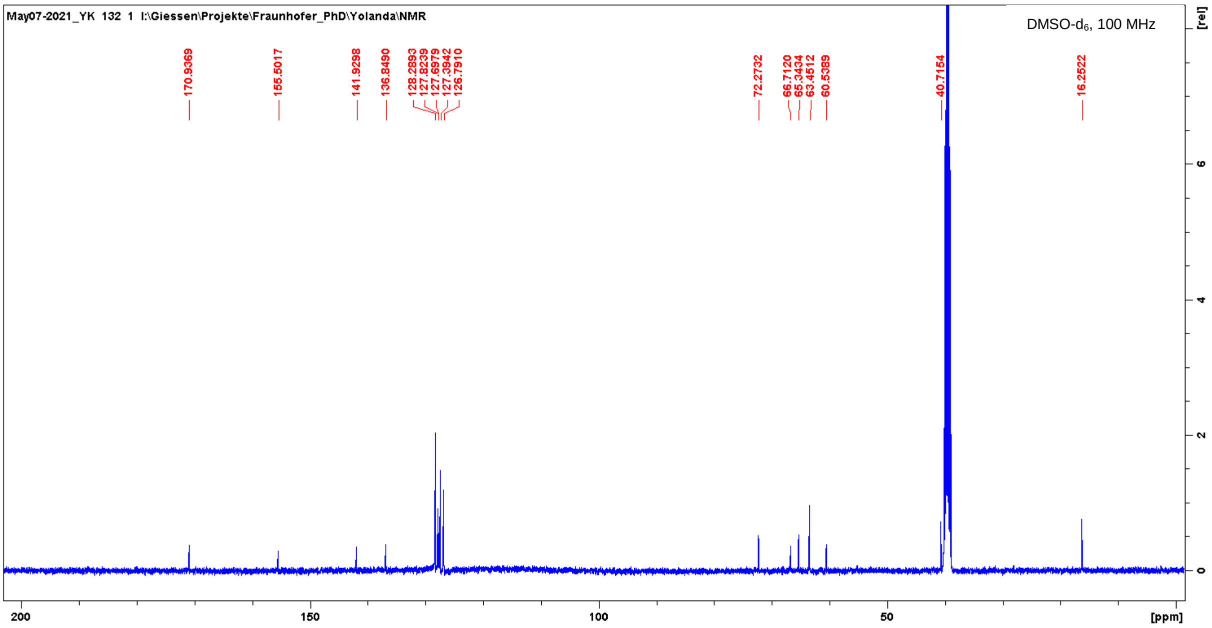
May07-2021_YK 132 1 I:\Giessen\Projekte\Fraunhofer_PhD\Yolanda\NMR
DMSO-d6, 100 MHz
170.9369
155.5017
141.9298
136.8490
72.2732
40.7154
16.2522
[ppm]
[rel]

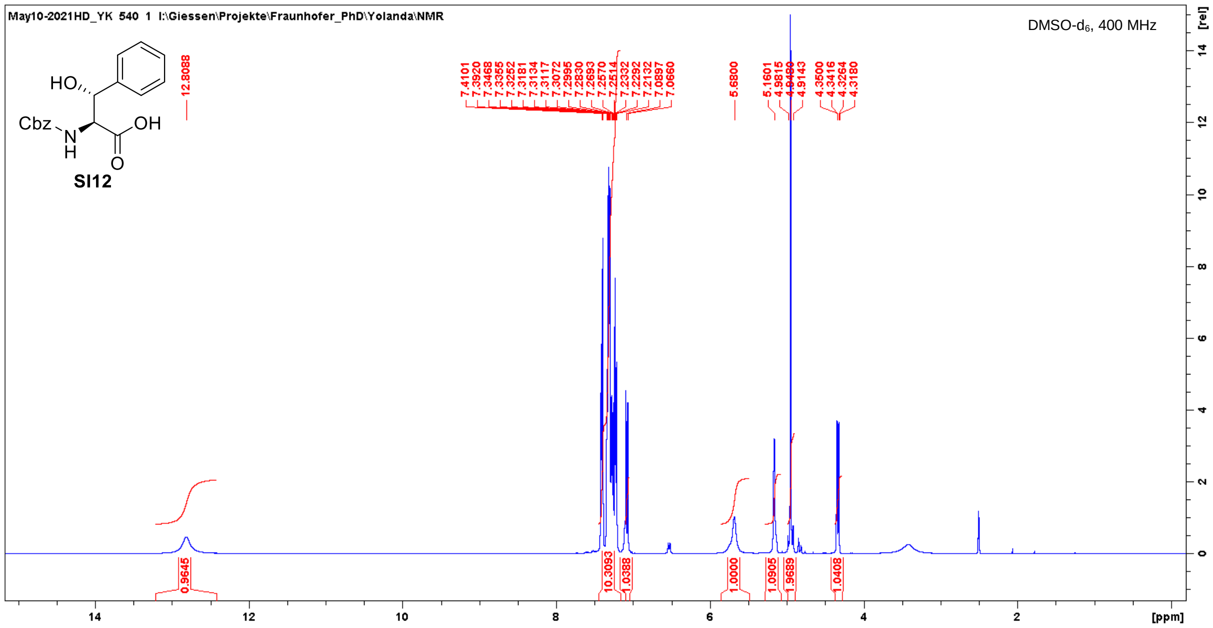
May10-2021HD_YK 540 1 I:\Giessen\Projekte\Fraunhofer_PhD\Yolanda\NMR
DMSO-d6, 400 MHz
SI12
[ppm]
[rel]

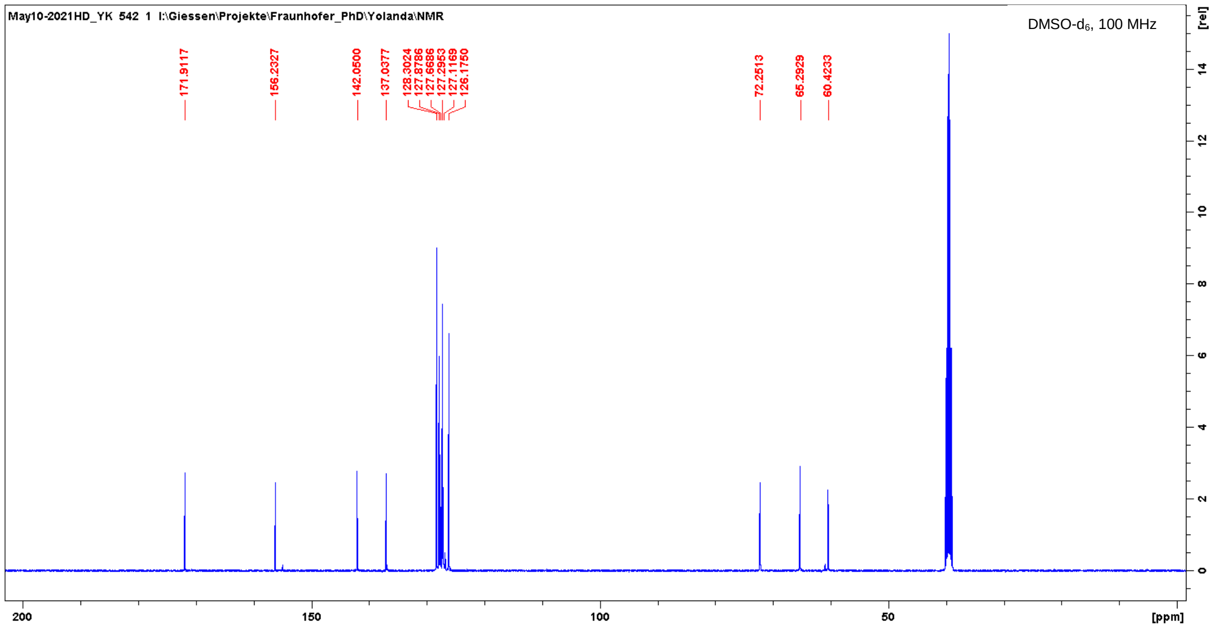
May10-2021HD_YK 542 1 I:\Giessen\Projekte\Fraunhofer_PhD\Yolanda\NMR
DMSO-d6, 100 MHz
171.9117
156.2327
142.0500
137.0377
72.2513
65.2929
60.4233
[ppm]
[rel]

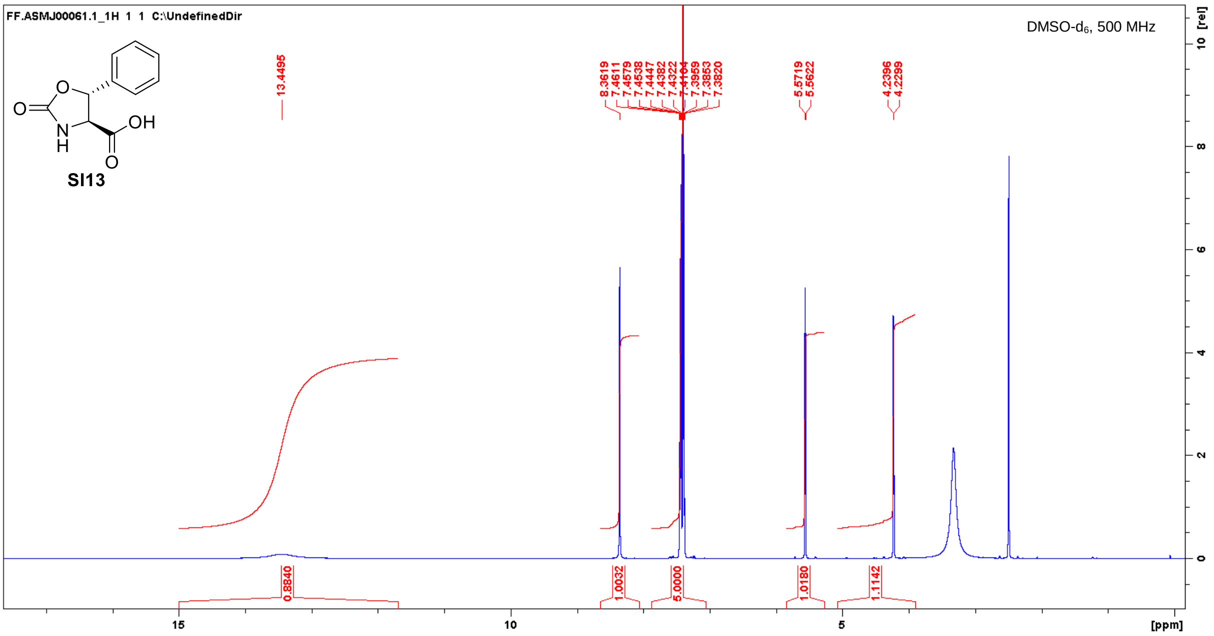
FF.ASMJ00061.1_1H 1 1 C:\UndefinedDir
DMSO-d6, 500 MHz
SI13
13.4495
8.3619
7.4611
7.4579
7.4538
7.4447
7.4382
7.4322
7.4104
7.3959
7.3853
7.3820
5.5719
5.5622
4.2396
4.2299
0.8840
1.0032
5.0000
1.0180
1.1142
[ppm]
[rel]

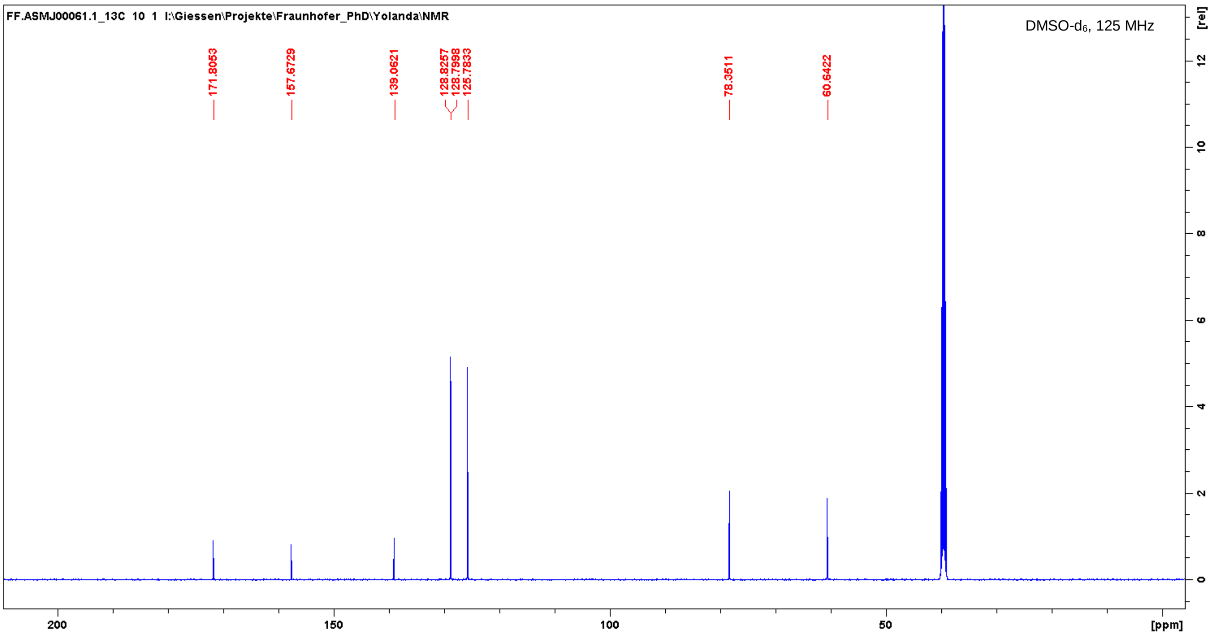
FF.ASMJ00061.1_13C 10 1 I:\Giessen\Projekte\Fraunhofer_PhD\Yolanda\NMR
DMSO-d6, 125 MHz
171.8053
157.6729
139.0621
128.8257
128.7998
125.7833
78.3511
60.6422
[ppm]
[rel]

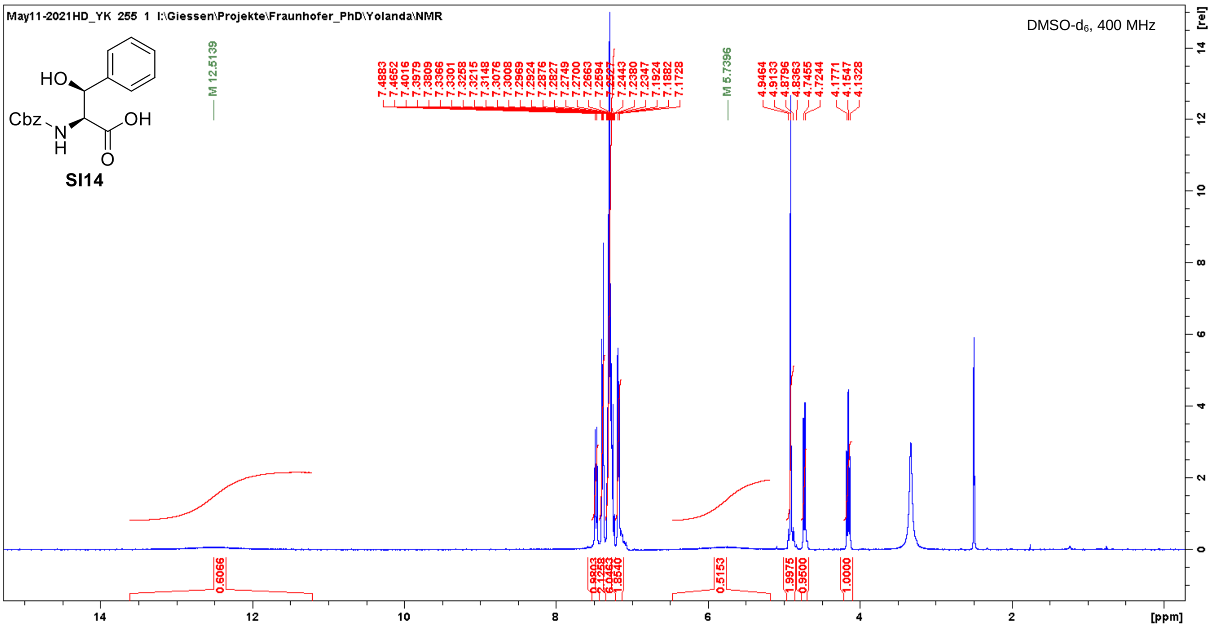
May11-2021HD_YK 255 1 I:\Giessen\Projekte\Fraunhofer_PhD\Yolanda\NMR
DMSO-d6, 400 MHz
SI14
HO
Cbz
N
H
OH
O
M 12.5139
M 5.7396
[ppm]
[rel]

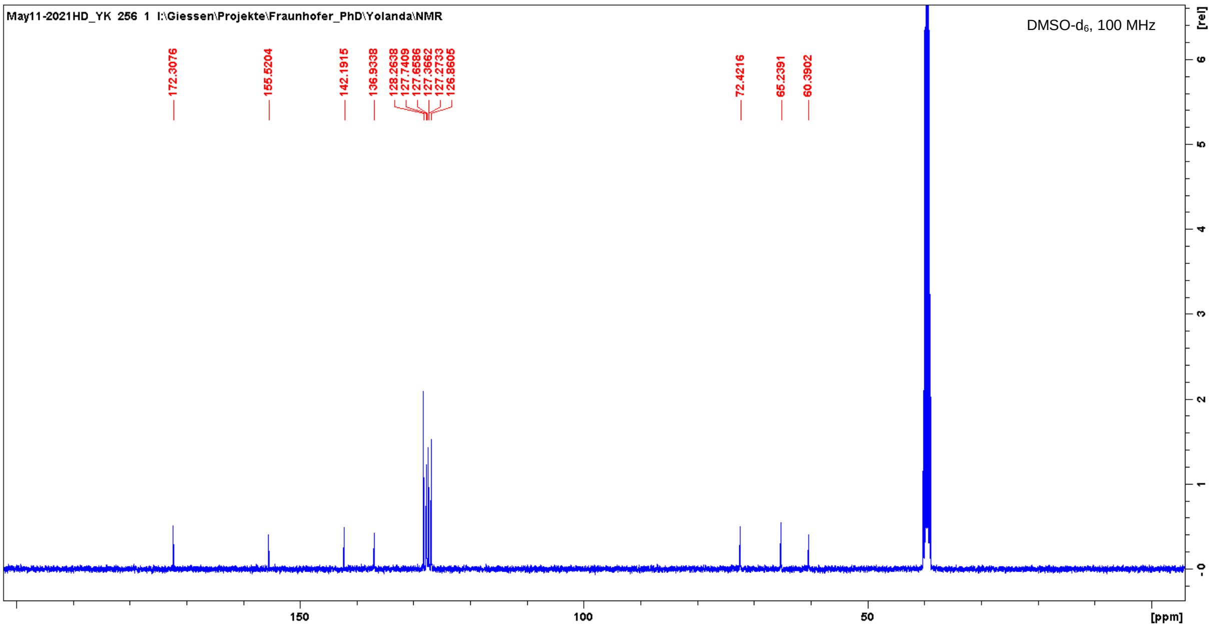
May11-2021HD_YK 256 1 I:\Giessen\Projekte\Fraunhofer_PhD\Yolanda\NMR
DMSO-d6, 100 MHz
172.3076
155.5204
142.1915
136.9338
128.2638
127.7409
127.5686
127.3662
127.2733
126.8605
72.4216
65.2391
60.3902
[ppm]
[rel]

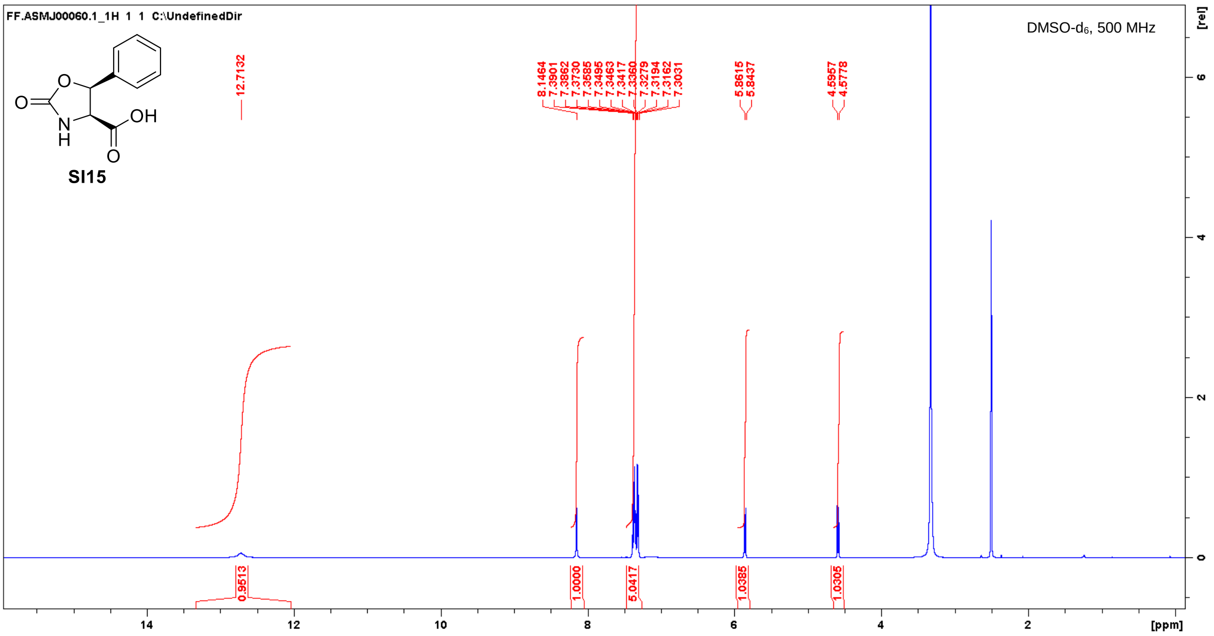
FF.ASMJ00060.1_1H 1 1 C:\UndefinedDir
DMSO-d6, 500 MHz
SI15
12.7132
8.1464
7.3901
7.3862
7.3730
7.3585
7.3495
7.3463
7.3417
7.3360
7.3279
7.3194
7.3162
7.3031
5.8615
5.8437
4.5957
4.5778
0.9513
1.0000
5.0417
1.0385
1.0305
[ppm]
[rel]

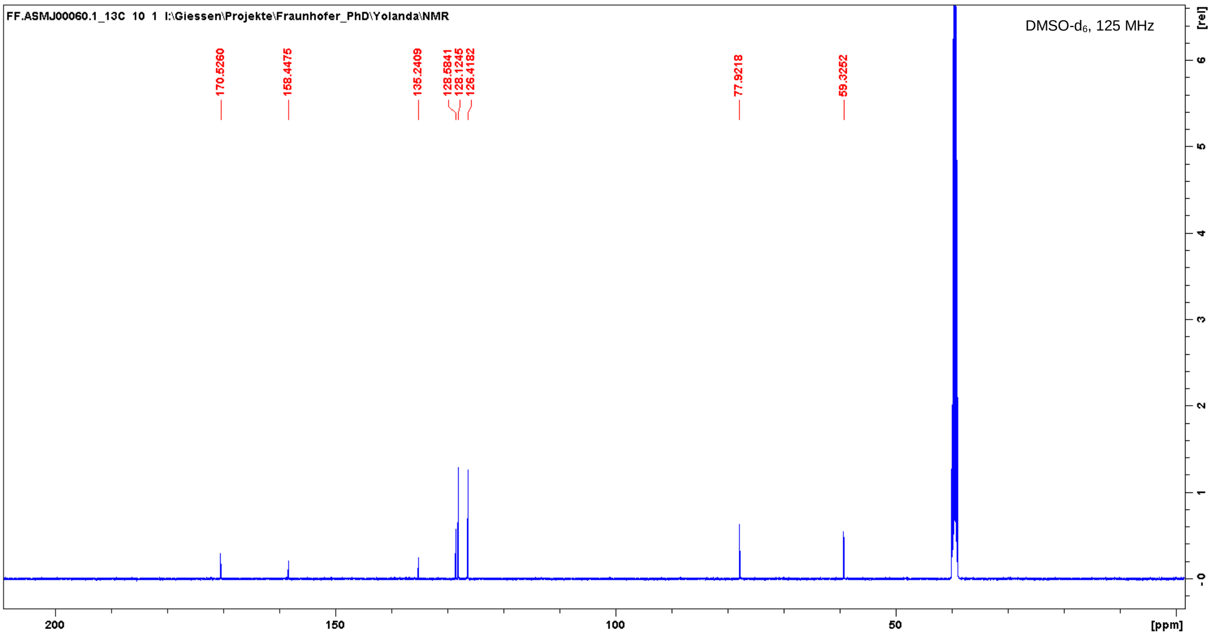
FF.ASMJ00060.1_13C 10 1 I:\Giessen\Projekte\Fraunhofer_PhD\Yolanda\NMR
DMSO-d6, 125 MHz
170.5260
158.4475
135.2409
128.5841
128.1245
126.4182
77.9218
59.3252
[ppm]
[rel]

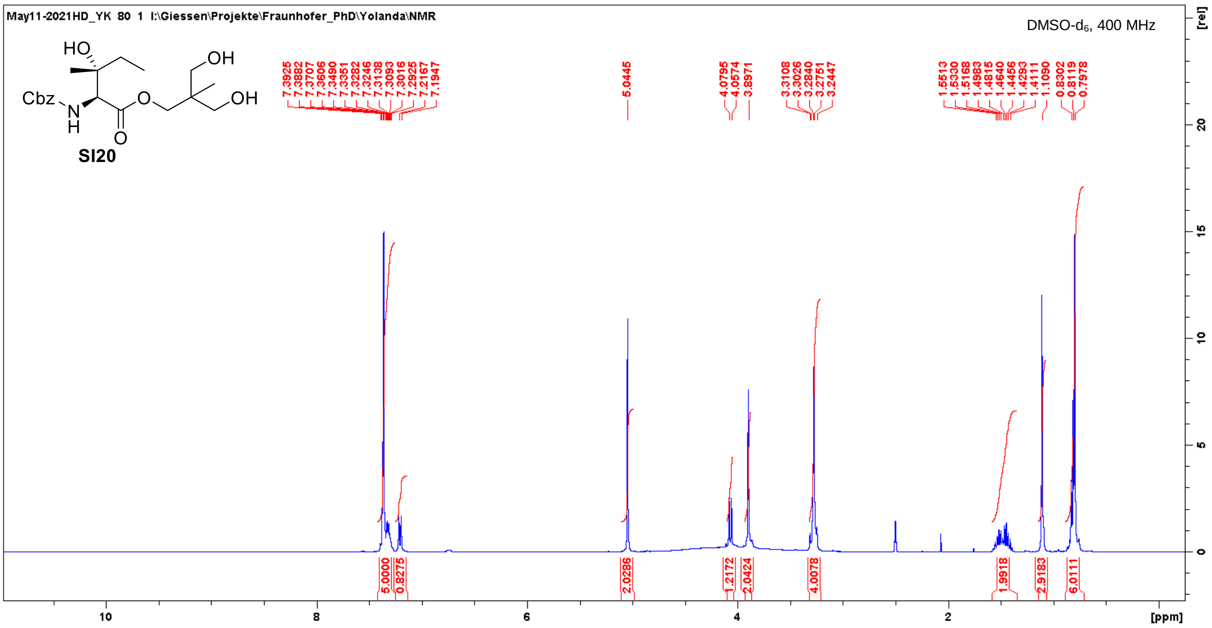

May11-2021HD_YK 80 1 I:\Giessen\Projekte\Fraunhofer_PhD\Yolanda\NMR
DMSO-d6, 400 MHz
HO
OH
Cbz
N
H
O
O
OH
SI20
7.3925
7.3882
7.3707
7.3606
7.3490
7.3351
7.3282
7.3246
7.3138
7.3093
7.3016
7.2925
7.2167
7.1947
5.0445
4.0795
4.0574
3.8971
3.3108
3.3026
3.2840
3.2751
3.2447
1.5513
1.5330
1.5168
1.4983
1.4815
1.4640
1.4456
1.4293
1.4111
1.1090
0.8302
0.8119
0.7978
5.0000
0.8275
2.0286
1.2172
2.0424
4.0078
1.9918
2.9183
6.0111
[ppm]
[rel]

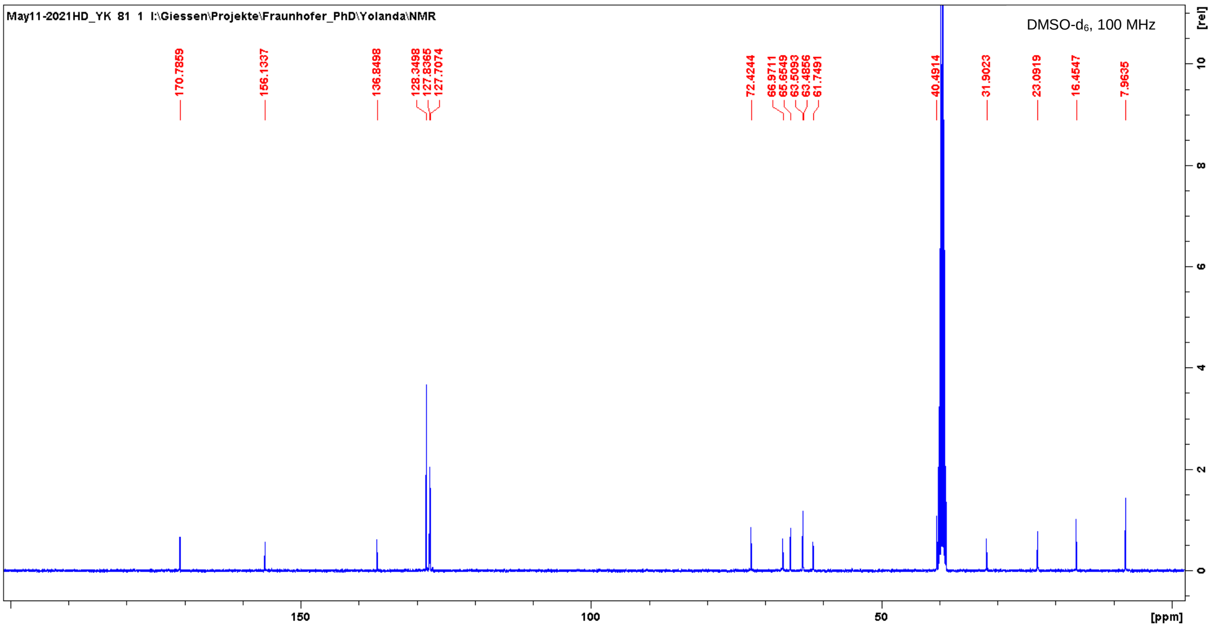

May11-2021HD_YK 81 1 I:\Giessen\Projekte\Fraunhofer_PhD\Yolanda\NMR
DMSO-d6, 100 MHz
170.7859
156.1337
136.8498
128.3498
127.8365
127.7074
72.4244
66.9711
65.6549
63.5093
63.4856
61.7491
40.4914
31.9023
23.0919
16.4547
7.9635
[ppm]
[rel]

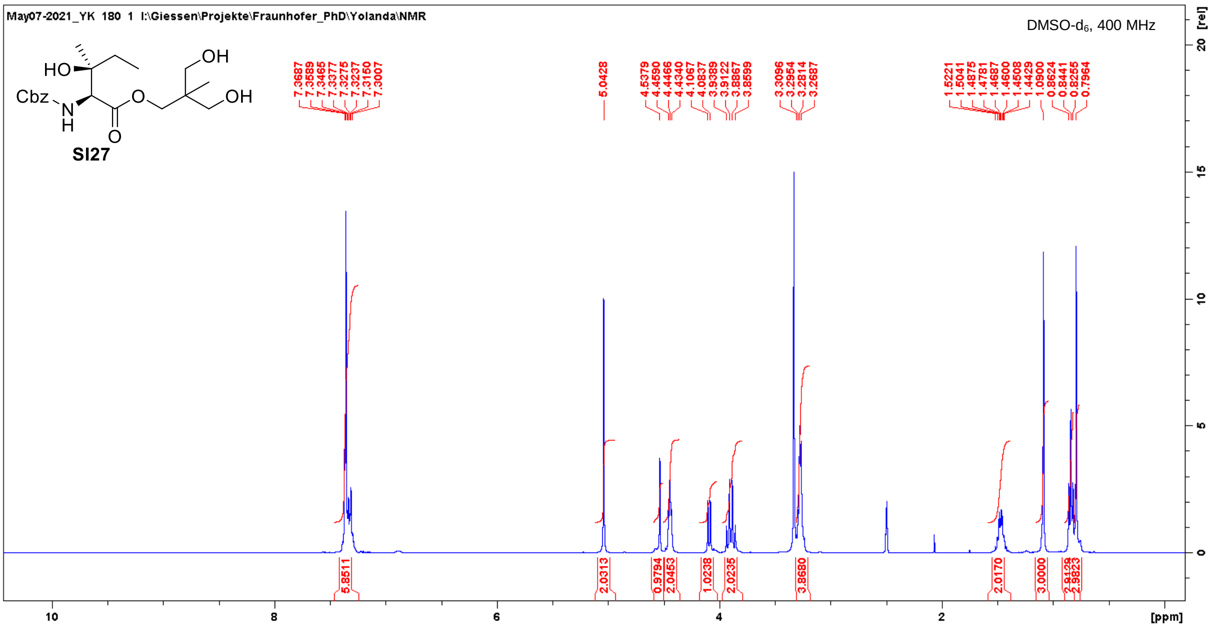
May07-2021_YK 180 1 I:\Giessen\Projekte\Fraunhofer_PhD\Yolanda\NMR
DMSO-d6, 400 MHz
SI27
[ppm]
[rel]

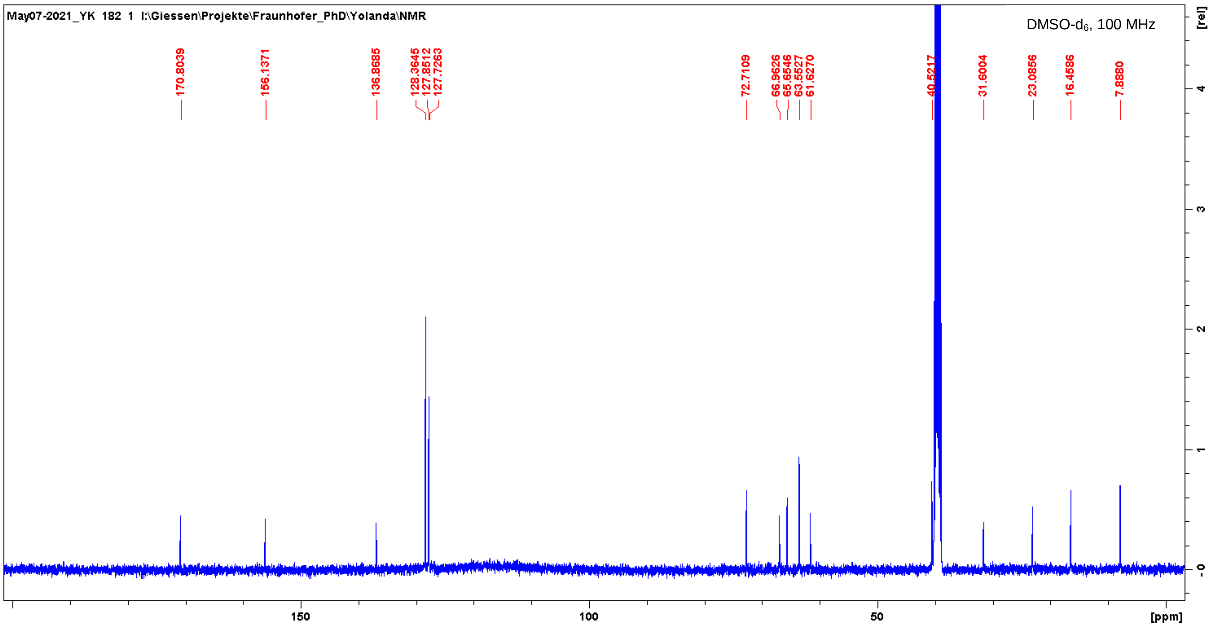
May07-2021_YK 182 1 I:\Giessen\Projekte\Fraunhofer_PhD\Yolanda\NMR
DMSO-d6, 100 MHz
170.8039
156.1371
136.8685
128.3645
127.8512
127.7263
72.7109
40.5217
31.6004
23.0856
16.4586
7.8880
[ppm]
[rel]

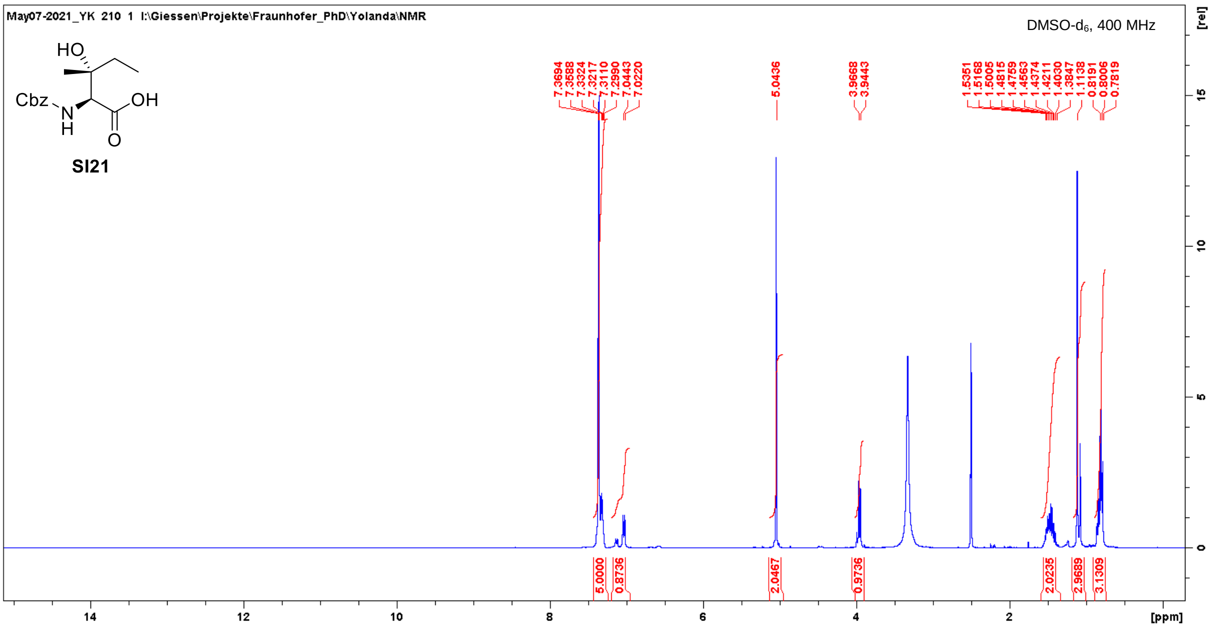
May07-2021_YK 210 1 I:\Giessen\Projekte\Fraunhofer_PhD\Yolanda\NMR
DMSO-d6, 400 MHz
HO
Cbz
N
H
OH
O
SI21
7.3694
7.3588
7.3324
7.3217
7.3110
7.2990
7.0443
7.0220
5.0436
3.9668
3.9443
1.5351
1.5168
1.5005
1.4815
1.4759
1.4563
1.4374
1.4211
1.4030
1.3847
1.1138
0.8191
0.8006
0.7819
5.0000
0.8736
2.0467
0.9736
2.0235
2.9689
3.1309
[ppm]
[rel]

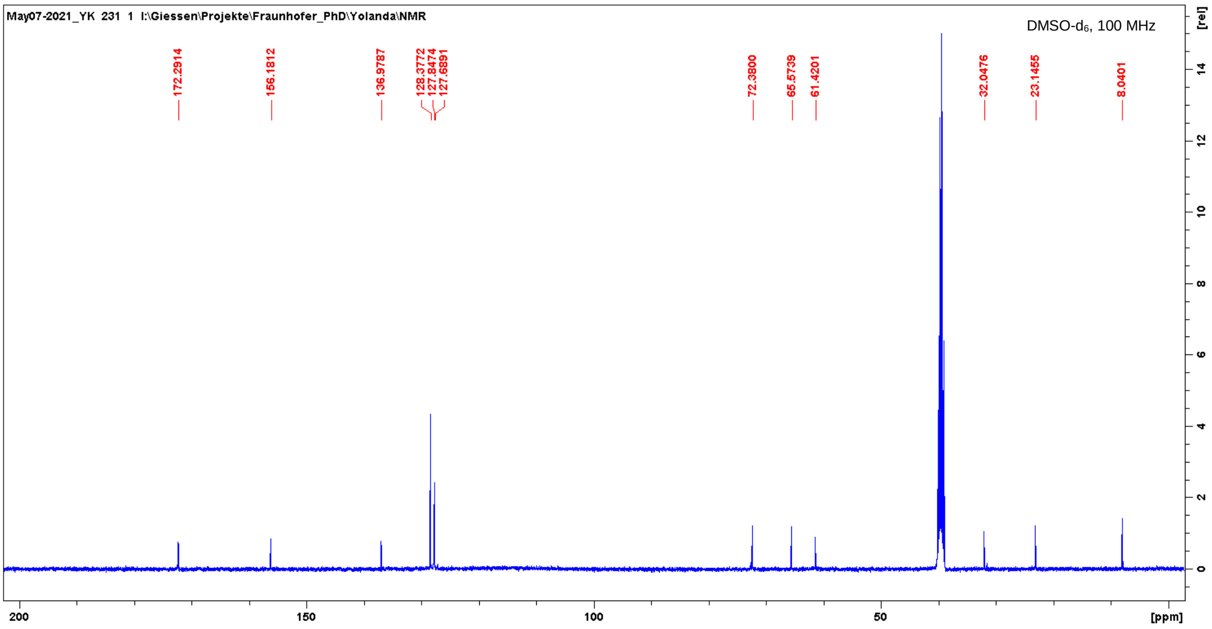
May07-2021_YK 231 1 I:\Giessen\Projekte\Fraunhofer_PhD\Yolanda\NMR
DMSO-d6, 100 MHz
172.2914
156.1812
136.9787
128.3772
127.8474
127.6891
72.3800
65.5739
61.4201
32.0476
23.1455
8.0401
[ppm]
[rel]

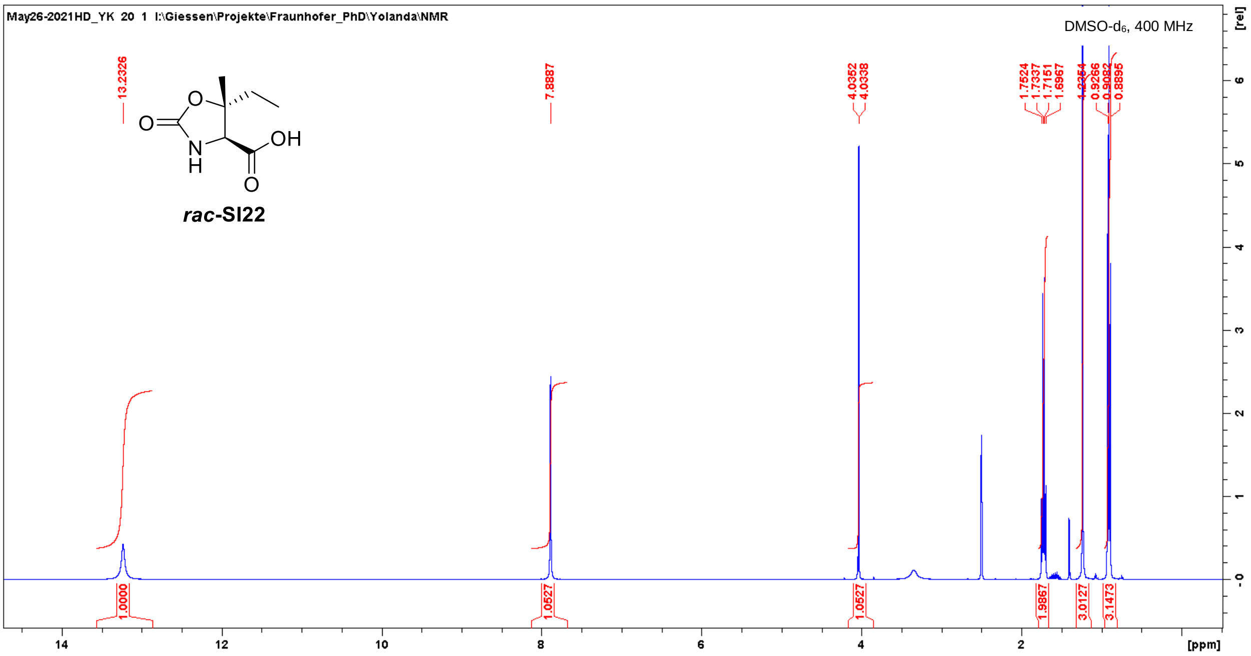
May26-2021HD_YK 20 1 I:\Giessen\Projekte\Fraunhofer_PhD\Yolanda\NMR
DMSO-d6, 400 MHz
rac-SI22
13.2326
7.8887
4.0352
4.0338
1.7524
1.7337
1.7151
1.6967
1.2254
0.9266
0.9082
0.8895
1.0000
1.0527
1.0527
1.9867
3.0127
3.1473
[ppm]
[rel]

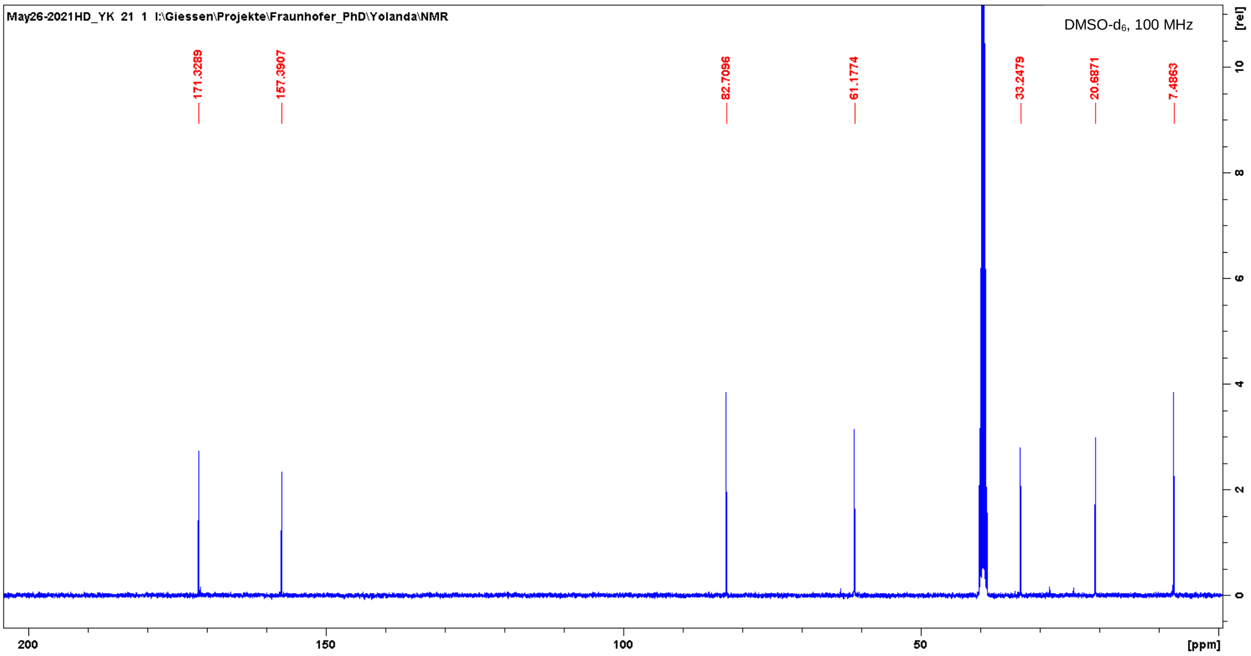
May26-2021HD_YK 21 1 I:\Giessen\Projekte\Fraunhofer_PhD\Yolanda\NMR
DMSO-d6, 100 MHz
171.3289
157.3907
82.7096
61.1774
33.2479
20.6871
7.4863
[ppm]
[rel]

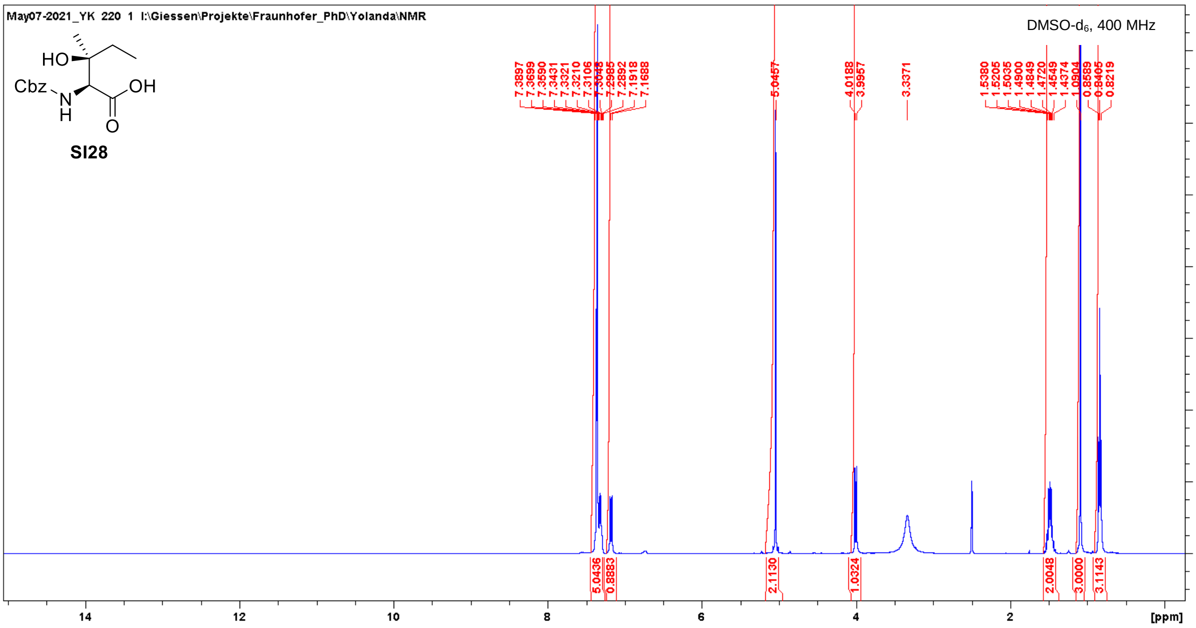
May07-2021_YK 220 1 I:\Giessen\Projekte\Fraunhofer_PhD\Yolanda\NMR
DMSO-d6, 400 MHz
SI28
7.3897 7.3699 7.3590 7.3431 7.3321 7.3210 7.3106 7.3048 7.2985 7.2892 7.1918 7.1688
5.0457
4.0188 3.9957
3.3371
1.5380 1.5205 1.5035 1.4900 1.4849 1.4720 1.4549 1.4374 1.0904 0.8589 0.8405 0.8219
5.0436 0.8883 2.1130 1.0324 2.0048 3.0000 3.1143
[ppm]
[rel]

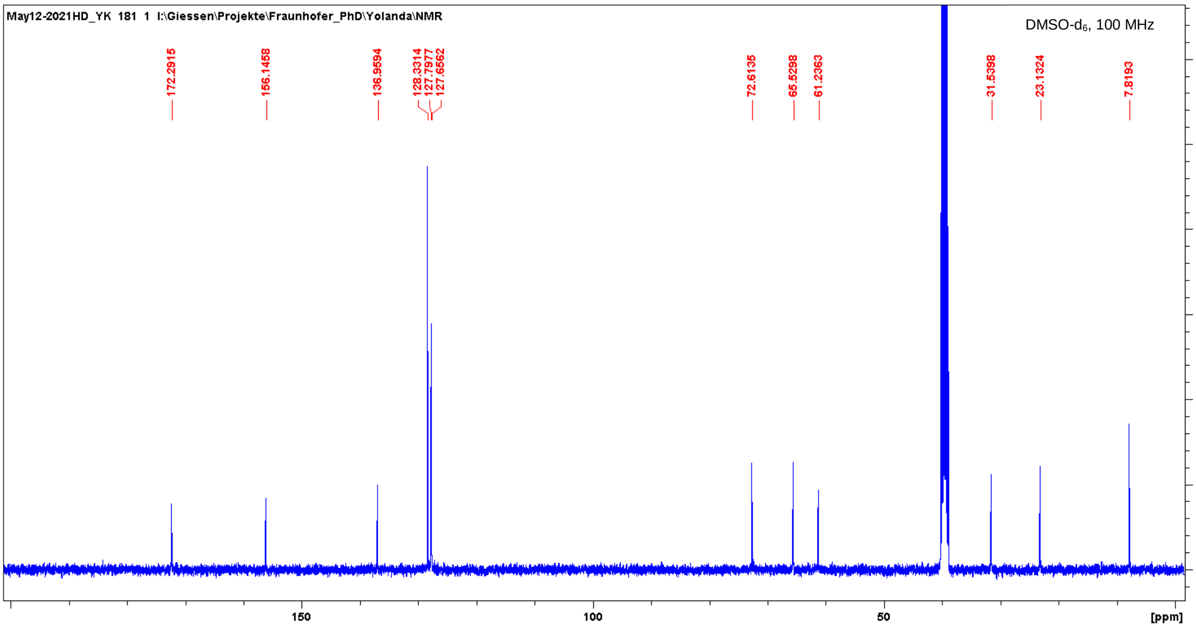
May12-2021HD_YK 181 1 I:\Giessen\Projekte\Fraunhofer_PhD\Yolanda\NMR
DMSO-d6, 100 MHz
172.2915
156.1458
136.9594
128.3314 127.7977 127.6562
72.6135
65.5298
61.2363
31.5398
23.1324
7.8193
[ppm]
[rel]

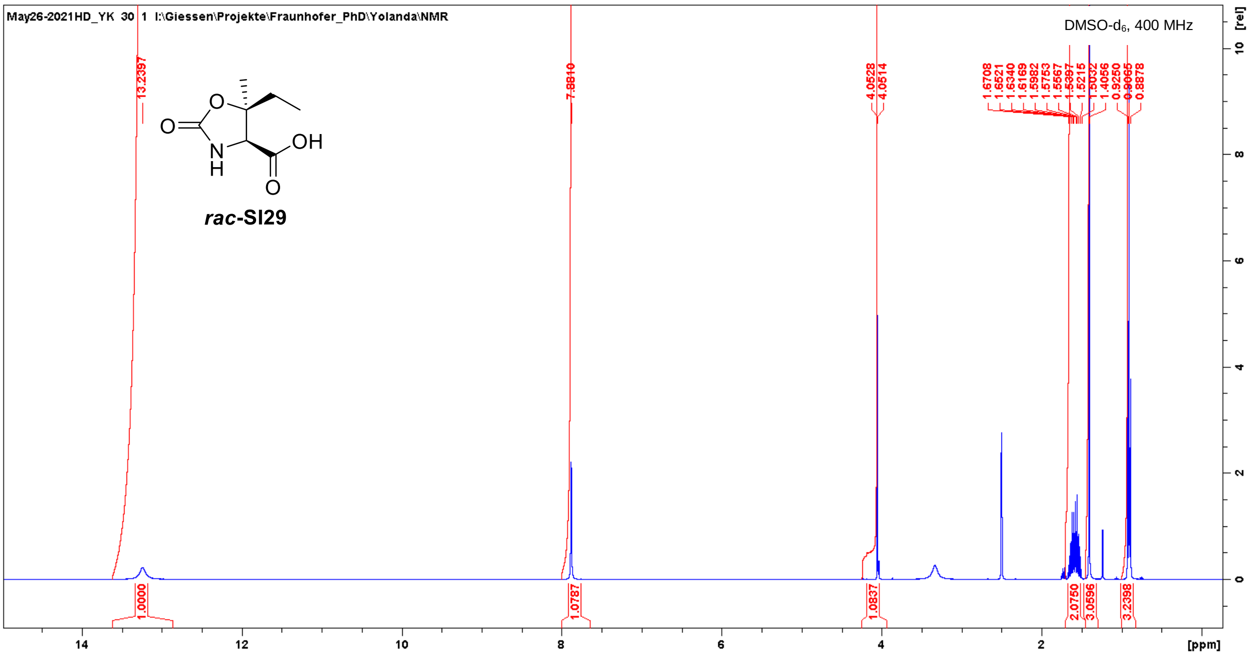
May26-2021HD_YK 30 1 I:\Giessen\Projekte\Fraunhofer_PhD\Yolanda\NMR
DMSO-d$_6$, 400 MHz
rac-SI29
13.2397
7.8810
4.0528
4.0514
1.6708
1.6521
1.6340
1.6169
1.5982
1.5763
1.5567
1.5397
1.5215
1.5032
1.4056
0.9250
0.9065
0.8878
1.0000
1.0787
1.0837
2.0750
3.0596
3.2398
[ppm]
[rel]

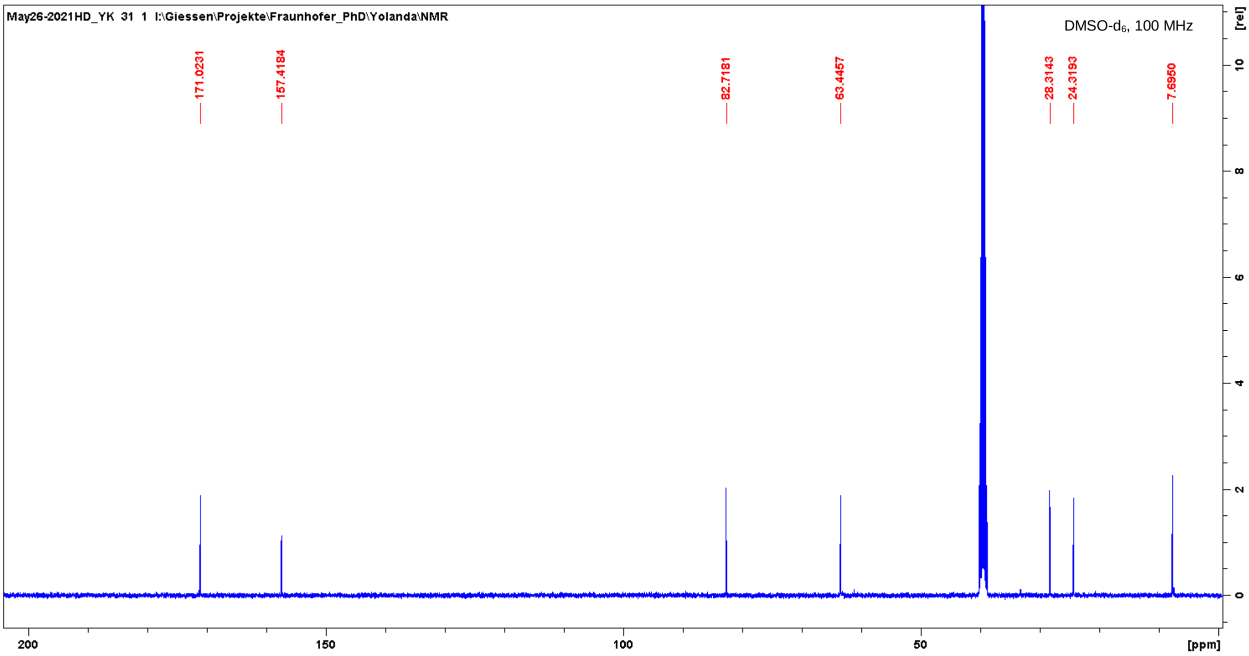
May26-2021HD_YK 31 1 I:\Giessen\Projekte\Fraunhofer_PhD\Yolanda\NMR
DMSO-d$_6$, 100 MHz
171.0231
157.4184
82.7181
63.4457
28.3143
24.3193
7.6950
[ppm]
[rel]

4.3. Determination of the enantiomeric excesses (*ee*) of synthetic β-hydroxyamino acid intermediates

Chiral Separation

Library, SM, Chiral & Peptide Purification
R&D / IDD In vitro Biology & HT Chemistry
Industriepark Höchst, G 838, Room 007
D-65926 Frankfurt
Dr. Schaffrath Tel.: +49-(0)69-305-30782

SANOFI

Sample Information:

Customer	Dr. Pöverlein
Analyst	K. Rahn-Hotze
Batch Ref No	FF.ASMJ00012.1
Sample Name	FF.ASMJ00012.1
Racemat	
Lab Journal	
Comment	

Separation Information:

APC\Poeverlein\Poeverlein_2021

HPLC-System	LC_30
Flow rate	1,0 ml/min
Temperature	30° C
HPLC Column	Chiralpak AS-H/122, 250x4,6 mm
Eluent	EtOH:MeOH 1:1

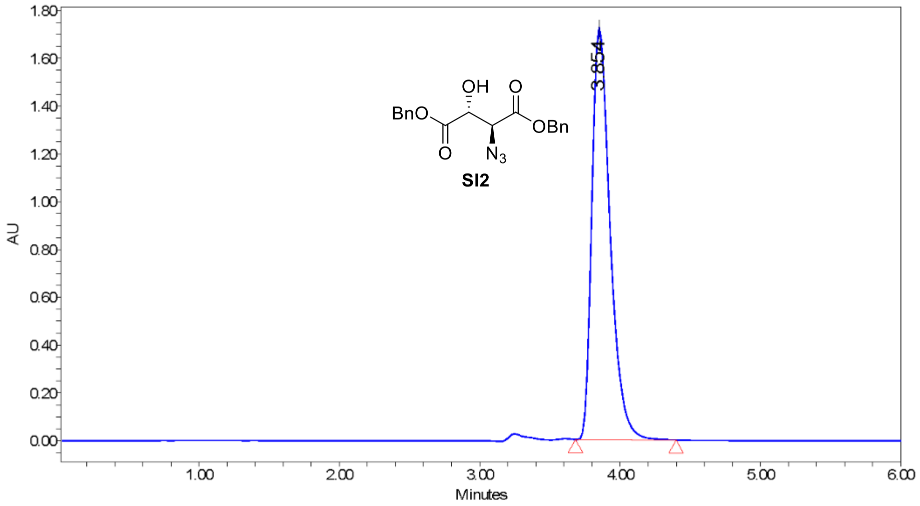

207.0nm; Inj ID 1311; Res ID 1326

Batch_Ref_No FF.ASMJ00012.1 Empower Project: APC\Poeverlein\Poeverlein_2021

Results

	RT	Area	% Area	Height
1	3.85	15542361	100.00	1726881

Current Date 4/13/2021 Vial 47 1 of 2
Date Acquired 4/13/2021 3:29:30 PM CEST Inj Vol 5.00 uL

Sample Information:

Customer Dr. Pöverlein

Batch Ref No FF.ASMJ00012.1

SampleName FF.ASMJ00012.1

Comment

Spectrum Index Plot

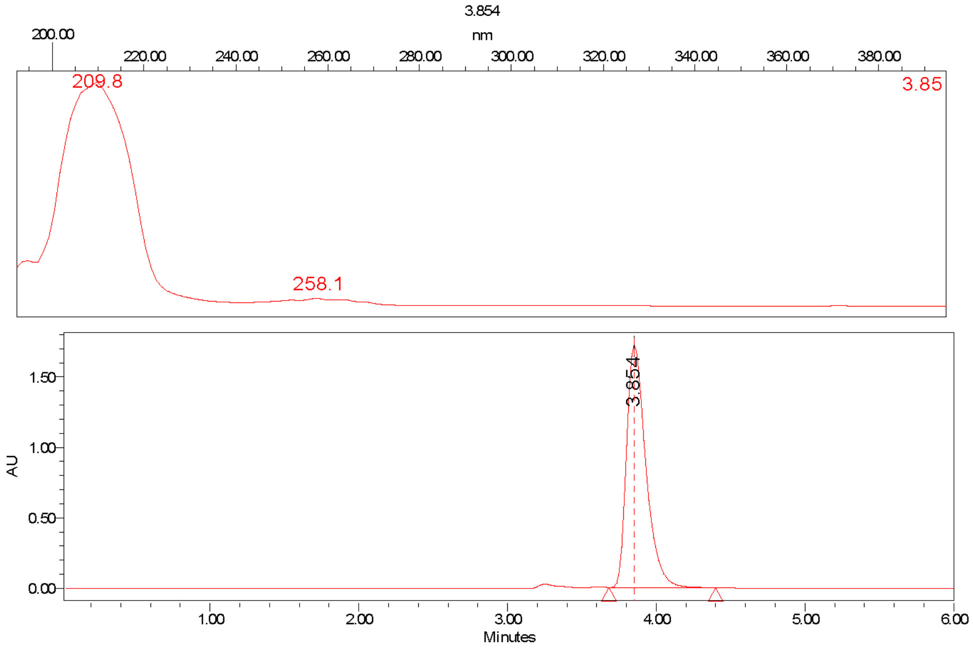

Batch_Ref_No FF.ASMJ00012.1 Project Name: APC\Poeverlein\Poeverlein_2021

Chiral Separation

Library, SM, Chiral & Peptide Purification
R&D / IDD In vitro Biology & HT Chemistry
Industriepark Höchst, G 838, Room 007
D-65926 Frankfurt
Dr. Schaffrath Tel.: +49-(0)69-305-30782

SANOFI

Sample Information:

Customer	Dr. Pöverlein
Analyst	K. Rahn-Hotze
Batch Ref No	FF.ASMJ00019.1
Sample Name	FF.ASMJ00019.1
Racemat	
Lab Journal	
Comment	

Separation Information:

APC\Poeverlein\Poeverlein_2021

HPLC-System	LC_30
Flow rate	1,0 ml/min
Temperature	30° C
HPLC Column	Chiralpak AS-H/122, 250x4,6 mm
Eluent	EtOH:MeOH 1:1

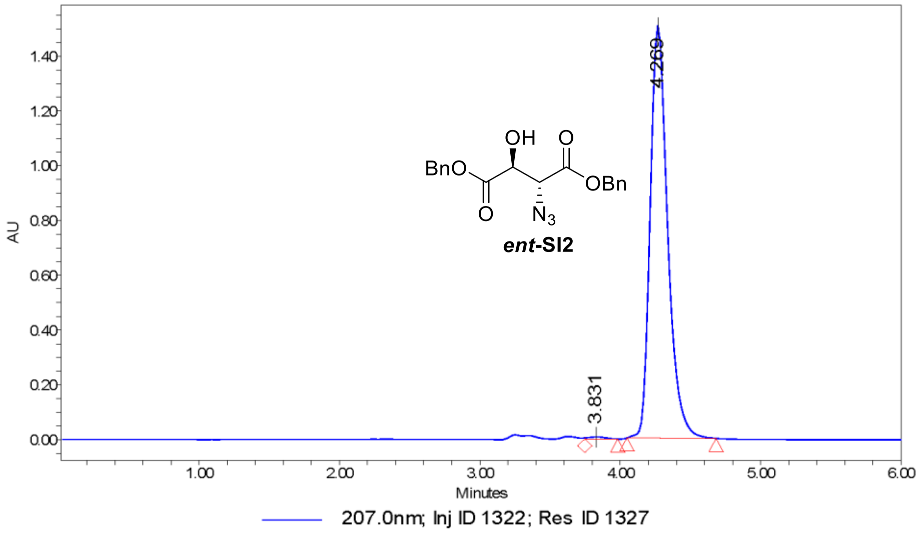

Batch_Ref_No FF.ASMJ00019.1 Empower Project: APC\Poeverlein\Poeverlein_2021

Results

	RT	Area	% Area	Height
1	3.83	65863	0.50	7974
2	4.27	12982699	99.50	1504163

Current Date 4/13/2021 Vial 48 1 of 2
Date Acquired 4/13/2021 3:42:32 PM CEST Inj Vol 2.00 uL

Sample Information:

Customer Dr. Pöverlein

Batch Ref No FF.ASMJ00019.1

SampleName FF.ASMJ00019.1

Comment

Spectrum Index Plot

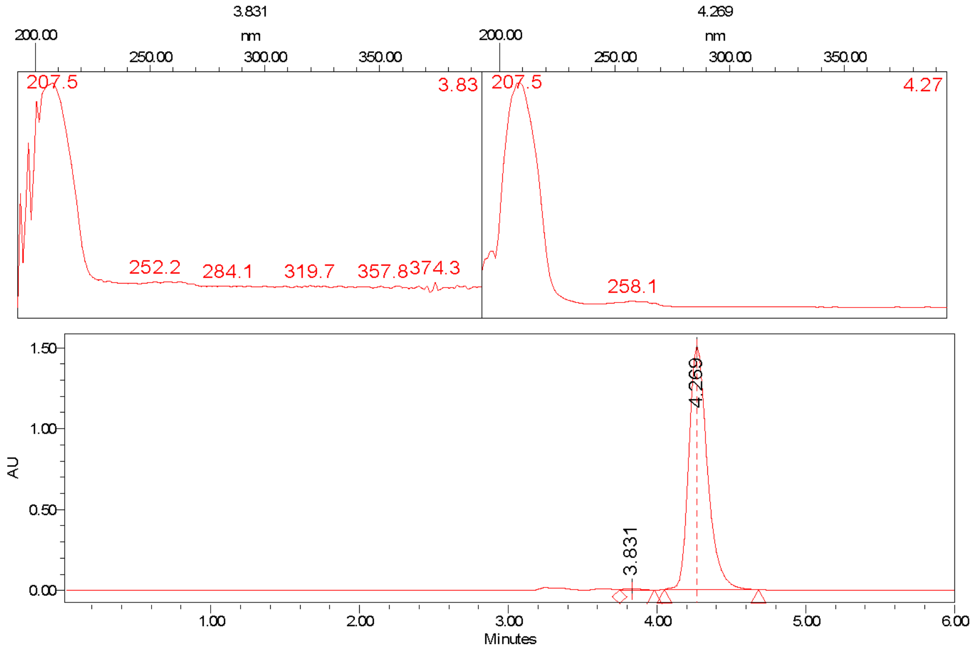

Batch_Ref_No FF.ASMJ00019.1 Project Name: APC\Poeverlein\Poeverlein_2021

Chiral Separation

Library, SM, Chiral & Peptide Purification
R&D / IDD In vitro Biology & HT Chemistry
Industriepark Höchst, G 838, Room 007
D-65926 Frankfurt
Dr. Schaffrath Tel.: +49-(0)69-305-30782

SANOFI

Sample Information:

Customer	Dr. Pöverlein
Analyst	K. Rahn-Hotze
Batch Ref No	FF.ASMJ00014.2
Sample Name	FF.ASMJ00014.2
Racemat	
Lab Journal	
Comment	

Separation Information:

APC\Poeverlein\Poeverlein_2021

HPLC-System	LC_30
Flow rate	1,0 ml/min
Temperature	30° C
HPLC Column	Chiralpak AD-H/148, 250x4,6 mm
Eluent	EtOH:MeOH 1:1

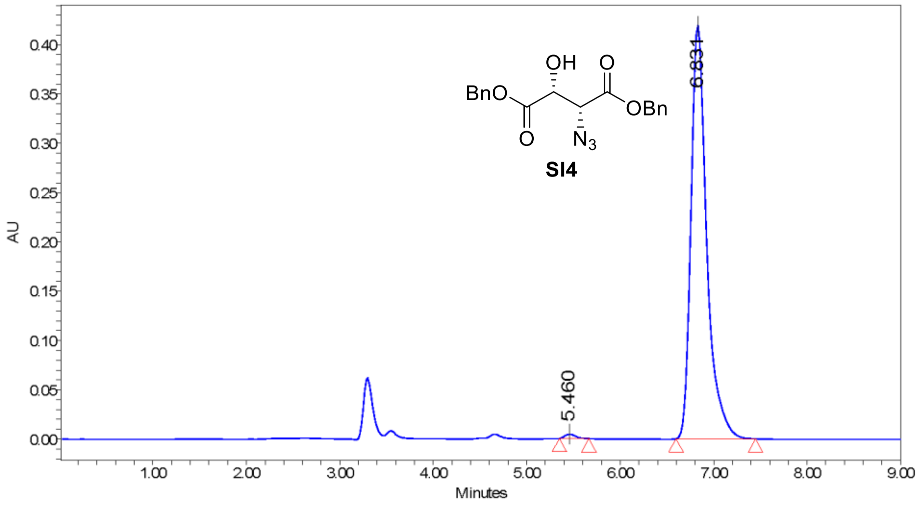

Batch_Ref_No FF.ASMJ00014.2 Empower Project: APC\Poeverlein\Poeverlein_2021

Results

	RT	Area	% Area	Height
1	5.46	34946	0.71	4388
2	6.83	4899448	99.29	419033

Current Date 4/13/2021
Date Acquired 4/13/2021 2:28:50 PM CEST

Vial 43
Inj Vol 5.00 uL

1 of 2

Sample Information:

Customer Dr. Pöverlein
Batch Ref No FF.ASMJ00014.2
SampleName FF.ASMJ00014.2
Comment

Spectrum Index Plot

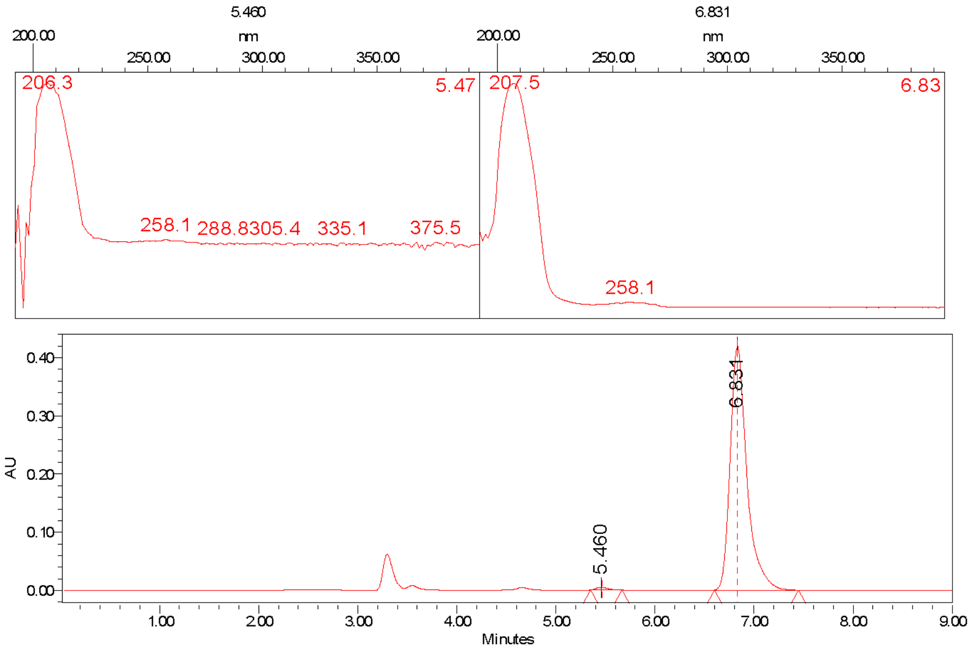

Batch_Ref_No FF.ASMJ00014.2 Project Name: APC\Poeverlein\Poeverlein_2021

Chiral Separation

Library, SM, Chiral & Peptide Purification
R&D / IDD In vitro Biology & HT Chemistry
Industriepark Höchst, G 838, Room 007
D-65926 Frankfurt
Dr. Schaffrath Tel.: +49-(0)69-305-30782

SANOFI

Sample Information:

Customer	Dr. Pöverlein
Analyst	K. Rahn-Hotze
Batch Ref No	FF.ASMJ00021.2
Sample Name	FF.ASMJ00021.2
Racemat	
Lab Journal	
Comment	

Separation Information:

APC\Poeverlein\Poeverlein_2021

HPLC-System	LC_30
Flow rate	1,0 ml/min
Temperature	30° C
HPLC Column	Chiralpak AD-H/148, 250x4,6 mm
Eluent	EtOH:MeOH 1:1

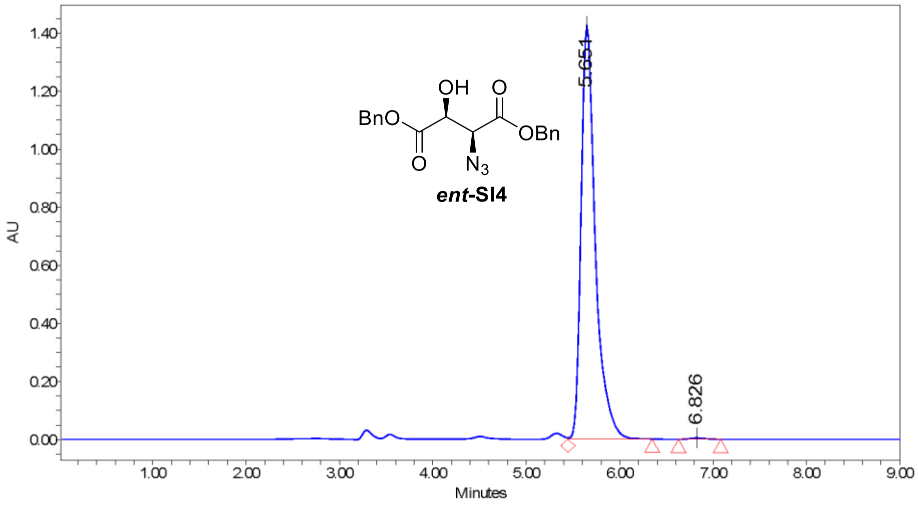

Batch_Ref_No FF.ASMJ00021.2 Empower Project: APC\Poeverlein\Poeverlein_2021

Results

	RT	Area	% Area	Height
1	5.65	15082936	99.67	1423205
2	6.83	50343	0.33	4658

Current Date 4/13/2021 Vial 44 1 of 2
Date Acquired 4/13/2021 2:38:46 PM CEST Inj Vol 5.00 uL

Sample Information:

Customer Dr. Pöverlein
Batch Ref No FF.ASMJ00021.2
SampleName FF.ASMJ00021.2
Comment

Spectrum Index Plot

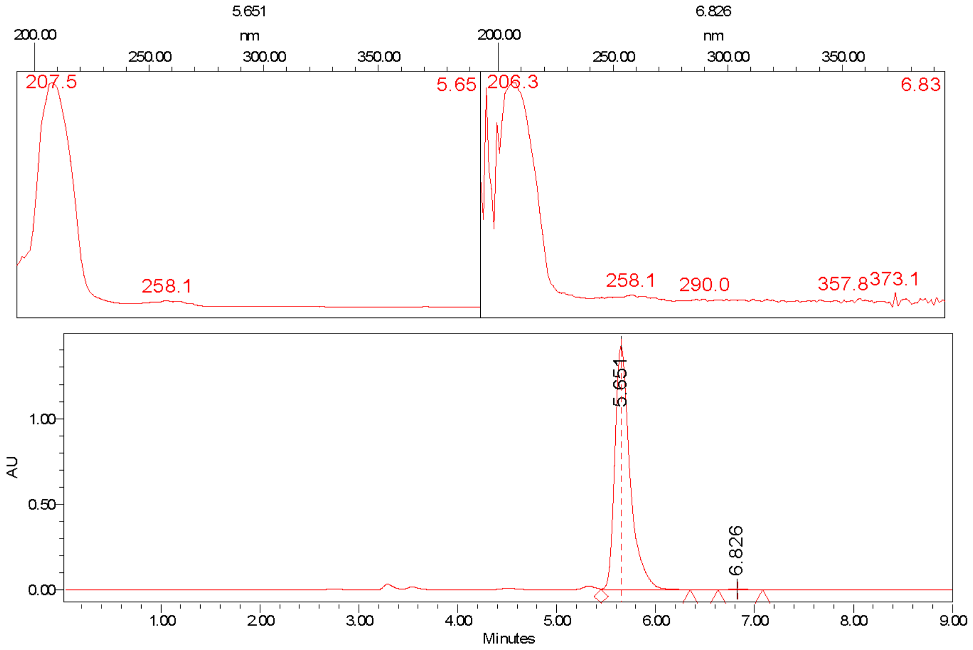

Batch_Ref_No FF.ASMJ00021.2 Project Name: APC\Poeverlein\Poeverlein_2021

Chiral Separation

Library, SM, Chiral & Peptide Purification
R&D / IDD In vitro Biology & HT Chemistry
Industriepark Höchst, G 838, Room 007
D-65926 Frankfurt
Dr. Schaffrath Tel.: +49-(0)69-305-30782

SANOFI

Sample Information:

Customer Dr. Pöverlein
Analyst K. Rahn-Hotze
Batch Ref No FF.ASMJ00075.2
Sample Name FF.ASMJ00075.2
Racemat
Lab Journal
Comment

Separation Information:

APC\Poeverlein\Poeverlein_2021
HPLC-System LC_03
Flow rate 1,0 ml/min
Temperature 30° C
HPLC Column Chiralpak ID/174, 250x4,6 mm
Eluent Hep:EtOH:MeOH 5:1:1 + 0,1% TFA

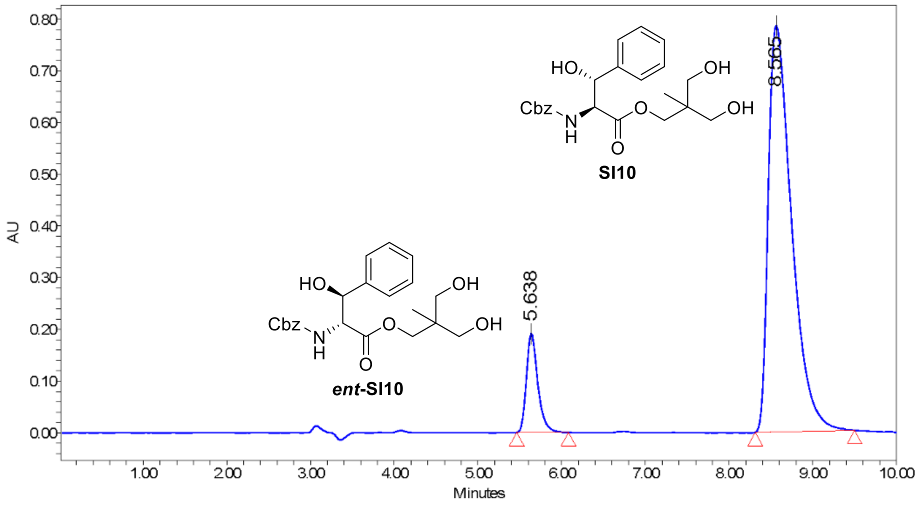

Batch_Ref_No FF.ASMJ00075.2 Empower Project: APC\Poeverlein\Poeverlein_2021

Results

	RT	Area	% Area	Height
1	5.64	1817870	10.68	191839
2	8.56	15197037	89.32	785502

Current Date 5/19/2021 Vial 32 1 of 2
Date Acquired 5/18/2021 3:34:03 PM CEST Inj Vol 2.00 uL

Sample Information:

Customer	Dr. Pöverlein
Batch Ref No	FF.ASMJ00075.2
SampleName	FF.ASMJ00075.2
Comment	

Spectrum Index Plot

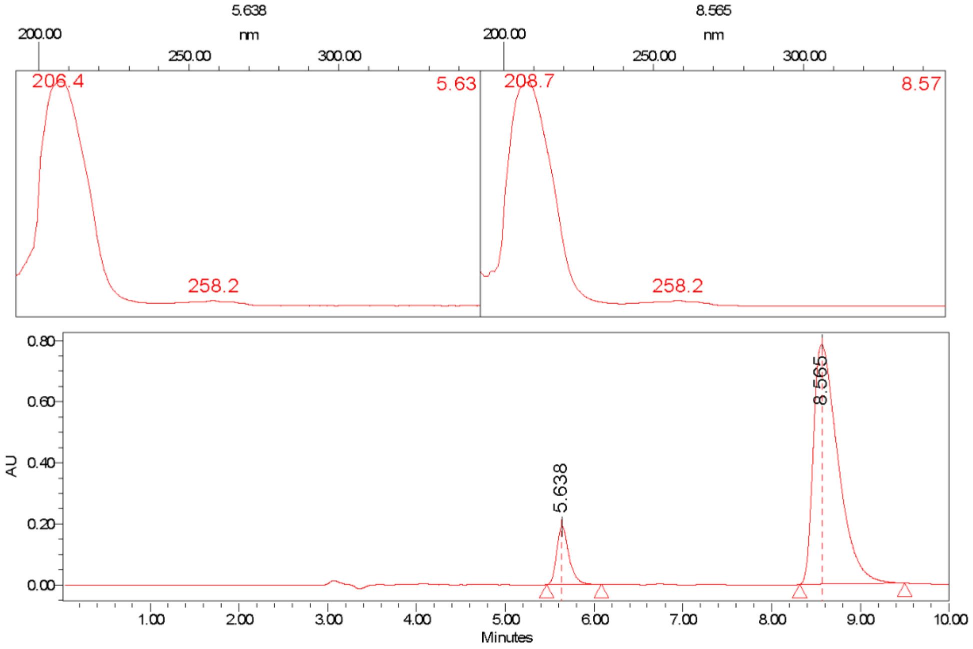

Batch_Ref_No FF.ASMJ00075.2 Project Name: APC\Poeverlein\Poeverlein_2021

Chiral Separation

Library, SM, Chiral & Peptide Purification
R&D / IDD In vitro Biology & HT Chemistry
Industriepark Höchst, G 838, Room 007
D-65926 Frankfurt
Dr. Schaffrath Tel.: +49-(0)69-305-30782

SANOFI

Sample Information:

Customer	Dr. Pöverlein
Analyst	K. Rahn-Hotze
Batch Ref No	FF.ASMJ00072.2
Sample Name	FF.ASMJ00072.2
Racemat	
Lab Journal	
Comment	

Separation Information:

APC\Poeverlein\Poeverlein_2021

HPLC-System	LC_03
Flow rate	1,0 ml/min
Temperature	30° C
HPLC Column	Chiralpak ID/174, 250x4,6 mm
Eluent	Hep:EtOH:MeOH 5:1:1 + 0,1% TFA

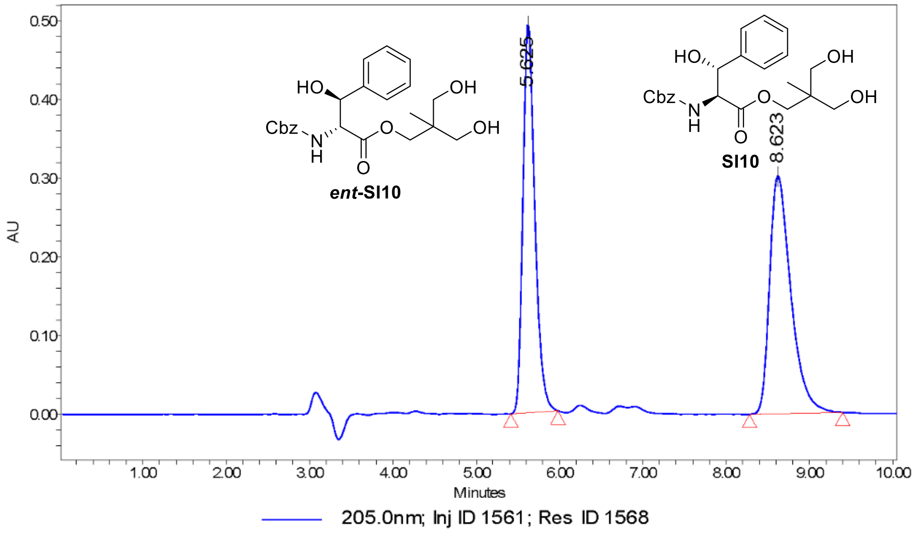

Batch_Ref_No FF.ASMJ00072.2 Empower Project: APC\Poeverlein\Poeverlein_2021

Results

	RT	Area	% Area	Height
1	5.62	4892494	47.71	496270
2	8.62	5362392	52.29	302904

Current Date 5/19/2021 Vial 26 1 of 2
Date Acquired 5/18/2021 10:18:44 AM CEST Inj Vol 5.00 uL

Sample Information:

Customer Dr. Pöverlein
Batch Ref No FF.ASMJ00072.2
SampleName FF.ASMJ00072.2
Comment

Spectrum Index Plot

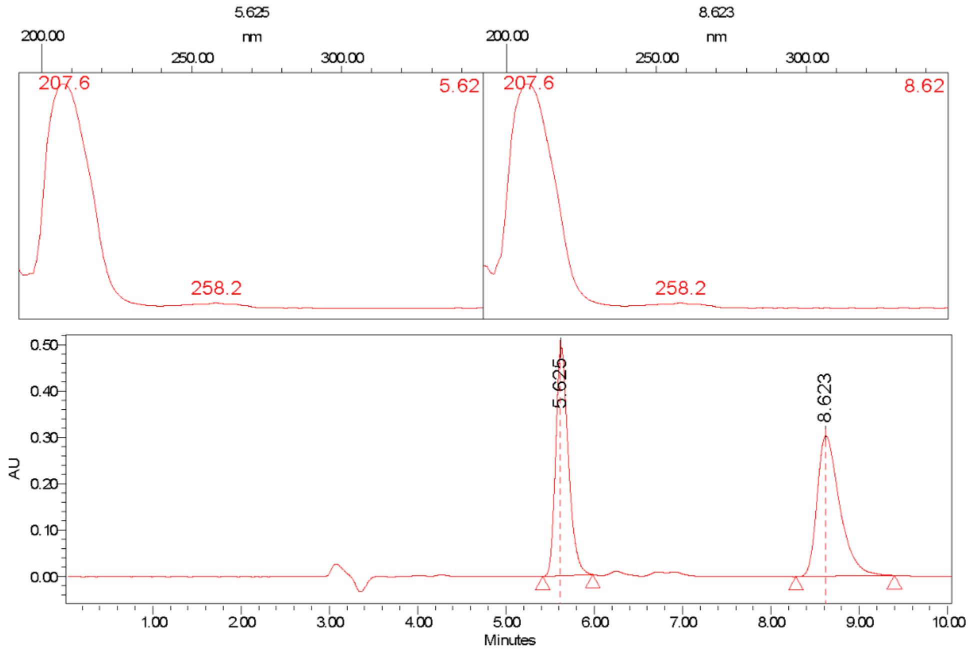

Batch_Ref_No FF.ASMJ00072.2 Project Name: APC\Poeverlein\Poeverlein_2021

Current Date 5/19/2021 Vial 26
Date Acquired 5/18/2021 10:18:44 AM CEST Inj Vol 5.00 uL

Chiral Separation

Library, SM, Chiral & Peptide Purification
R&D / IDD In vitro Biology & HT Chemistry
Industriepark Höchst, G 838, Room 007
D-65926 Frankfurt
Dr. Schaffrath Tel.: +49-(0)69-305-30782

SANOFI

Sample Information:

Customer	Dr. Pöverlein
Analyst	K. Rahn-Hotze
Batch Ref No	FF.ASMJ00075.1
Sample Name	FF.ASMJ00075.1
Racemat	
Lab Journal	
Comment	

Separation Information:

APC\Poeverlein\Poeverlein_2021

HPLC-System	LC_03
Flow rate	1,0 ml/min
Temperature	30° C
HPLC Column	Chiralpak IF/181, 250x4,6 mm
Eluent	Hep:EtOH:MeOH 5:1:1 + 0,1% TFA

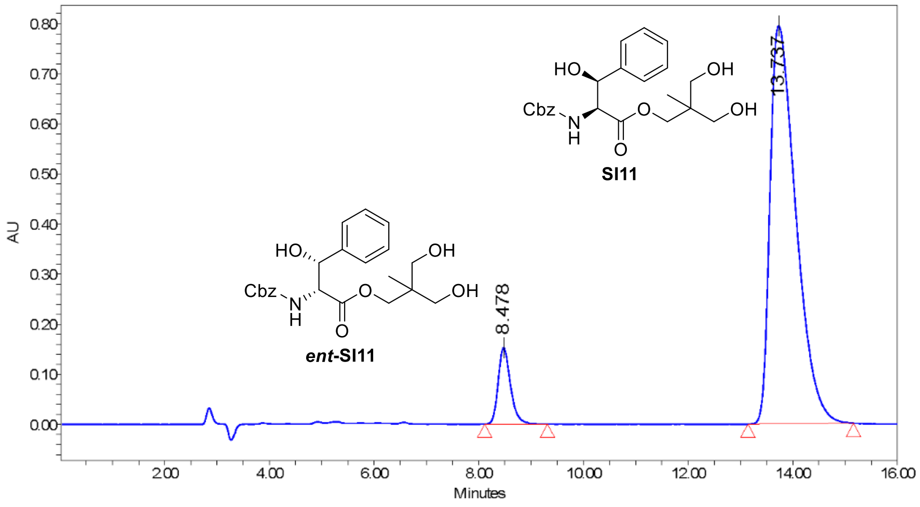

205.0nm; Inj ID 1587; Res ID 1592

Batch_Ref_No FF.ASMJ00075.1 Empower Project: APC\Poeverlein\Poeverlein_2021

Results

	RT	Area	% Area	Height
1	8.48	2488866	8.38	152577
2	13.74	27228681	91.62	794500

Current Date 5/19/2021 Vial 29 1 of 2
Date Acquired 5/18/2021 2:30:24 PM CEST Inj Vol 5.00 uL

Sample Information:

Customer Dr. Pöverlein
Batch Ref No FF.ASMJ00075.1
SampleName FF.ASMJ00075.1
Comment

Spectrum Index Plot

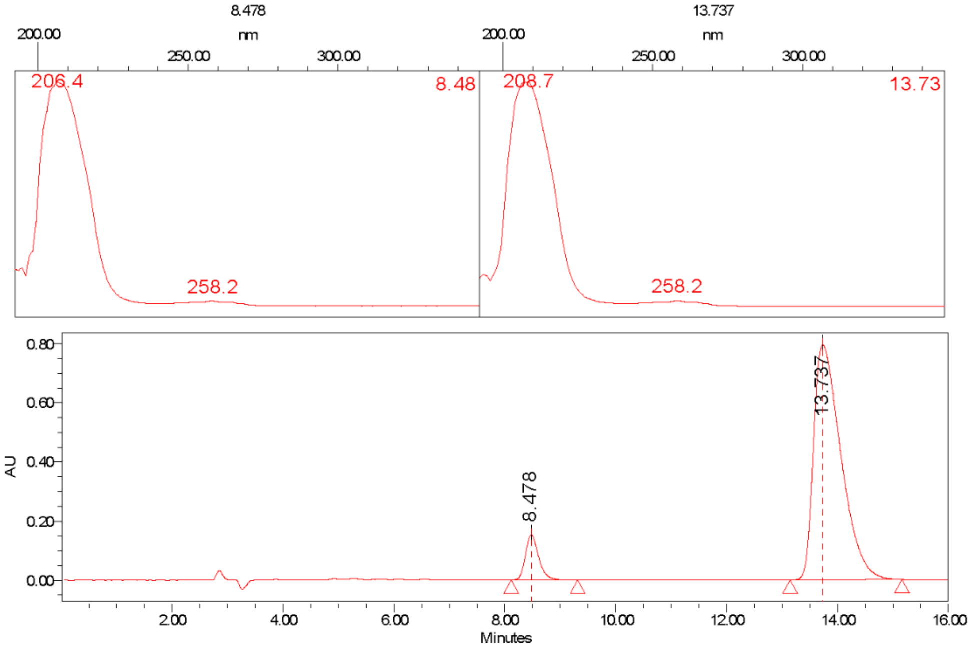

Batch_Ref_No FF.ASMJ00075.1 Project Name: APC\Poeverlein\Poeverlein_2021

Chiral Separation

Library, SM, Chiral & Peptide Purification
R&D / IDD In vitro Biology & HT Chemistry
Industriepark Höchst, G 838, Room 007
D-65926 Frankfurt
Dr. Schaffrath Tel.: +49-(0)69-305-30782

SANOFI

Sample Information:

Customer	Dr. Pöverlein
Analyst	K. Rahn-Hotze
Batch Ref No	FF.ASMJ00072.1
Sample Name	FF.ASMJ00072.1
Racemat	
Lab Journal	
Comment	

Separation Information:

APC\Poeverlein\Poeverlein_2021

HPLC-System	LC_03
Flow rate	1,0 ml/min
Temperature	30° C
HPLC Column	Chiralpak IF/181, 250x4,6 mm
Eluent	Hep:EtOH:MeOH 5:1:1 + 0,1% TFA

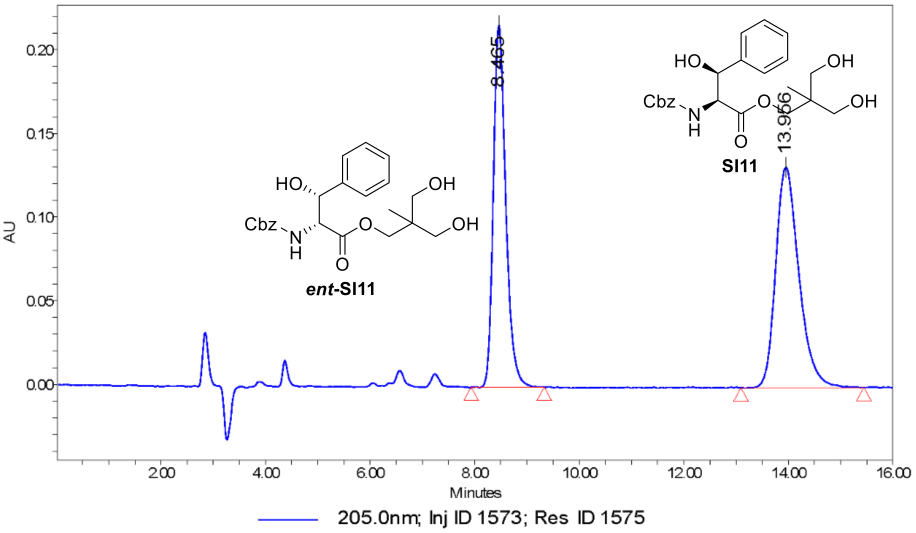

Batch_Ref_No FF.ASMJ00072.1 Empower Project: APC\Poeverlein\Poeverlein_2021

Results

	RT	Area	% Area	Height
1	8.46	3565304	46.77	216120
2	13.96	4057665	53.23	131638

Current Date 5/19/2021 Vial 25 1 of 2
Date Acquired 5/18/2021 11:04:49 AM CEST Inj Vol 5.00 uL

Sample Information:

Customer	Dr. Pöverlein
Batch Ref No	FF.ASMJ00072.1
SampleName	FF.ASMJ00072.1
Comment	

Spectrum Index Plot

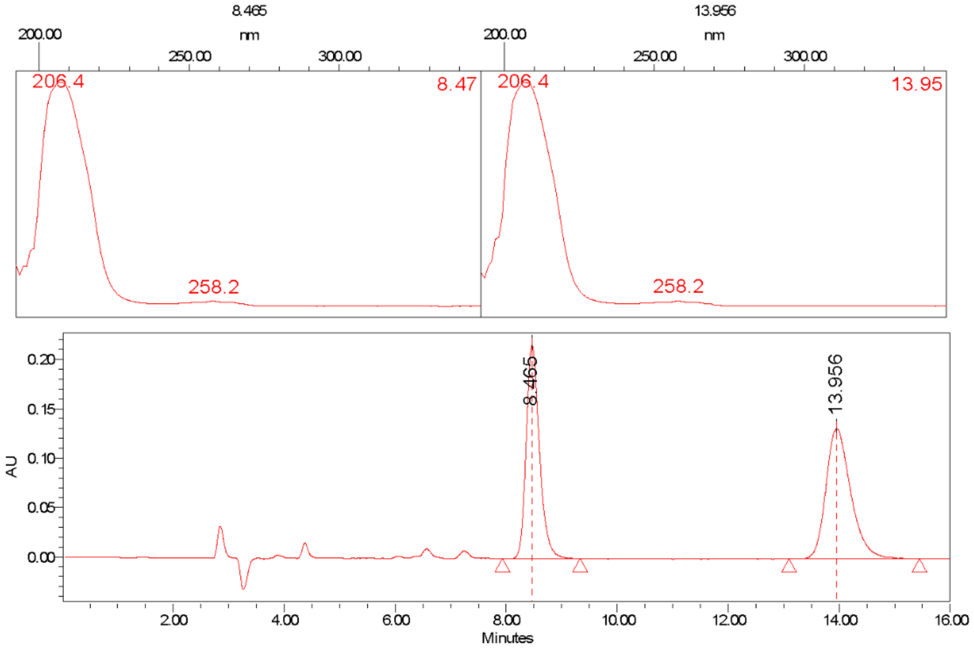

Batch_Ref_No FF.ASMJ00072.1 Project Name: APC\Poeverlein\Poeverlein_2021

Chiral Separation

Library, SM, Chiral & Peptide Purification
R&D / IDD In vitro Biology & HT Chemistry
Industriepark Höchst, G 838, Room 007
D-65926 Frankfurt
Dr. Schaffrath Tel.: +49-(0)69-305-30782

SANOFI

Sample Information:

Customer	Dr. Pöverlein
Analyst	K. Rahn-Hotze
Batch Ref No	FF.ASMJ00095.1
Sample Name	FF.ASMJ00095.1
Racemat	
Lab Journal	
Comment	

Separation Information:

APC\Poeverlein\Poeverlein_2021

HPLC-System	LC_03
Flow rate	1,0 ml/min
Temperature	30° C
HPLC Column	Chiralpak IF/181, 250x4,6 mm
Eluent	Hep:EtOH:MeOH 5:1:1 + 0,1% TFA

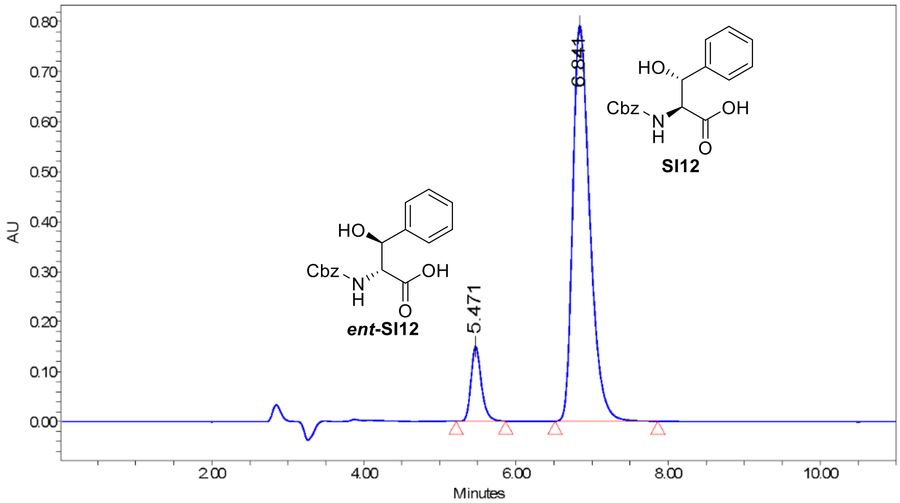

Batch_Ref_No FF.ASMJ00095.1 Empower Project: APC\Poeverlein\Poeverlein_2021

Results

	RT	Area	% Area	Height
1	5.47	1415425	10.28	149772
2	6.84	12350954	89.72	793556

Current Date 5/19/2021 Vial 31 1 of 2
Date Acquired 5/18/2021 2:57:07 PM CEST Inj Vol 5.00 uL

Sample Information:

Customer Dr. Pöverlein

Batch Ref No FF.ASMJ00095.1

SampleName FF.ASMJ00095.1

Comment

Spectrum Index Plot

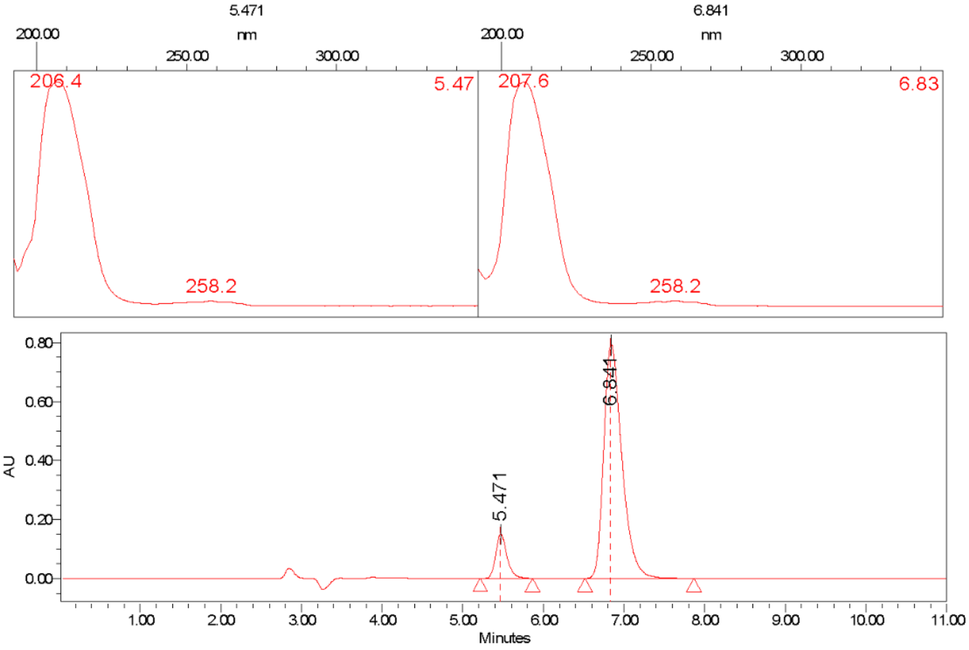

Batch_Ref_No FF.ASMJ00095.1 Project Name: APC\Poeverlein\Poeverlein_2021

Chiral Separation

Library, SM, Chiral & Peptide Purification
R&D / IDD In vitro Biology & HT Chemistry
Industriepark Höchst, G 838, Room 007
D-65926 Frankfurt
Dr. Schaffrath Tel.: +49-(0)69-305-30782

SANOFI

Sample Information:

Customer Dr. Pöverlein
Analyst K. Rahn-Hotze
Batch Ref No FF.ASMJ00091.1
Sample Name FF.ASMJ00091.1
Racemat
Lab Journal
Comment

Separation Information:

APC\Poeverlein\Poeverlein_2021
HPLC-System LC_03
Flow rate 1,0 ml/min
Temperature 30° C
HPLC Column Chiralpak IF/181, 250x4,6 mm
Eluent Hep:EtOH:MeOH 5:1:1 + 0,1% TFA

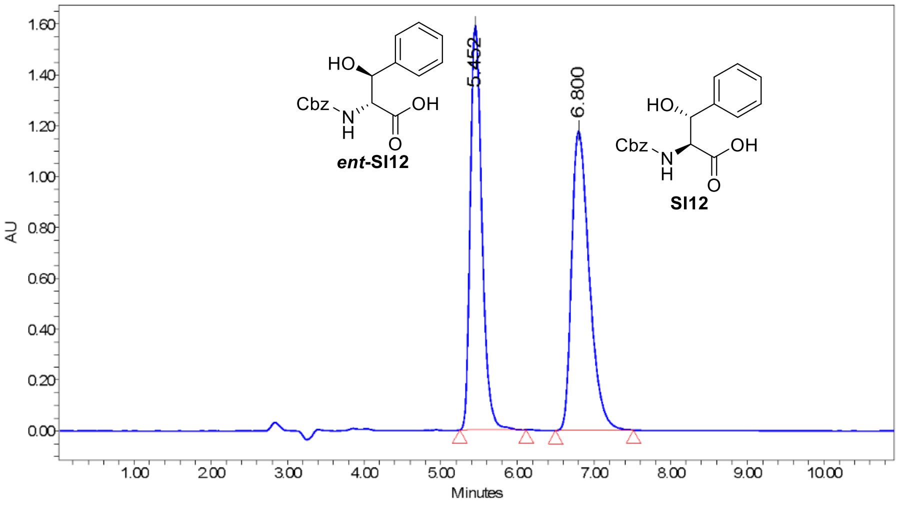

Batch_Ref_No FF.ASMJ00091.1 Empower Project: APC\Poeverlein\Poeverlein_2021

Results

	RT	Area	% Area	Height
1	5.45	16852027	47.19	1590853
2	6.80	18856420	52.81	1178025

Current Date 5/19/2021
Date Acquired 5/18/2021 9:54:32 AM CEST
Vial 28
Inj Vol 5.00 uL
1 of 2

Sample Information:

Customer Dr. Pöverlein
Batch Ref No FF.ASMJ00091.1
SampleName FF.ASMJ00091.1
Comment

Spectrum Index Plot

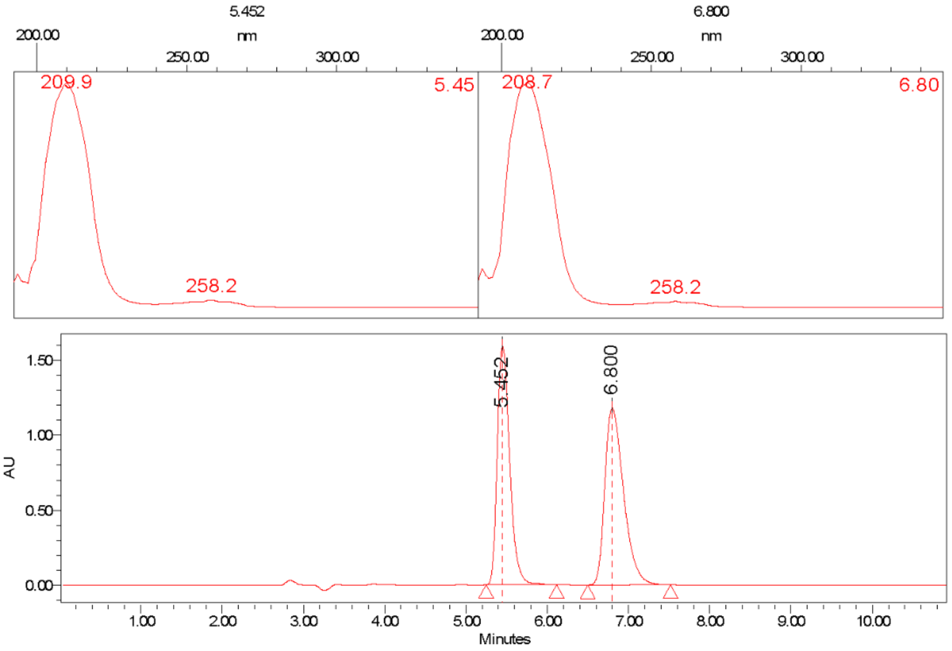

Batch_Ref_No FF.ASMJ00091.1 Project Name: APC\Poeverlein\Poeverlein_2021

Current Date 5/19/2021 Vial 28 2 of 2
Date Acquired 5/18/2021 9:54:32 AM CEST Inj Vol 5.00 uL

Chiral Separation

Library, SM, Chiral & Peptide Purification
R&D / IDD In vitro Biology & HT Chemistry
Industriepark Höchst, G 838, Room 007
D-65926 Frankfurt
Dr. Schaffrath Tel.: +49-(0)69-305-30782

SANOFI

Sample Information:

Customer	Dr. Pöverlein
Analyst	K. Rahn-Hotze
Batch Ref No	FF.ASMJ00094.1
Sample Name	FF.ASMJ00094.1
Racemat	
Lab Journal	
Comment	

Separation Information:

APC\Poeverlein\Poeverlein_2021

HPLC-System	LC_03
Flow rate	1,0 ml/min
Temperature	30° C
HPLC Column	Chiralpak IF/181, 250x4,6 mm
Eluent	Hep:EtOH:MeOH 5:1:1 + 0,1% TFA

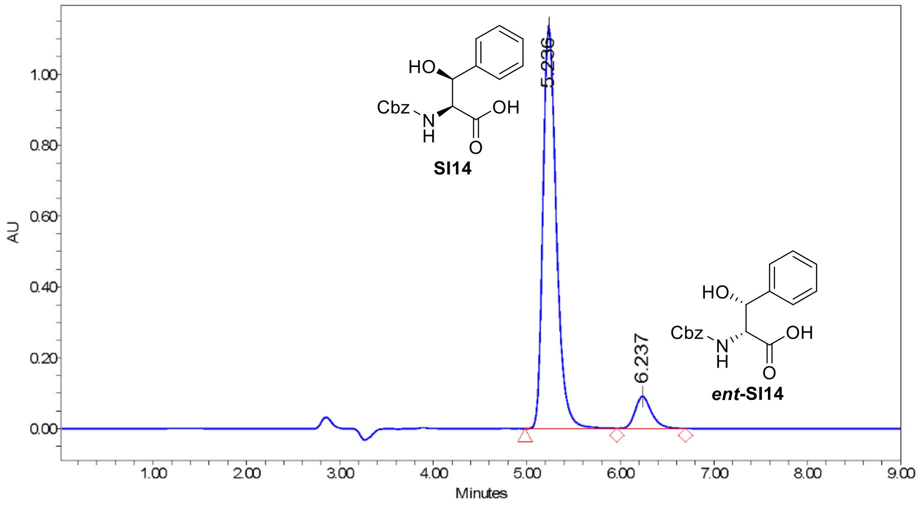

205.0nm; Inj ID 1590; Res ID 1596

Batch_Ref_No FF.ASMJ00094.1 Empower Project: APC\Poeverlein\Poeverlein_2021

Results

	RT	Area	% Area	Height
1	5.24	11346463	90.90	1137016
2	6.24	1135561	9.10	91076

Current Date 5/19/2021 Vial 30 1 of 2
Date Acquired 5/18/2021 2:47:15 PM CEST Inj Vol 5.00 uL

Sample Information:

Customer	Dr. Pöverlein
Batch Ref No	FF.ASMJ00094.1
SampleName	FF.ASMJ00094.1
Comment	

Spectrum Index Plot

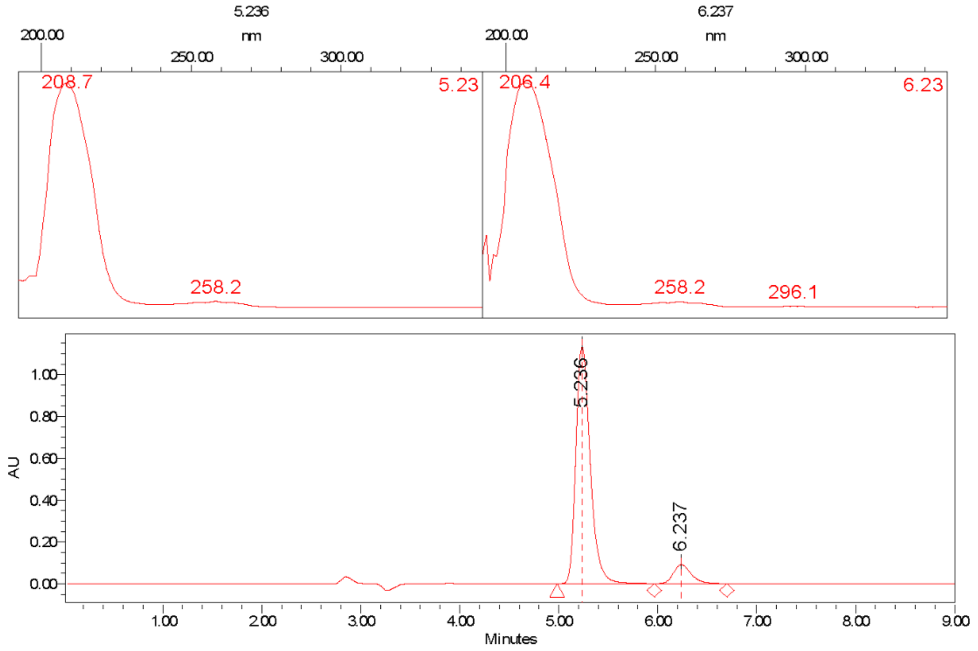

Batch_Ref_No FF.ASMJ00094.1 Project Name: APC\Poeverlein\Poeverlein_2021

Chiral Separation

Library, SM, Chiral & Peptide Purification
R&D / IDD In vitro Biology & HT Chemistry
Industriepark Höchst, G 838, Room 007
D-65926 Frankfurt
Dr. Schaffrath Tel.: +49-(0)69-305-30782

SANOFI

Sample Information:

Customer	Dr. Pöverlein
Analyst	K. Rahn-Hotze
Batch Ref No	FF.ASMJ00090.1
Sample Name	FF.ASMJ00090.1
Racemat	
Lab Journal	
Comment	

Separation Information:

APC\Poeverlein\Poeverlein_2021

HPLC-System	LC_03
Flow rate	1,0 ml/min
Temperature	30° C
HPLC Column	Chiralpak IF/181, 250x4,6 mm
Eluent	Hep:EtOH:MeOH 5:1:1 + 0,1% TFA

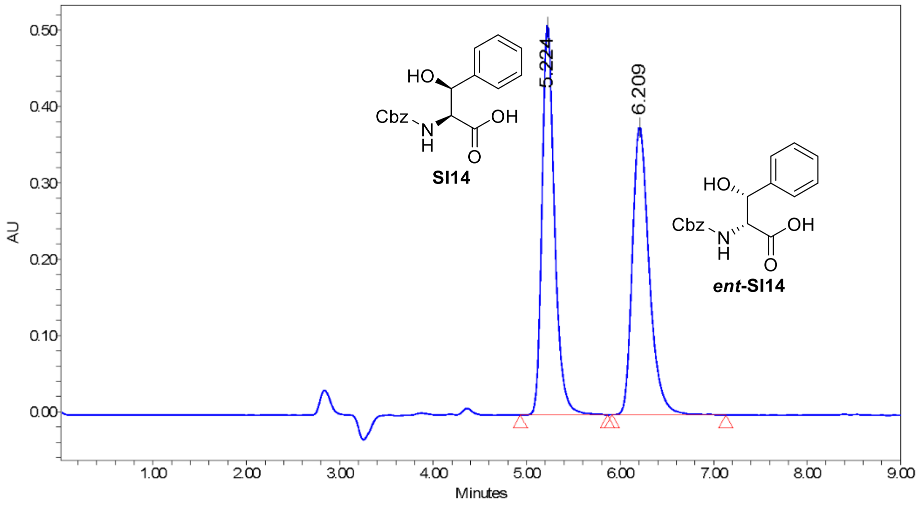

Batch_Ref_No FF.ASMJ00090.1 Empower Project: APC\Poeverlein\Poeverlein_2021

Results

	RT	Area	% Area	Height
1	5.22	4759655	50.20	513452
2	6.21	4722672	49.80	378452

Current Date 5/19/2021 Vial 27 1 of 2
Date Acquired 5/18/2021 11:37:51 AM CEST Inj Vol 5.00 uL

Sample Information:

Customer Dr. Pöverlein

Batch Ref No FF.ASMJ00090.1

SampleName FF.ASMJ00090.1

Comment

Spectrum Index Plot

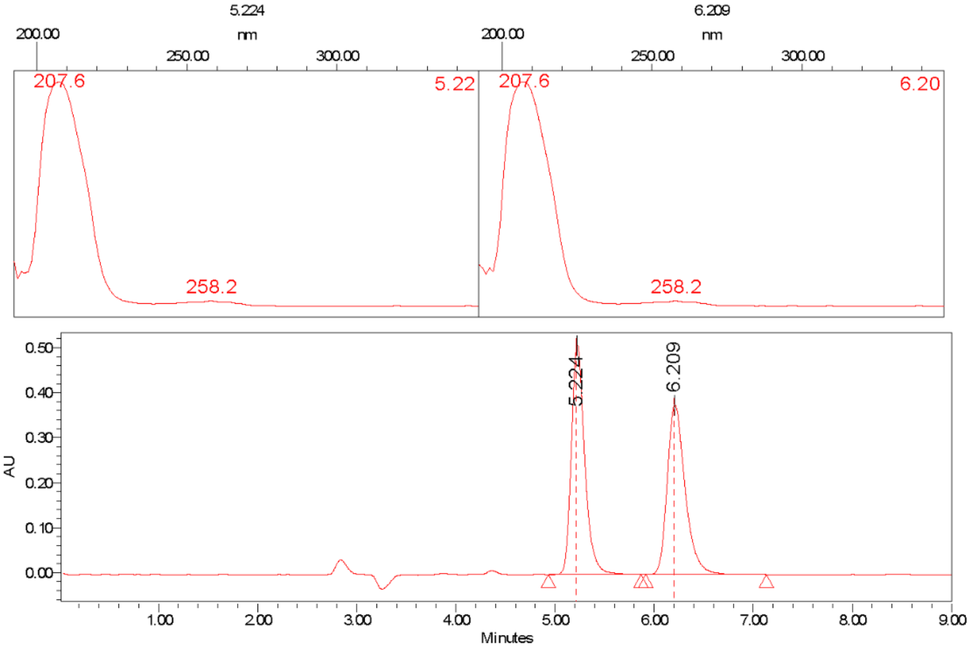

Batch_Ref_No FF.ASMJ00090.1 Project Name: APC\Poeverlein\Poeverlein_2021

Chiral Separation

Library, SM, Chiral & Peptide Purification
R&D / IDD In vitro Biology & HT Chemistry
Industriepark Höchst, G 838, Room 007
D-65926 Frankfurt
Dr. Schaffrath Tel.: +49-(0)69-305-30782

SANOFI

Sample Information:

Customer	Dr. Pöverlein
Analyst	K. Rahn-Hotze
Batch Ref No	FF.ASMJ00088.1
Sample Name	FF.ASMJ00088.1
Racemat	
Lab Journal	
Comment	

Separation Information:

APC\Poeverlein\Poeverlein_2021

HPLC-System	LC_03
Flow rate	1,0 ml/min
Temperature	30° C
HPLC Column	Chiralpak IF/181, 250x4,6 mm
Eluent	Hep:EtOH:MeOH 5:1:1 + 0,1% TFA

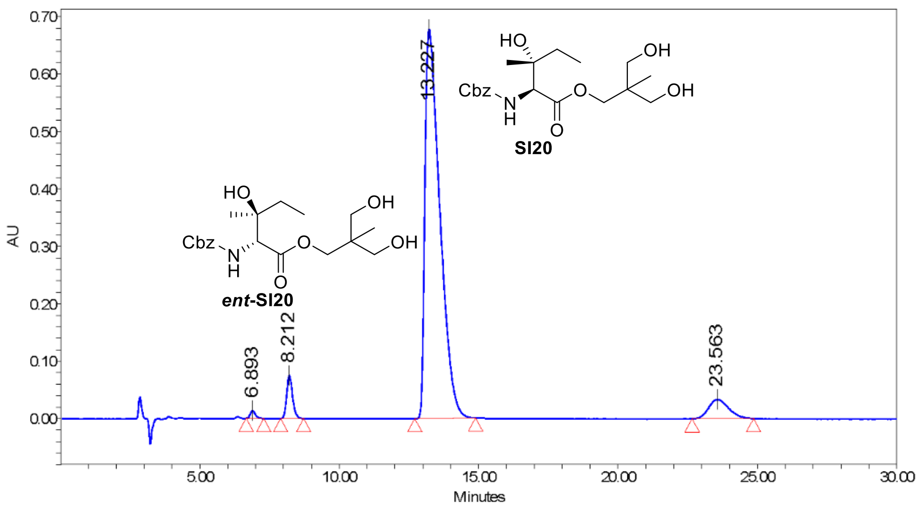

207.0nm; Inj ID 1674; Res ID 1681

Batch_Ref_No FF.ASMJ00088.1   Empower Project: APC\Poeverlein\Poeverlein_2021

Results

	RT	Area	% Area	Height
1	6.89	168888	0.60	13440
2	8.21	1103333	3.93	74819
3	13.23	25157668	89.52	677669
4	23.56	1671918	5.95	33362

Current Date 5/19/2021   Vial 37   1 of 2
Date Acquired 5/19/2021 9:06:21 AM CEST   Inj Vol 5.00 uL

Sample Information:

Customer	Dr. Pöverlein
Batch Ref No	FF.ASMJ00088.1
SampleName	FF.ASMJ00088.1
Comment	

Spectrum Index Plot

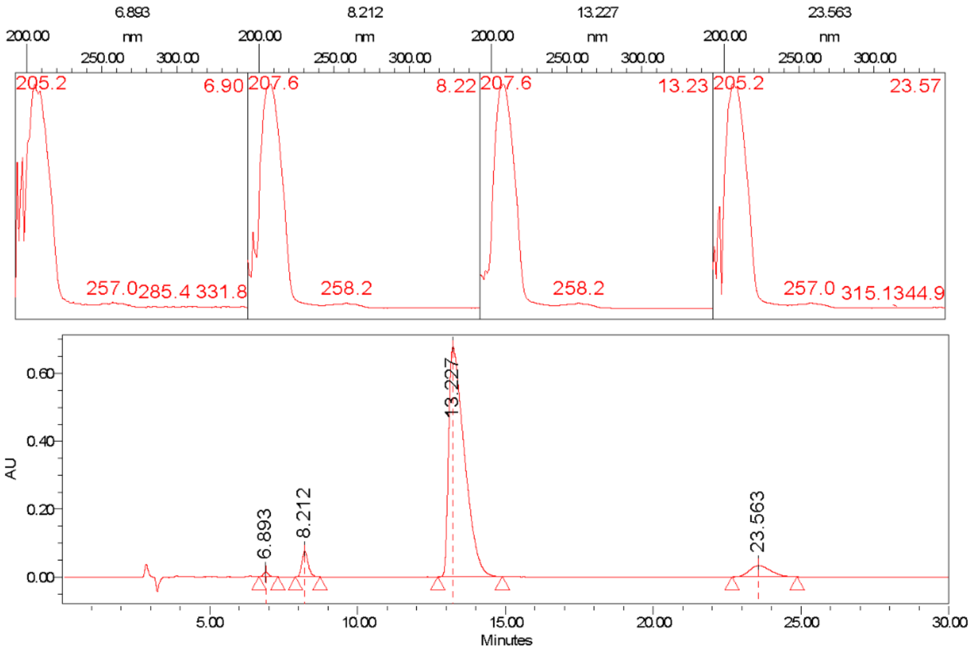

Batch_Ref_No FF.ASMJ00088.1 Project Name: APC\Poeverlein\Poeverlein_2021

Current Date	5/19/2021	Vial	37	2 of 2
Date Acquired	5/19/2021 9:06:21 AM CEST	Inj Vol	5.00 uL	

Chiral Separation

Library, SM, Chiral & Peptide Purification
R&D / IDD In vitro Biology & HT Chemistry
Industriepark Höchst, G 838, Room 007
D-65926 Frankfurt
Dr. Schaffrath Tel.: +49-(0)69-305-30782

SANOFI

Sample Information:

Customer	Dr. Pöverlein
Analyst	K. Rahn-Hotze
Batch Ref No	FF.ASMJ00079.1
Sample Name	FF.ASMJ00079.1
Racemat	
Lab Journal	
Comment	

Separation Information:

APC\Poeverlein\Poeverlein_2021

HPLC-System	LC_03
Flow rate	1,0 ml/min
Temperature	30° C
HPLC Column	Chiralpak IF/181, 250x4,6 mm
Eluent	Hep:EtOH:MeOH 5:1:1 + 0,1% TFA

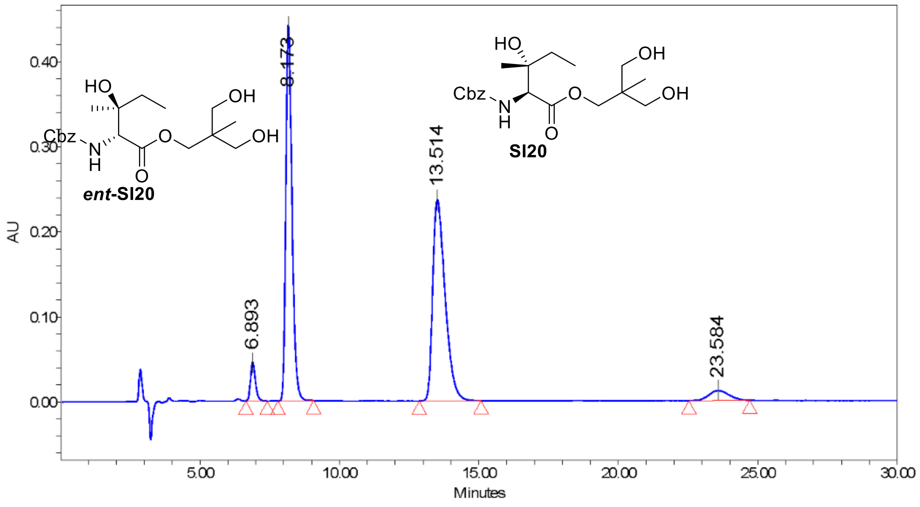

Batch_Ref_No FF.ASMJ00079.1 Empower Project: APC\Poeverlein\Poeverlein_2021

Results

	RT	Area	% Area	Height
1	6.89	557454	3.65	45324
2	8.17	6794692	44.55	442018
3	13.51	7308184	47.92	236514
4	23.58	591832	3.88	11880

Current Date 5/19/2021
Date Acquired 5/19/2021 8:04:37 AM CEST

Vial 35
Inj Vol 5.00 uL

Sample Information:

Customer Dr. Pöverlein

Batch Ref No FF.ASMJ00079.1

SampleName FF.ASMJ00079.1

Comment

Spectrum Index Plot

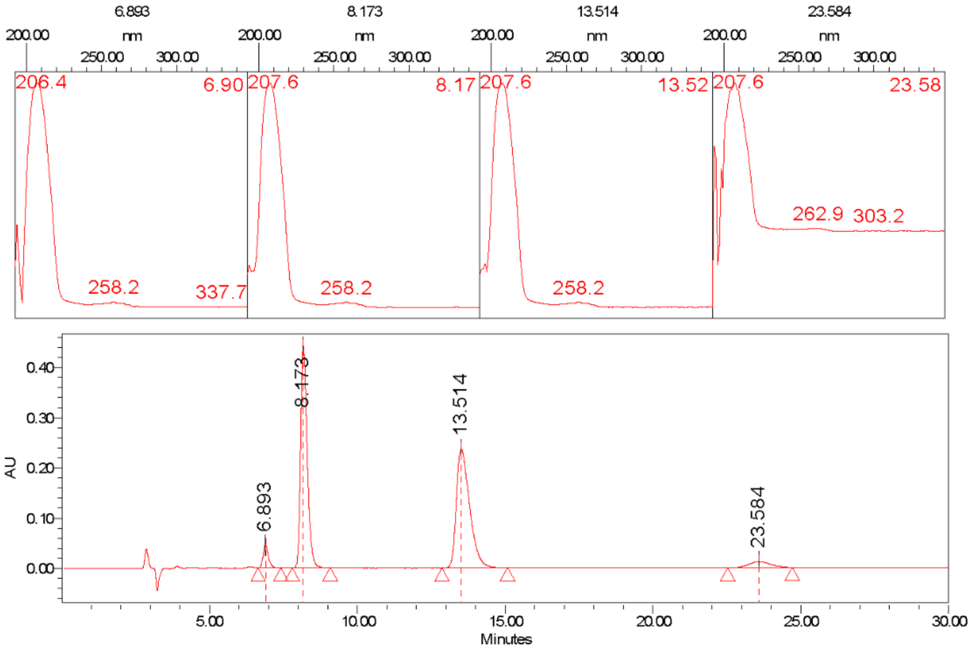

Batch_Ref_No FF.ASMJ00079.1 Project Name: APC\Poeverlein\Poeverlein_2021

Chiral Separation

Library, SM, Chiral & Peptide Purification
R&D / IDD In vitro Biology & HT Chemistry
Industriepark Höchst, G 838, Room 007
D-65926 Frankfurt
Dr. Schaffrath Tel.: +49-(0)69-305-30782

SANOFI

Sample Information:

Customer	Dr. Pöverlein
Analyst	K. Rahn-Hotze
Batch Ref No	FF.ASMJ00089.1
Sample Name	FF.ASMJ00089.1
Racemat	
Lab Journal	
Comment	

Separation Information:

APC\Poeverlein\Poeverlein_2021

HPLC-System	LC_03
Flow rate	1,0 ml/min
Temperature	30° C
HPLC Column	Chiralpak IF/181, 250x4,6 mm
Eluent	Hep:EtOH:MeOH 5:1:1 + 0,1% TFA

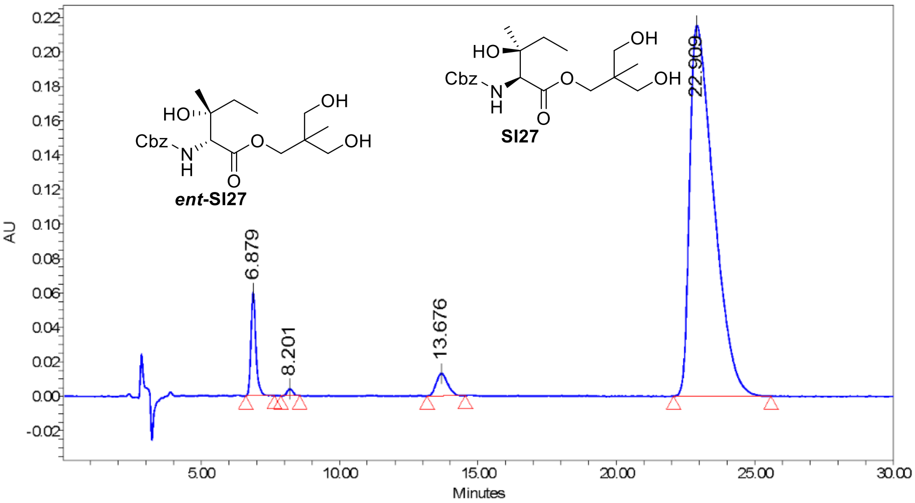

207.0nm; Inj ID 1691; Res ID 1721

Batch_Ref_No FF.ASMJ00089.1 Empower Project: APC\Poeverlein\Poeverlein_2021

Results

	RT	Area	% Area	Height
1	6.88	743457	5.24	60011
2	8.20	57360	0.40	3936
3	13.68	370942	2.62	12923
4	22.91	13004059	91.73	215289

Current Date 5/19/2021 Vial 38 1 of 2
Date Acquired 5/19/2021 11:12:48 AM CEST Inj Vol 3.00 uL

Sample Information:

Customer Dr. Pöverlein

Batch Ref No FF.ASMJ00089.1

SampleName FF.ASMJ00089.1

Comment

Spectrum Index Plot

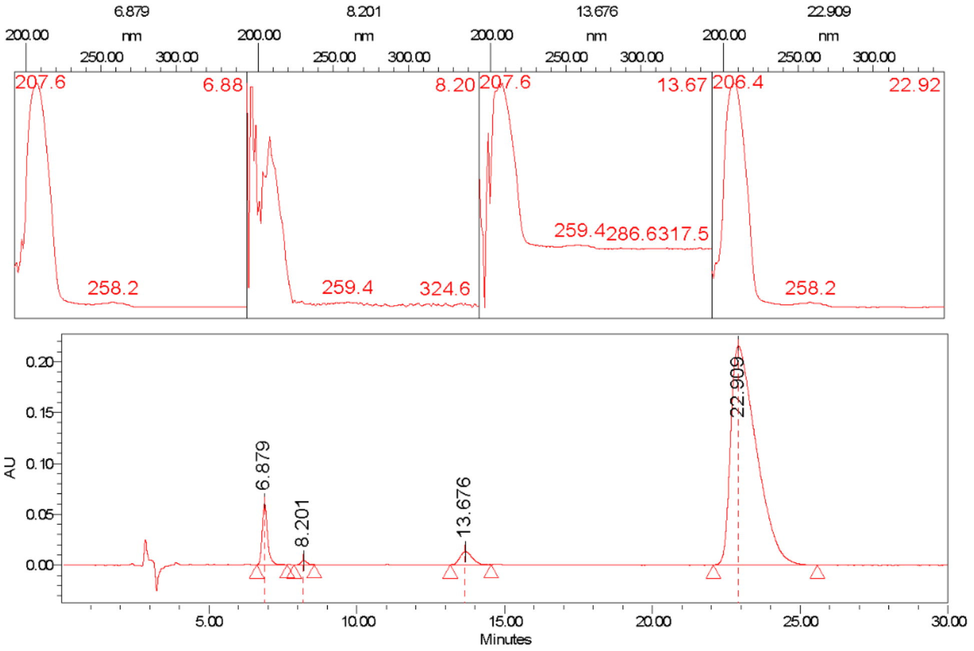

Batch_Ref_No FF.ASMJ00089.1 Project Name: APC\Poeverlein\Poeverlein_2021

Chiral Separation

Library, SM, Chiral & Peptide Purification
R&D / IDD In vitro Biology & HT Chemistry
Industriepark Höchst, G 838, Room 007
D-65926 Frankfurt
Dr. Schaffrath Tel.: +49-(0)69-305-30782

SANOFI

Sample Information:

Customer	Dr. Pöverlein
Analyst	K. Rahn-Hotze
Batch Ref No	FF.ASMJ00080.1
Sample Name	FF.ASMJ00080.1
Racemat	
Lab Journal	
Comment	

Separation Information:

APC\Poeverlein\Poeverlein_2021

HPLC-System	LC_03
Flow rate	1,0 ml/min
Temperature	30° C
HPLC Column	Chiralpak IF/181, 250x4,6 mm
Eluent	Hep:EtOH:MeOH 5:1:1 + 0,1% TFA

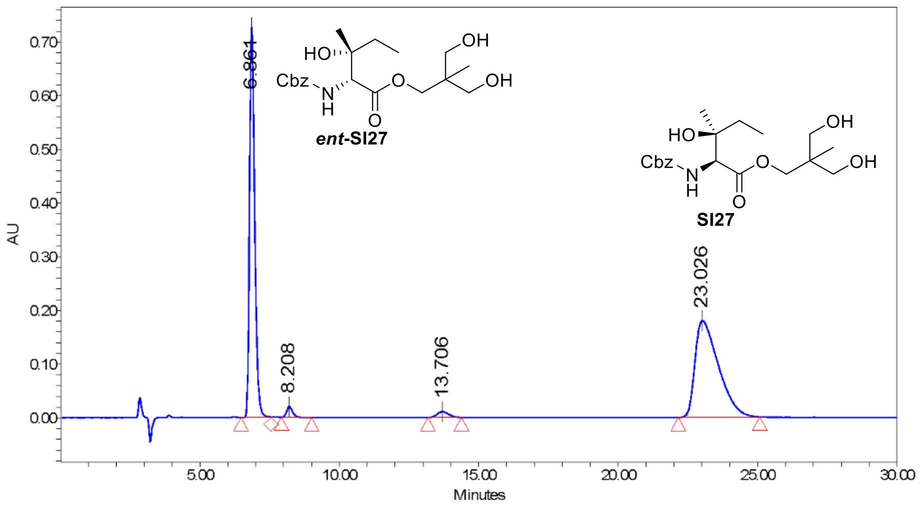

Batch_Ref_No FF.ASMJ00080.1 Empower Project: APC\Poeverlein\Poeverlein_2021

Results

	RT	Area	% Area	Height
1	6.86	9352186	45.72	727535
2	8.21	294051	1.44	19947
3	13.71	306613	1.50	10859
4	23.03	10503751	51.35	179849

Current Date 5/19/2021
Date Acquired 5/19/2021 8:35:28 AM CEST
Vial 36
Inj Vol 5.00 uL

Sample Information:

Customer Dr. Pöverlein

Batch Ref No FF.ASMJ00080.1

SampleName FF.ASMJ00080.1

Comment

Spectrum Index Plot

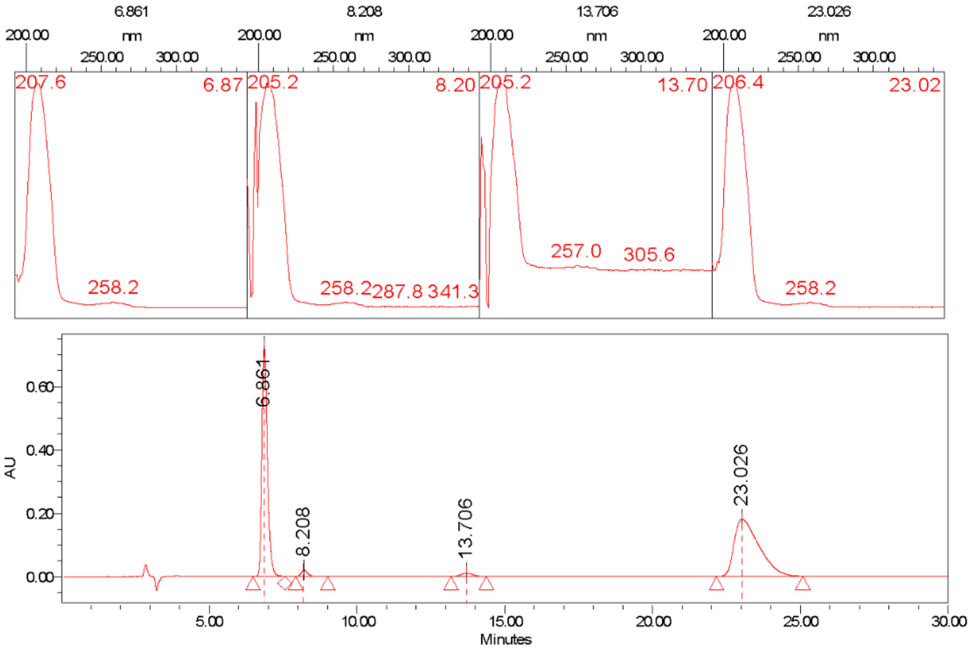

Batch_Ref_No FF.ASMJ00080.1 Project Name: APC\Poeverlein\Poeverlein_2021

Current Date 5/19/2021 Vial 36 2 of 2

Date Acquired 5/19/2021 8:35:28 AM CEST Inj Vol 5.00 uL

Chiral Separation

Library, SM, Chiral & Peptide Purification
R&D / IDD In vitro Biology & HT Chemistry
Industriepark Höchst, G 838, Room 007
D-65926 Frankfurt
Dr. Schaffrath Tel.: +49-(0)69-305-30782

SANOFI

Sample Information:

Customer Dr. Pöverlein
Analyst K. Rahn-Hotze
Batch Ref No FF.ASMJ00096.1
Sample Name FF.ASMJ00096.1
Racemat
Lab Journal
Comment

Separation Information:

APC\Poeverlein\Poeverlein_2021
HPLC-System LC_03
Flow rate 1,0 ml/min
Temperature 30° C
HPLC Column Chiralpak IF/181, 250x4,6 mm
Eluent Hep:EtOH 10:1 + 0,1% TFA

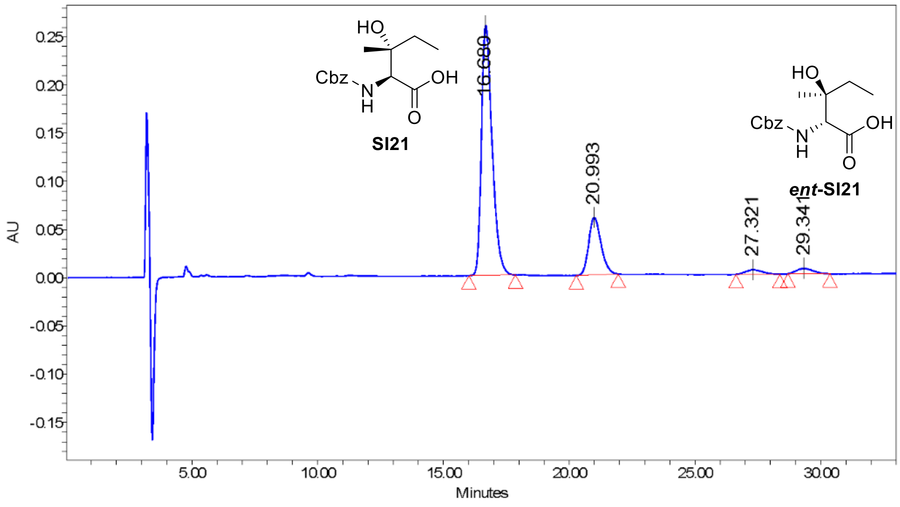

Batch_Ref_No FF.ASMJ00096.1 Empower Project: APC\Poeverlein\Poeverlein_2021

Results

	RT	Area	% Area	Height
1	16.68	7643383	75.21	259347
2	20.99	2040213	20.07	59600
3	27.32	219056	2.16	4734
4	29.34	260346	2.56	5668

Current Date 6/8/2021 Vial 44 1 of 2
Date Acquired 6/8/2021 10:08:03 AM CEST Inj Vol 5.00 uL

Sample Information:

Customer Dr. Pöverlein

Batch Ref No FF.ASMJ00096.1

SampleName FF.ASMJ00096.1

Comment

Spectrum Index Plot

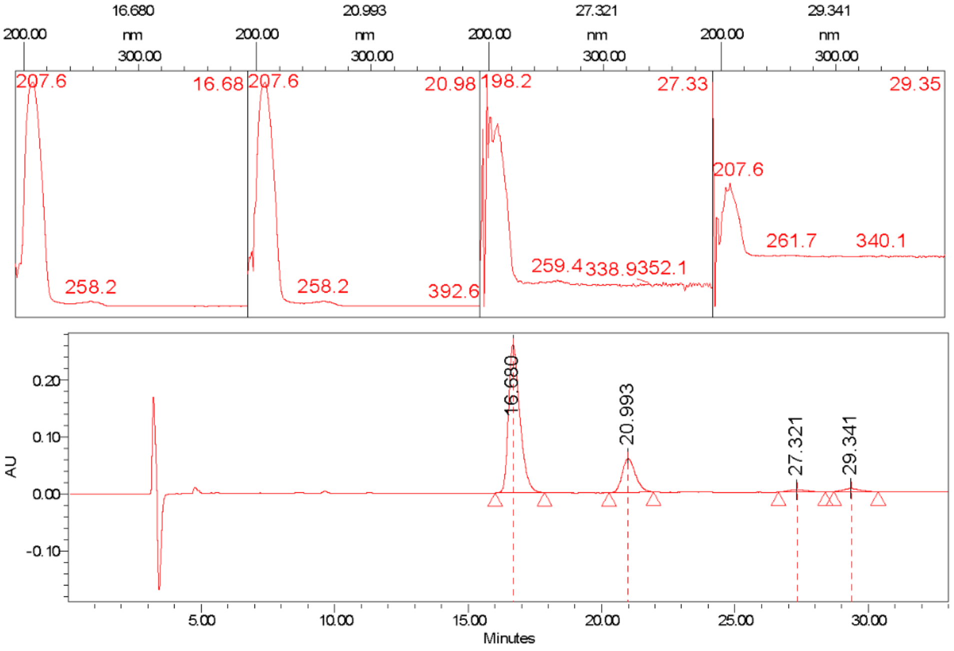

Batch_Ref_No FF.ASMJ00096.1 Project Name: APC\Poeverlein\Poeverlein_2021

Chiral Separation

Library, SM, Chiral & Peptide Purification
R&D / IDD In vitro Biology & HT Chemistry
Industriepark Höchst, G 838, Room 007
D-65926 Frankfurt
Dr. Schaffrath Tel.: +49-(0)69-305-30782

SANOFI

Sample Information:

Customer Dr. Pöverlein
Analyst K. Rahn-Hotze
Batch Ref No FF.ASMJ00092.1
Sample Name FF.ASMJ00092.1
Racemat
Lab Journal
Comment

Separation Information:

APC\Poeverlein\Poeverlein_2021
HPLC-System LC_03
Flow rate 1,0 ml/min
Temperature 30° C
HPLC Column Chiralpak IF/181, 250x4,6 mm
Eluent Hep:EtOH 10:1 + 0,1% TFA

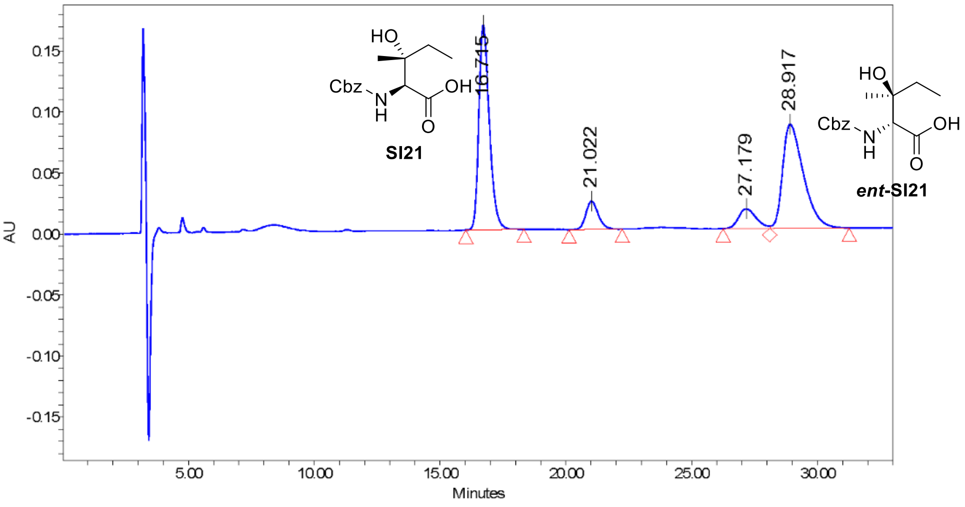

208.0nm; Inj ID 1774; Res ID 1783

Batch_Ref_No FF.ASMJ00092.1 Empower Project: APC\Poeverlein\Poeverlein_2021

Results

	RT	Area	% Area	Height
1	16.72	4876070	42.95	167660
2	21.02	803425	7.08	23173
3	27.18	787898	6.94	16162
4	28.92	4884366	43.03	85118

Current Date 6/8/2021
Date Acquired 6/8/2021 9:00:19 AM CEST
Vial 42
Inj Vol 5.00 uL
1 of 2

Sample Information:

Customer Dr. Pöverlein

Batch Ref No FF.ASMJ00092.1

SampleName FF.ASMJ00092.1

Comment

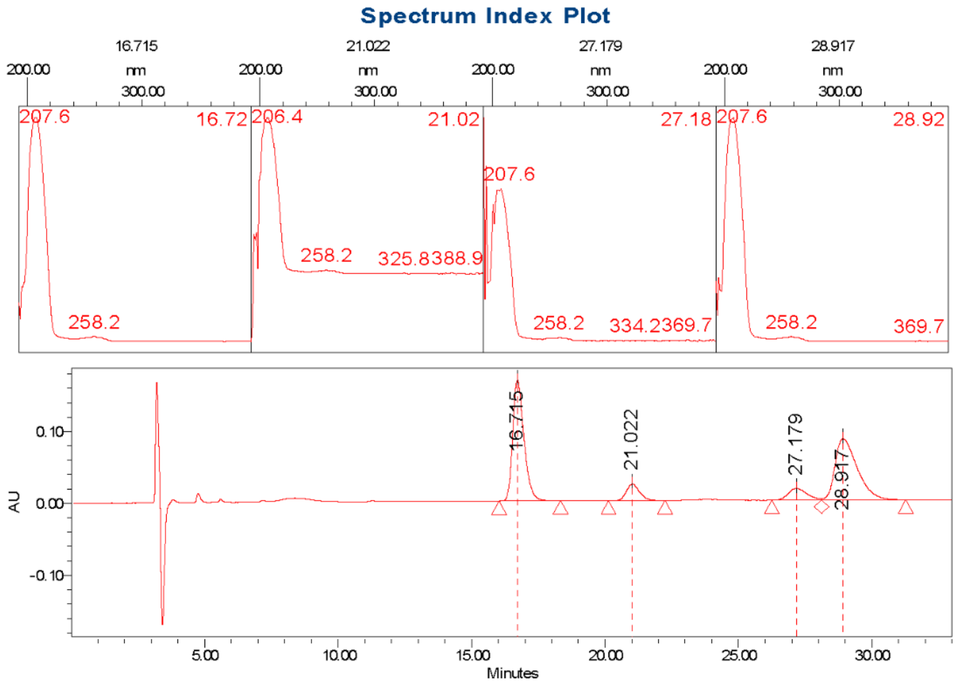

Batch_Ref_No FF.ASMJ00092.1 Project Name: APC\Poeverlein\Poeverlein_2021

Chiral Separation

Library, SM, Chiral & Peptide Purification
R&D / IDD In vitro Biology & HT Chemistry
Industriepark Höchst, G 838, Room 007
D-65926 Frankfurt
Dr. Schaffrath Tel.: +49-(0)69-305-30782

SANOFI

Sample Information:

Customer	Dr. Pöverlein
Analyst	K. Rahn-Hotze
Batch Ref No	FF.ASMJ00097.1
Sample Name	FF.ASMJ00097.1
Racemat	
Lab Journal	
Comment	

Separation Information:

APC\Poeverlein\Poeverlein_2021

HPLC-System	LC_03
Flow rate	1,0 ml/min
Temperature	30° C
HPLC Column	Chiralpak IF/181, 250x4,6 mm
Eluent	Hep:EtOH 10:1 + 0,1% TFA

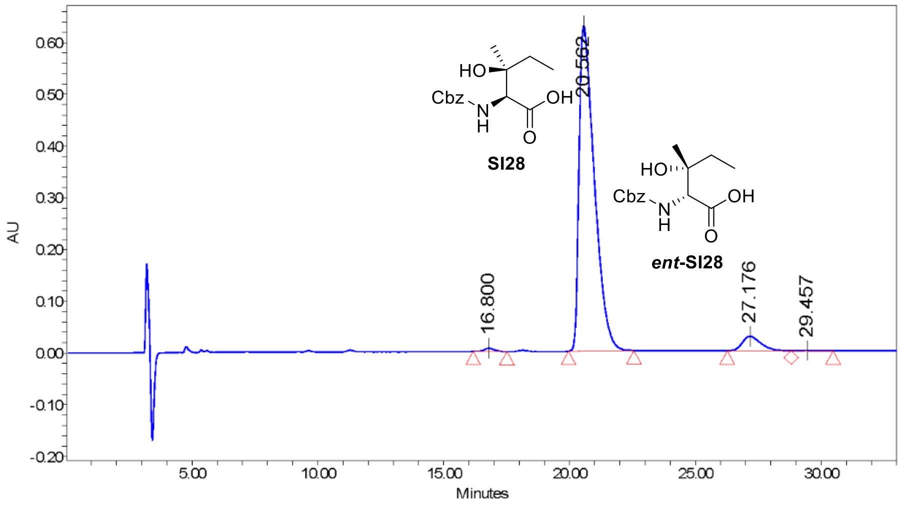

Batch_Ref_No FF.ASMJ00097.1 Empower Project: APC\Poeverlein\Poeverlein_2021

Results

	RT	Area	% Area	Height
1	16.80	165940	0.59	6167
2	20.56	26569318	94.16	629039
3	27.18	1444362	5.12	28117
4	29.46	37232	0.13	730

Current Date 6/8/2021
Date Acquired 6/8/2021 10:41:54 AM CEST
Vial 45
Inj Vol 5.00 uL

Sample Information:

Customer	Dr. Pöverlein
Batch Ref No	FF.ASMJ00097.1
SampleName	FF.ASMJ00097.1
Comment	

Spectrum Index Plot

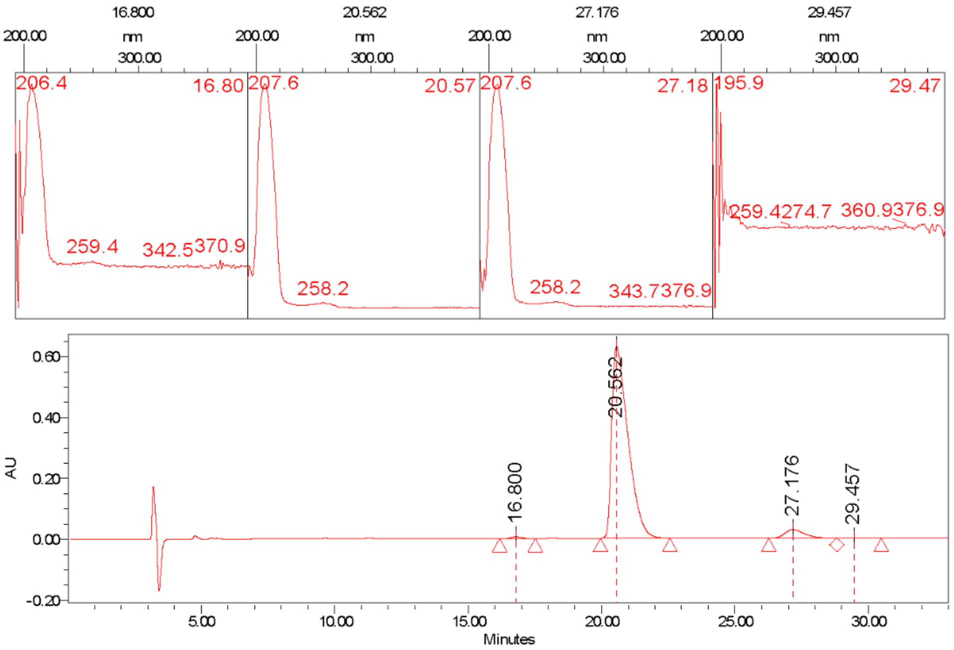

Batch_Ref_No FF.ASMJ00097.1 Project Name: APC\Poeverlein\Poeverlein_2021

Chiral Separation

Library, SM, Chiral & Peptide Purification
R&D / IDD In vitro Biology & HT Chemistry
Industriepark Höchst, G 838, Room 007
D-65926 Frankfurt
Dr. Schaffrath Tel.: +49-(0)69-305-30782

SANOFI

Sample Information:

Customer	Dr. Pöverlein
Analyst	K. Rahn-Hotze
Batch Ref No	FF.ASMJ00093.1
Sample Name	FF.ASMJ00093.1
Racemat	
Lab Journal	
Comment	

Separation Information:

APC\Poeverlein\Poeverlein_2021

HPLC-System	LC_03
Flow rate	1,0 ml/min
Temperature	30° C
HPLC Column	Chiralpak IF/181, 250x4,6 mm
Eluent	Hep:EtOH 10:1 + 0,1% TFA

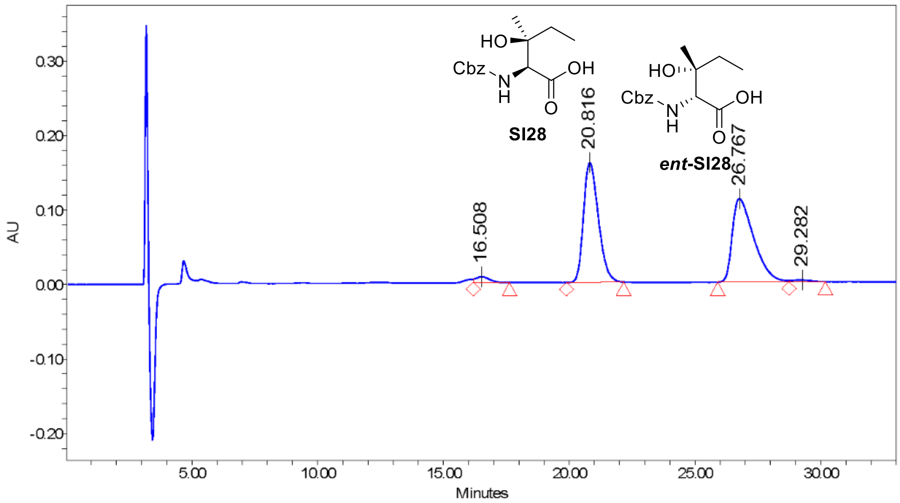

Batch_Ref_No FF.ASMJ00093.1 Empower Project: APC\Poeverlein\Poeverlein_2021

Results

	RT	Area	% Area	Height
1	16.51	288213	2.11	7812
2	20.82	6602833	48.38	160649
3	26.77	6642495	48.67	111814
4	29.28	115445	0.85	2267

Current Date 6/8/2021
Date Acquired 6/8/2021 11:17:59 AM CEST

Vial 43
Inj Vol 15.00 uL

Sample Information:

Customer Dr. Pöverlein

Batch Ref No FF.ASMJ00093.1

SampleName FF.ASMJ00093.1

Comment

Spectrum Index Plot

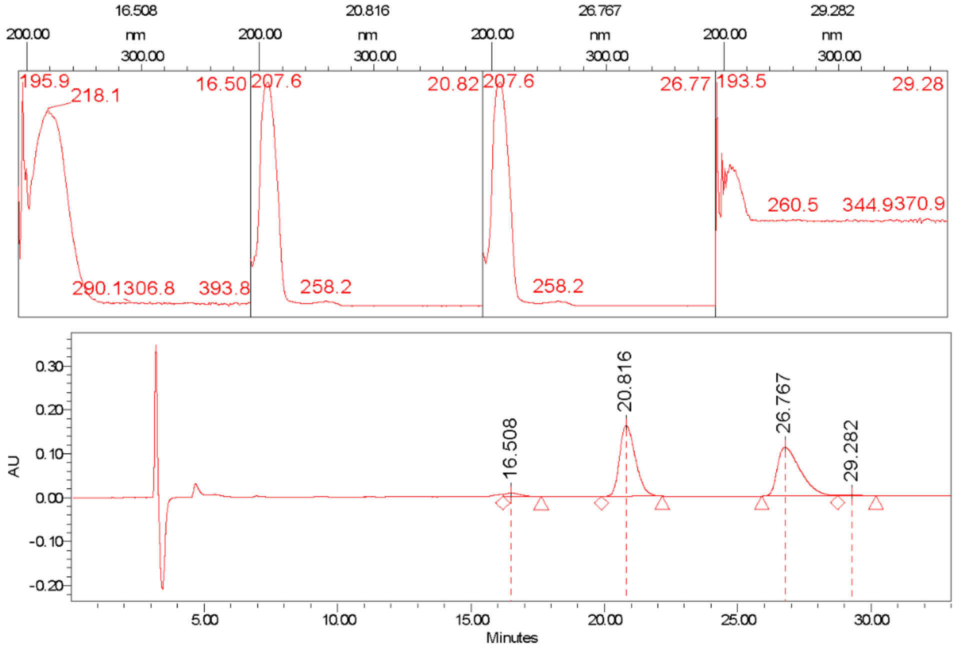

Batch_Ref_No FF.ASMJ00093.1 Project Name: APC\Poeverlein\Poeverlein_2021
